# Ancestral reconstruction reveals catalytic inactivation of activation-induced cytidine deaminase concomitant with cold water adaption in the Gadiformes bony fish

**DOI:** 10.1186/s12915-022-01489-8

**Published:** 2022-12-27

**Authors:** Atefeh Ghorbani, S. Javad Khataeipour, Monica H. Solbakken, David N. G. Huebert, Minasadat Khoddami, Khalil Eslamloo, Cassandra Collins, Tiago Hori, Sissel Jentoft, Matthew L. Rise, Mani Larijani

**Affiliations:** 1 Department of Molecular Biology and Biochemistry, Simon Fraser University, Burnaby, Canada; 2 Program in Immunology and Infectious Diseases, Division of Biomedical Sciences, Faculty of Medicine, Memorial University of Newfoundland, St. John’s, Canada; 3 Department of Computer Science, Faculty of Science, Memorial University of Newfoundland, St. John’s, Canada; 4 Centre for Ecological and Evolutionary Synthesis, Department of Biosciences, University of Oslo, Oslo, Norway; 5 Department of Ocean Sciences, Memorial University of Newfoundland, St. John’s, Canada

**Keywords:** Antibody evolution, Ancestral enzymes, DNA editing, Innate and adaptive immunity, Enzyme evolution

## Abstract

**Background:**

Antibody affinity maturation in vertebrates requires the enzyme activation-induced cytidine deaminase (AID) which initiates secondary antibody diversification by mutating the immunoglobulin loci. AID-driven antibody diversification is conserved across jawed vertebrates since bony and cartilaginous fish. Two exceptions have recently been reported, the Pipefish and Anglerfish, in which the AID-encoding *aicda* gene has been lost. Both cases are associated with unusual reproductive behavior, including male pregnancy and sexual parasitism. Several cold water fish in the Atlantic cod (Gadinae) family carry an *aicda* gene that encodes for a full-length enzyme but lack affinity-matured antibodies and rely on antibodies of broad antigenic specificity. Hence, we examined the functionality of their AID.

**Results:**

By combining genomics, transcriptomics, immune responsiveness, and functional enzymology of AID from 36 extant species, we demonstrate that AID of that Atlantic cod and related fish have extremely lethargic or no catalytic activity. Through ancestral reconstruction and functional enzymology of 71 AID enzymes, we show that this enzymatic inactivation likely took place relatively recently at the emergence of the true cod family (Gadidae) from their ancestral Gadiformes order. We show that this AID inactivation is not only concordant with the previously shown loss of key adaptive immune genes and expansion of innate and cell-based immune genes in the Gadiformes but is further reflected in the genomes of these fish in the form of loss of AID-favored sequence motifs in their immunoglobulin variable region genes.

**Conclusions:**

Recent demonstrations of the loss of the *aicda* gene in two fish species challenge the paradigm that AID-driven secondary antibody diversification is absolutely conserved in jawed vertebrates. These species have unusual reproductive behaviors forming an evolutionary pressure for a certain loss of immunity to avoid tissue rejection. We report here an instance of catalytic inactivation and functional loss of AID rather than gene loss in a conventionally reproducing vertebrate. Our data suggest that an expanded innate immunity, in addition to lower pathogenic pressures in a cold environment relieved the pressure to maintain robust secondary antibody diversification. We suggest that in this unique scenario, the AID-mediated collateral genome-wide damage would form an evolutionary pressure to lose AID function.

**Supplementary Information:**

The online version contains supplementary material available at 10.1186/s12915-022-01489-8.

## Background

Classical mammalian antibody affinity maturation (AM) appears to be a conserved feature of the jawed vertebrate adaptive immune response. Many studies have revealed the presence of the antibody immune response and AM prior to divergence of cartilaginous and bony fish [1–17]. As several specific examples, affinity maturation has been detected in IgM and IgNAR of immunized nurse shark (*Ginglymostoma cirratum*), improving antibody-antigen binding affinity by up to tenfold [9, 11]. In the rainbow trout (*Oncorhynchus mykiss*), the emergence of higher affinity antibodies by week 14 after immunization has been reported [7, 8]. In immunized Atlantic salmon (*Salmo salar*), up to a tenfold increase in antibody affinity has been observed [18]. Also, in the South African clawed toad (*Xenopus laevis*), 5- to tenfold increase in antibody affinity was detected 4 weeks after immunization [15].

AM requires somatic hypermutation (SHM) of immunoglobulin (Ig) genes by the enzyme activation-induced cytidine deaminase (AID, encoded by *aicda* gene) [19–24]. AID is expressed in activated B lymphocytes where it converts deoxycytidine (dC) to deoxyuridine (dU) at *Ig* variable (V) and switch (S) sequences, preferentially in the context of WRC (W = A/T; R = A/G) motifs [19, 21, 22, 25]. AID deficiency in mouse (*Mus musculus*) and human (*Homo sapiens*) results in hyper-IgM (HIGM) immunodeficiency characterized by lack of affinity-matured and isotype-switched antibodies [26, 27]. AID-mediated SHM has been reported in *IgV* genes of immunized *Xenopus*, channel catfish (*Ictalurus punctatus*), zebrafish (*Danio rerio*), and the nurse shark [11–16]. Based on the presence of the recombination activating gene products (RAGs1/2), classical V(D)J-based antibody genes, and the AID-mediated SHM as far back as the cartilaginous fish, and in many studied bony fish, until recently it was believed that AID-mediated secondary antibody diversification and antibody affinity maturation are universal in jawed vertebrates. Further solidifying this model have been the findings of AID-related cytidine deaminases encoded in the genomes of several invertebrates, though the roles that they may play in immunity remains unknown [28, 29]. However, two recent studies have challenged the universal conservation of AID-mediated secondary antibody diversification in jawed vertebrates by showing that there are at least two species of bony fish, the Anglerfish and Pipefish that lack the *aicda* gene [30, 31]. These fish have alternative reproductive behaviors, in the form of sexual parasitism and male pregnancy presumably necessitating a certain loss of robust immunity to avoid tissue rejection, potentially explaining the loss of AID.

Recent studies have also revealed a loss of key immune genes from the genomes of the Gadiformes (related to the Atlantic cod) family of bony fish where key genes involved in B and helper T cell activation (i.e., *mhc II* and *cd4*) are lost, whereas genes involved in cell-mediated and innate immunity (i.e., *mhc I* and *tlrs*) are expanded [32, 33]. Prior studies demonstrated that the antibody response of Atlantic cod (*Gadus morhua*) and haddock (*Melanogrammus aeglefinus*)—both members of the Gadiformes lineage—lacked affinity-matured antibodies [18, 34–37]. These scenarios are similar to that of HIGM syndrome mediated by AID deficiency prompting us to investigate the genetics, functionality, and evolutionary trajectory of AID within Gadiformes lineage.

We examined the genetics and transcription of Atlantic cod AID and characterized the biochemical properties of 71 extant, predicted ancestral, and mutant AIDs within and outside of Gadiformes lineage. This study reveals an instance of purposeful catalytic inactivation of AID in vertebrates that are not only not immunodeficient, but among the most successful and thriving species in their habitats. We have applied ancestral sequence reconstruction (ASR) to an enzyme involved in the immune system to discover the evolutionary juncture and genetic events surrounding this natural instance of enzyme inactivation.

## Results

### Aicda gene synteny and transcription profile in the Atlantic cod

Since the Atlantic cod is the most studied Gadiformes species with an available genome assembly [38], we investigated the chromosomal location and transcript expression of its *aicda* gene. The Atlantic cod genome project has revealed a putative *aicda* gene with a 5-exon genomic structure which is conserved across examined vertebrates (Supplementary Fig. 1) [38, 39]. Upon the examination of the *aicda* gene, we observed that *aicda* gene has a conserved synteny within Teleostei and Amniota (mammals, reptiles, and birds) groups (Supplementary Fig. 2). These results suggest that during the teleost-specific whole-genome duplication (TS-WGD) event [40], a different copy of the *aicda* has been retained in teleost species compared to the tetrapod group.

**Fig. 1:**
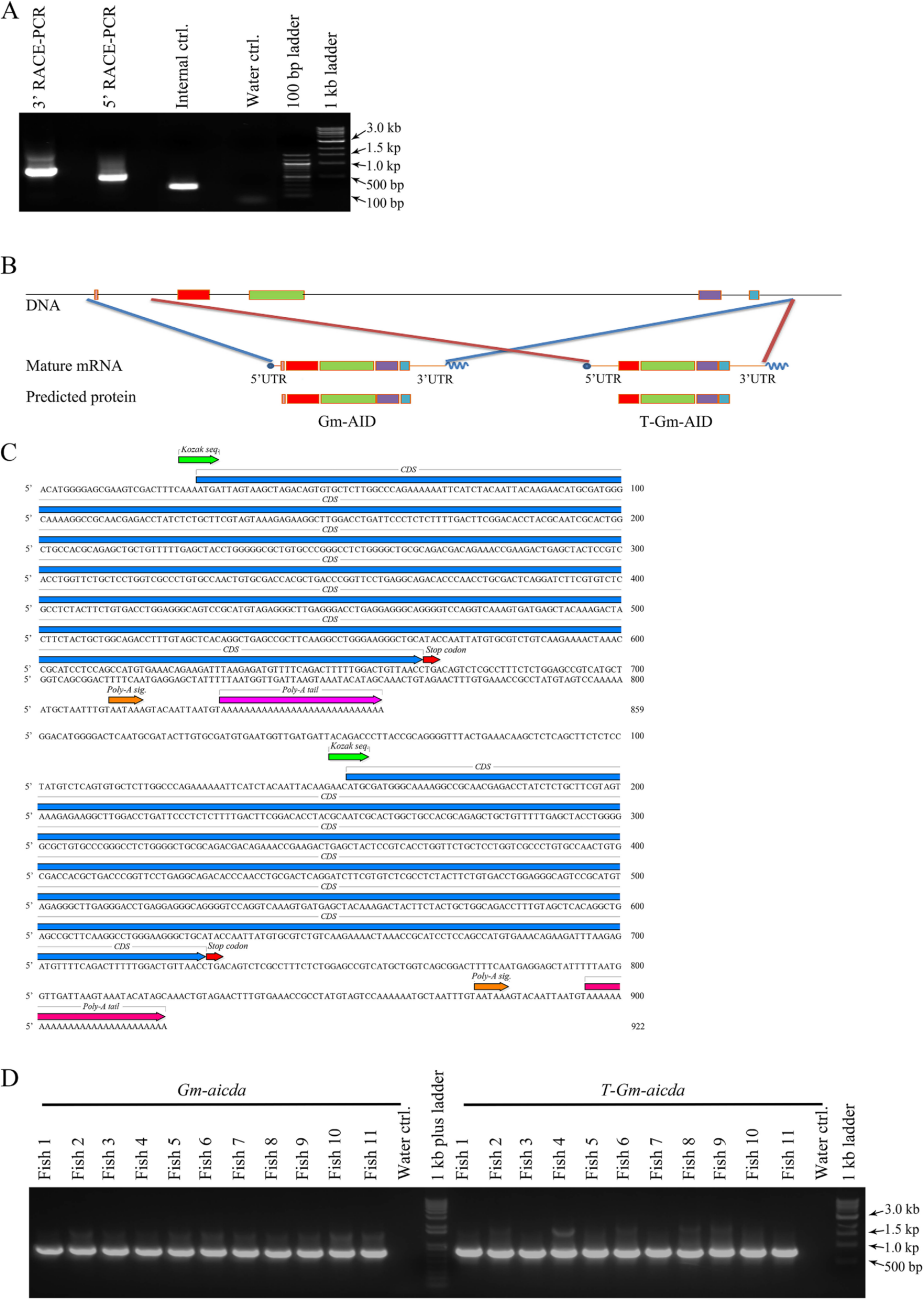
Identification and characterization of *aicda* transcript(s) in Atlantic cod.** A** Amplification of full-length AID mRNA(s) through RACE-PCR using 1 µg of splenic RNA. **B** Analyses of the RACE-PCR sequencing data revealed two mRNA transcripts encoding a full-length *aicda* and a truncated isoform. **C** The ATGpr website was used to identify the initiation codon, coding sequence (CDS), and the stop codon with highest probability. The Kozak sequence, CDS, stop codon, poly-A signal, and poly-A tail are labelled for each identified mRNA sequence. Comparison of the *Gm-aicda* genomic region and identified transcripts showed different transcription start site utilization among the two transcripts resulting in the absence of the first exon in the truncated transcript (*T*-*Gm-*aicda). **D** Confirmation of the presence of both *aicda* transcripts in Atlantic cod individuals through RT-PCR. One microgram of splenic total RNA of each pIC-stimulated fish sampled at 24 HPI was used for cDNA synthesis. Eleven individual fish were included. Isoform-specific primers (ISPs) and 2.5 µl of diluted cDNA (equivalent to 25 ng of initial RNA) were used to amplify both transcripts. The amplicons were sequenced to confirm the results

Through rapid amplification of cDNA ends (RACE) PCR, we found two distinct *aicda* transcripts in Atlantic cod (Fig. 1A). The 830-bp transcript contains a 642-bp coding sequence and encodes a full-length AID. The 892-bp transcripts lack the first exon and encode a truncated AID isoform in which the first 21 amino acids are missing (Fig. 1B, C, Supplementary Fig. 1). The full-length and truncated versions, hereafter respectively referred to as *Gm-aicda* and *T-Gm-aicda*, share the same 3′-UTR in which the polyadenylation signal (AAUAAA) is located 13 bp upstream of the poly-A tail (Fig. 1B, C). Amino acid alignment of AID homologs revealed that the encoded Gm-AID protein from the full-length transcript contains all of AID’s hallmark functional motifs, including the Zn-coordinating and catalytic residues, secondary catalytic residues, nuclear localization signal, nuclear export signal, and phosphorylation sites) [41–48]. Within the AID/APOBEC family, the core catalytic motif is comprised of H[A/V]E-X[24-36]-PCXXC motif in which the histidine (H) and the two cysteines (C) coordinate the catalytic Zn^2+^ and the glutamate (E) acts as proton donor in the deamination reaction [49] (Fig. 1B, Supplementary Fig. 1). We then examined 11 individual fish and found both transcripts expressed in splenic cDNA of all fish (Fig. 1D). Previous studies have reported the presence of distinct *aicda* isoforms, due to utilizing different poly-A sites or alternative splicing, in Iberian ribbed newt, African clawed frog, mouse, and human but not in channel catfish, zebrafish, domestic dog, and cattle [24, 50–58]. A similarly truncated *aicda* transcript like *T-Gm-aicda* has only been reported in the Iberian ribbed newt [56] occurring due to a different transcription start site, suggesting involvement of different transcription factors.

**Fig. 2:**
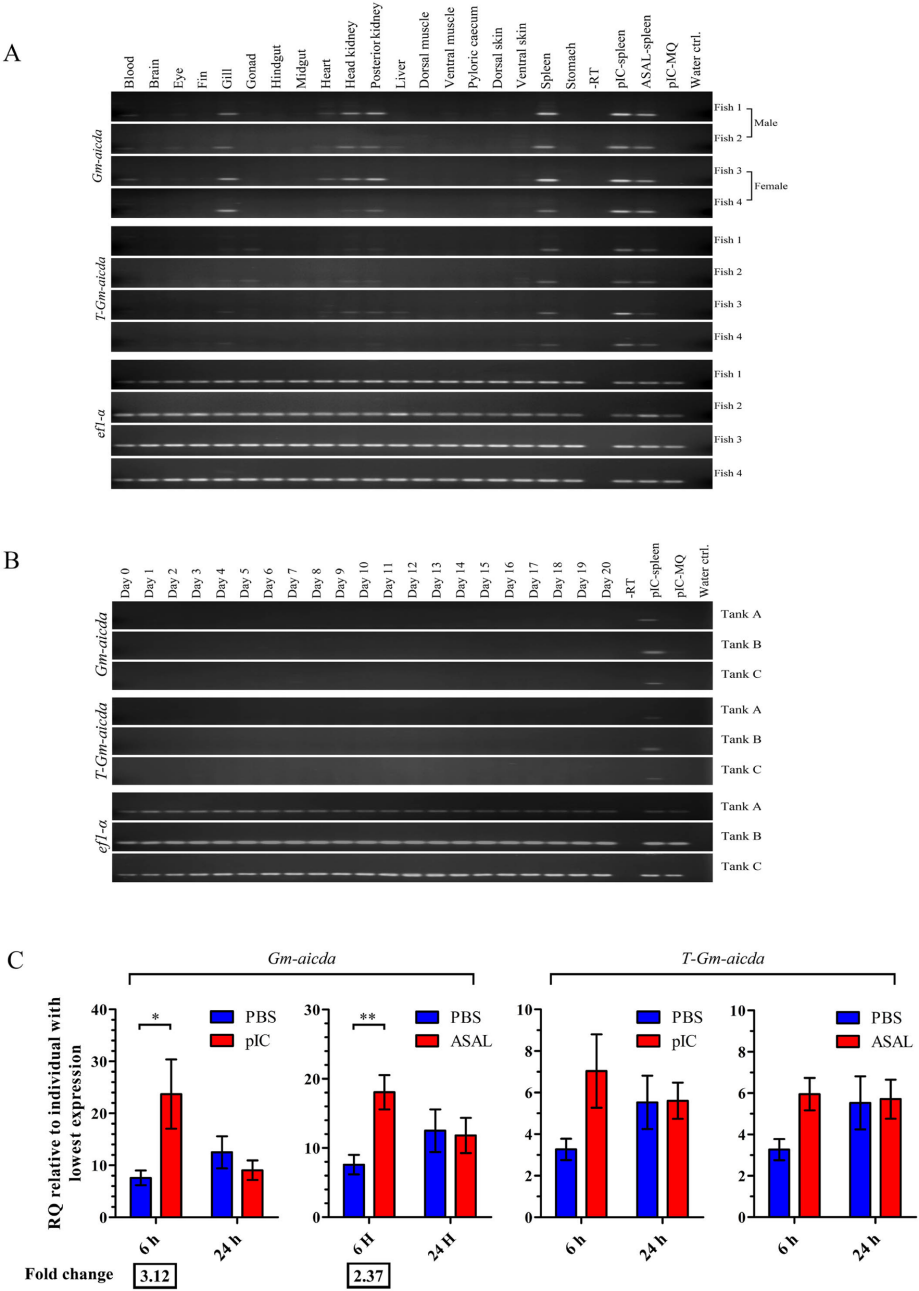
Tissue-specific expression profile of the Atlantic cod *aicda *isoforms. **A** Constitutive expression of Atlantic cod *Gm-aicda* (top panel) and *T-Gm-aicda* (middle panel) transcripts was compared to *ef1-α* (bottom panel) in 19 different tissues through RT-PCR. Purified Atlantic cod macrophages, isolated from different fish individuals, were stimulated with Poly(I:C) (pIC) and used as negative controls for *aicda* expression (MQ-pIC). The splenic sample of pIC-challenged fish, different from the tissue panel fish, at 24 HPI was used as positive controls for *Gm*-*aicda* expression. **B** *Gm-aicda* transcripts expression during Atlantic cod embryogenesis was analyzed in a mixture of fertilized eggs and cleavage-stage embryos that were collected after communal spawning and distributed into three tanks. Samples of ~ 180 eggs/embryos per tank were collected from 12 h post-fertilization (day 0) until the yolk-sac absorption stage (day 20) and flash frozen. Five micrograms of total RNA extracted from each sample was used for cDNA synthesis, and 2 µl of diluted cDNA (corresponding to 50 ng of initial RNA) was reverse transcribed using isoform-specific *Gm*-*aicda* primers. The expression of the Atlantic cod *elongation factor 1-α* (*ef1*-*α*) was studied as a house-keeping gene. **C** Analysis of Atlantic cod *aicda* transcript expression upon immune stimulation. The expression of *aicda* gene was analyzed 6 or 24 h post injection with either pIC or with Formalin-killed typical *A. salmonicida* (ASAL). *Gm-aicda* transcript expression was normalized to *rplp1* and *atps* expression, and the sample with the lowest normalized expression was used as calibrator. Data are represented as mean ± SEM (*n* = 10). Asterisks represent a significant difference between an immune-challenged group and the corresponding PBS-injected control group (*: *p* < 0.05; **: *p* < 0.01). The expression fold-change values are shown below the graphs

Previous studies on vertebrates have identified lymph node and spleen as the main *aicda* expressing tissues [23, 24, 51, 52, 56–58], while lower and variable levels of *aicda* expression was reported in thymus, pancreas, kidney, liver, and lung of mammals [23, 57, 58], in brain, intestine, kidney, liver, and lung of amphibians, and in intestine, fin, and posterior and anterior kidneys of fish [51, 52, 56]. Using RT-PCR, we detected the higher levels of *Gm-aicda* transcript expression in spleen, kidney, and gill, and lower levels in blood and heart (Fig. 2A). *T-Gm-aicda* transcript was expressed only at lower levels in immune-related tissues, notably spleen. Interestingly, only *T-Gm-aicda* transcript was detected in the male, but not female, reproductive tissue (Fig. 2). *Gm*-*aicda* is located on Linkage Group 11 which has been proposed to contain the majority of the Atlantic cod sex-locus [59]. In one scenario, its expression as *T-Gm-aicda* isoform, which lacks catalytic activity, might be due to proximity to sex-locus and lack of proper transcription factor(s) to safeguard the genome in male gonads. In another scenario, this expression is reminiscent of human AID and mammalian APOBEC4 expression in testis which might be a remnant of an ancient unknown role of AID [60–62]. No *Gm-aicda* transcript expression was detected in viral mimic [i.e., polyinosinic:polycytidylic acid (pIC)]-stimulated macrophages (Fig. 2A), nor during embryogenesis (Fig. 2B) as reported in zebrafish and the Iberian ribbed newt [56, 63, 64].

In mammals, *aicda* expression can be induced during B cell activation, either through interaction of peptide/MHCII complex and CD40 on B cells with T cell receptor and CD40L on CD4^+^ T helper (T_H_) cells, or through dual engagement of B cell receptor and TLRs on B cells with antigens such as lipopolysaccharide (LPS) [65–69]. Although both pathways lead to comparable *aicda* expression, the latter pathway takes place early in immune response when T_H_ cell assistance is not yet available [66]. Since loss of *cd4* and *mhc II* in Atlantic cod are highly suggestive of impaired canonical T_H_ cell function, we sought to investigate *Gm-aicda* expression in the early immune response [38, 39]. We observed approximately 3- and twofold higher expression of *Gm-aicda* in response to viral mimic (i.e., pIC) and bacterial antigen [formalin‐killed Aeromonas salmonicida (ASAL)], respectively, at 6 h post injection (Fig. 2C). In contrast, the splenic expression of *T-Gm-aicda* did not significantly change upon immune stimulation.

### The Atlantic cod AID is an extremely lethargic enzyme

**Fig. 3:**
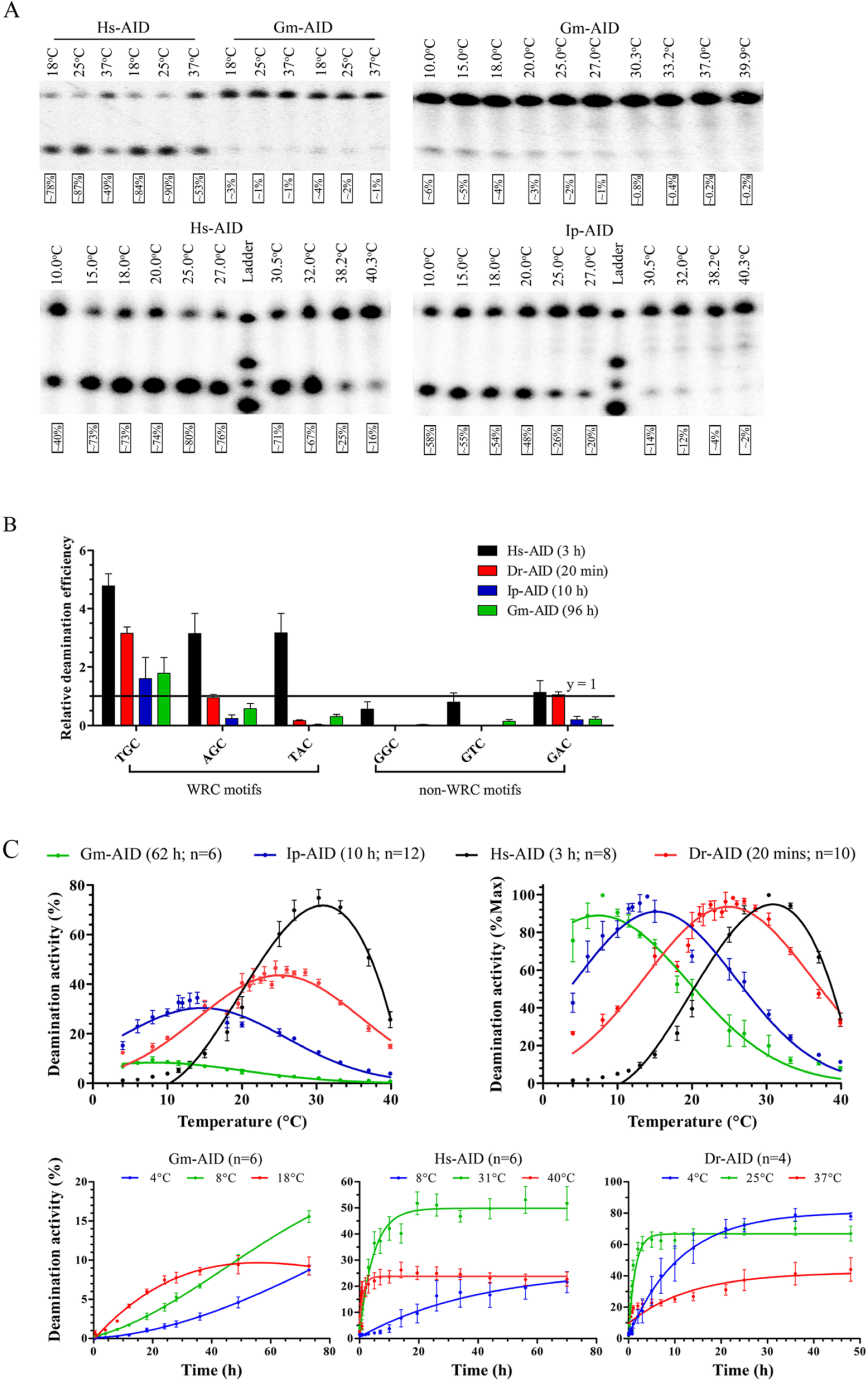
Biochemical characterization of the Atlantic cod AID’s enzymatic function.** A** Functional analysis of purified Atlantic cod AID. Gm-AID was expressed and purified alongside other AID homologs as a GST-fusion protein and tested for cytidine deamination activity using the standard alkaline cleavage assay. Deamination activity (% product generated) is presented below each lane. All experiments were done using ^32^P-labelled TGCbub7 substrate in duplicate. The upper left panel shows a typical alkaline cleavage gel wherein purified Gm-AID and Hs-AID were incubated with substrate at 18, 25, and 37 °C for 16 h showing barely detectible deamination activity for Gm-AID. The upper right panel shows a typical alkaline cleavage gel wherein 16-h prolonged incubation of purified Gm-AID with substrate revealed a preference for lower temperatures. The bottom panel shows a typical alkaline cleavage gel wherein the activity of Gm-AID was tested on ^32^P-labelled TGCbub7 substrate at various incubation temperature points alongside Hs-AID and Ip-AID of as controls. **B** The sequence specificity of Gm-AID was compared to that of other AID orthologs. AID was incubated with various substrates containing WRC (TGC, AGC, and TAC) or non-WRC (GGC, GTC, and GAC) motifs at their corresponding optimal temperature. In these experiments, three independent protein preparations were tested for each AID ortholog in duplicate (*n* = 6). Incubation time was selected based on catalytic robustness of each AID ortholog. Since the absolute activity level on each substrate varied among AID orthologs, relative deamination efficiency was used to enable comparison between AID orthologs. Relative deamination efficiency was calculated by dividing the activity on each substrate by that of the average activity for all 6 studied substrates. Data are represented as mean ± SEM. **C** Top panel shows the determination of the optimal temperature of Gm-AID compared to that of other AID orthologs at fine temperature increments (4 to 40 °C). Three to six independent protein preparations of each AID ortholog were tested in duplicates. Results are plotted as deamination activity percentage (left panel) and percentage of maximum deamination activity (right panel), revealing optimal temperature of 8, 14, 25, and 31 °C for Gm-AID, Ip-AID, Dr-AID, and Hs-AID, respectively. Data is represented as mean ± SEM. The bottom panel shows time course enzyme kinetics conducted at three temperature points (optimal, below, and above optimal) and corresponding optimal pH of each AID ortholog, in order to confirm the determination of the optimal temperature. Three independent preparations of Gm-AID (30 min to 73 h), Hs-AID (1 min to 70 h), and Dr-AID (30 s to 48 h) were tested in duplicate (*n* = 6). Data is represented as mean ± SEM

We expressed and purified Gm-AID and T-Gm-AID as N-terminally tagged GST fusion protein (Supplementary Fig. 3A) and tested their cytidine deaminase activity in the alkaline cleavage assay which is the standard assay used to measure the activity of purified AID/APOBECs (Supplementary Fig. 3B). This assay has previously been used in multiple studies to describe the biochemical properties of AID proteins [25, 70–72]. Using this method, we tested the activity of purified Gm-AID on a standard bubble substrate bearing the AID-favored WRC motif TGC, previously shown to be favored by AID of various species (Supplementary Fig. 3B) [71–73]. We observed extremely low amounts of deaminated product formation by Gm-AID compared to other human and bony fish AID orthologs from the zebrafish and channel catfish (Dr-AID, Ip-AID), and the observation of even this very low product levels of ~ 1–2% required prolonged enzyme:substrate incubations of up to 4 days (Fig. 3A). We then analyzed the substrate specificity of Gm-AID and found that Dr-AID and Ip-AID exhibited WRC preference, as did Gm-AID, favoring the two WRC motifs, TGC and AGC (Fig. 3B), consistent with previous findings [25, 72] and the high conservation in their substrate specificity loop sequences.

Since AID of at least one other bony fish, the zebrafish (*Danio rerio*), has been shown to function in epigenetic reprogramming since Dr-AID has the unique enzymatic ability to efficiently deaminate 5-methylated cytidine (5-mC) in CpG motifs [71], we wondered if Gm-AID may also play a similar non-immune role and assessed its activity on substrates containing 5-mC. We found that similar to a previous report [71], while all AID orthologs exhibited activity on both 5-mC and dC, they were less efficient in deaminating 5-mC, except for Dr-AID which showed robust activity on both substrates (Supplementary Fig. 3D).

We examined the optimal pH of AID homologs in buffer with effective pH ranging from 5.9 to 8.2. We found the optimal pH of about 7.3, 7.6, 7.9, and 8.1 for Hs-AID, Dr-AID, Ip-AID, and Gm-AID, respectively, as demonstrated [74]. We then examined the optimal temperature of AID orthologs. Consistent with previous studies [25, 72], we determined the optimal temperature of channel catfish AID (Ip-AID), zebrafish AID (Dr-AID), and human AID (Hs-AID) to be 14, 25, and 31 °C, respectively, while Gm-AID was most active at 4–12 °C with an optimal of 8 °C (Fig. 3A, C top panel), which corresponds precisely to its natural and optimal habitat temperature [75, 76]. To further confirm the cold adaptation of Gm-AID, time course enzyme kinetics were carried at optimal, higher, and lower than optimal temperatures. Gm-AID activity continued to increase at 8 °C even after 72 h, confirming 8 °C as the optimal temperature of this AID (Fig. 3C, bottom panel).

These experiments demonstrated that unlike the other AID orthologs that yield deaminated substrates in time frames in orders of several minutes to hours, Gm-AID required prolonged incubations of up to several days (24–96 h) before borderline detectible amounts of product could be observed, even with its optimal substrate sequence and at its optimal temperature (Fig. 3A, B). Since Gm-AID exhibited extremely weak cytidine deaminase activity, we verified that this is indeed bona fide cytidine deaminase catalytic activity by generating Gm-AID mutants lacking the catalytic glutamate (Gm-AID^E62G^ and Gm-AID^E62Q^, Supplementary Fig. 3C).

### Deamination catalysis by the Atlantic cod AID is several orders of magnitude less efficient than other orthologs

**Fig. 4:**
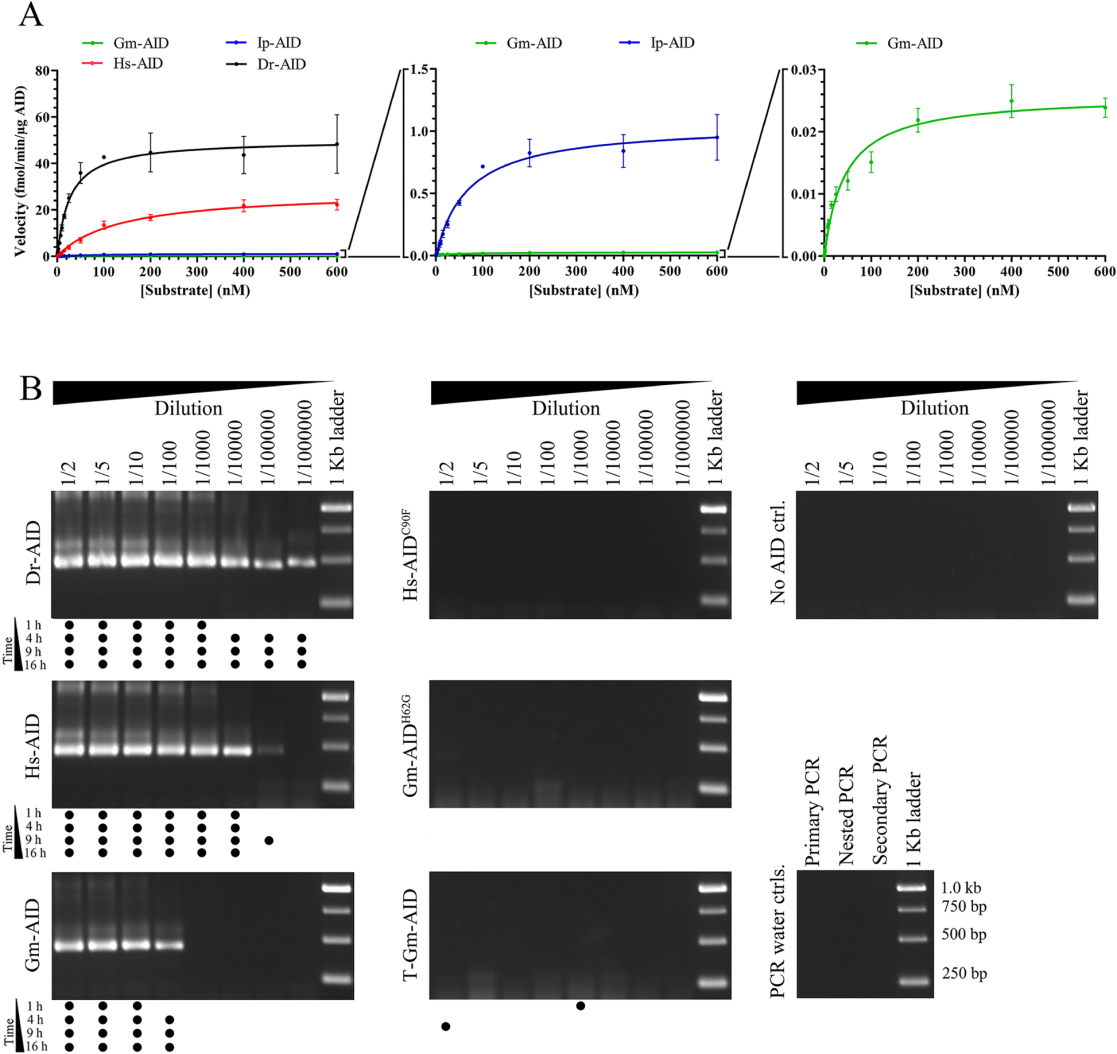
Comparison of the catalytic rate of Atlantic cod AID with other AID orthologs.** A** The catalytic rate of Gm-AID was compared to that of other AID orthologs through Michaelis–Menten kinetics determined by the alkaline cleavage assay. Three independent protein preparations of each AID ortholog were tested in duplicate. Data is represented as mean ± SEM (*n* = 6). **B** The relative catalytic activity of Gm-AID was confirmed through a PCR-based deamination assay using a single-stranded plasmid as the substrate. The presence of a PCR amplicon indicates AID-mediated deamination events. Through serial dilution analysis of the substrate incubated with AID which is used as the template for the deamination-specific PCR, it was apparent that Dr-AID is 10–100-fold more active than Hs-AID, while Gm-AID supported 100- and 10,000-fold less mutation levels than Hs-AID and Dr-AID, respectively. Four independent experiments were performed, and the presence of a PCR band in each experiment was recorded as a black dot below each lane in the representative gel

Having determined that Gm-AID is an extremely lethargic enzyme compared to other AID orthologs, we sought to precisely quantify this degree of difference in catalytic ability. We then quantified Gm-AID’s cytidine deaminase capability through two independent approaches (Fig. 4). First, we conducted standard Michaelis–Menten kinetics using the alkaline cleavage assay to compare the catalytic parameters (*K*_cat_, *K*_m_) of AID orthologs. Consistent with previous findings [41, 72, 77, 78], we observed that Dr-AID exhibited the highest catalytic efficiency (i.e., *K*_cat_/*K*_m_ ratio), ~ ninefold higher than Hs-AID, while the catalytic efficiency of Ip-AID was ~ 13-fold lower than Hs-AID. The catalytic efficiency of Gm-AID was ~ 3100, 350, and 25-fold lower than Dr-AID, Hs-AID, and Ip-AID, respectively as apparent by *K*_cat_ and *K*_cat_/*K*_m_ values (Fig. 4A, Table 1).

**Table 1:**
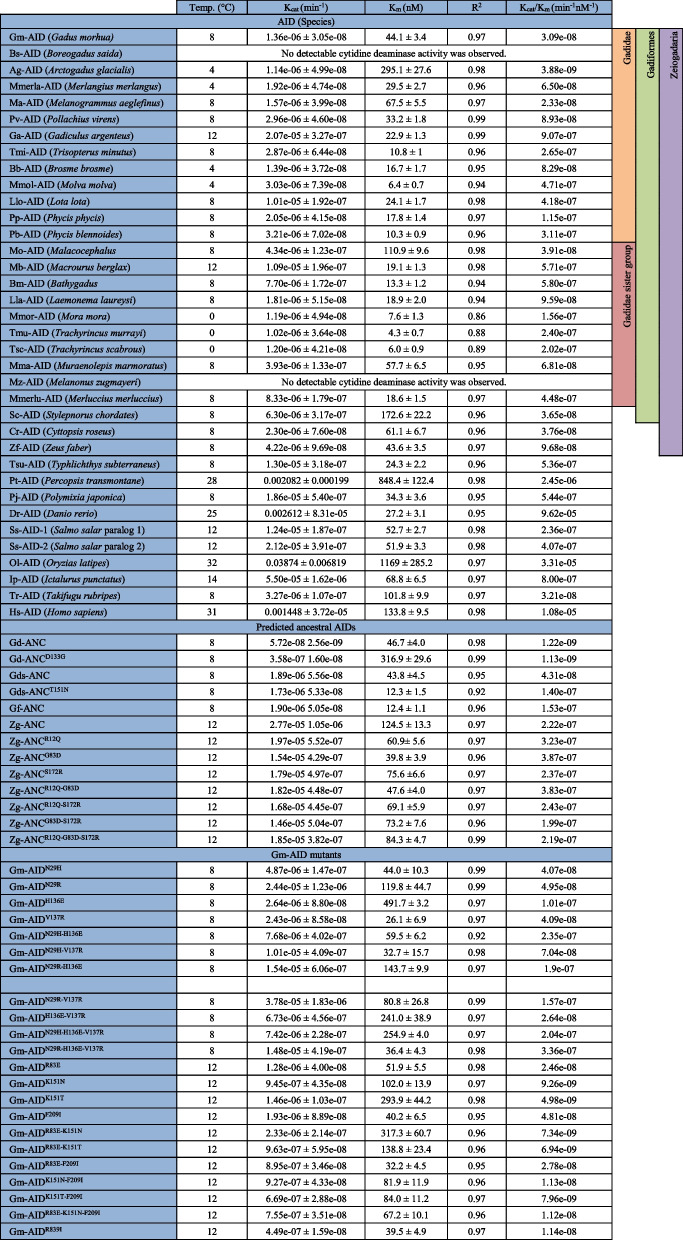
The enzymatic parameters measured for extant, ancestral, and mutant AID enzymes purified and examined in this study

As a second independent measurement, we used a PCR-based deamination assay which we have previously established and demonstrated to be a quantitatively accurate means of measuring differences in the catalytic activity of AID [25, 79]. This assay relies upon deamination-specific primers to quantify AID-mediated mutation levels on multi-kb-long DNA substrates [25, 79]. No cytidine deamination activity was detected for T-Gm-AID and the catalytically inactive mutants (Hs-AID^C90F^ and Gm-AID^E62G^), confirming the result of alkaline cleavage assay. We found Dr-AID to be 10–100-fold more active than Hs-AID, while Gm-AID supported 100- and 10,000-fold less mutation levels than Hs-AID and Dr-AID, respectively (Fig. 4B). In addition to being a second independent assay for deamination efficiency, this assay measures AID’s deamination on long ssDNA stretches of several hundred nucleotides in length that may form various secondary structures, thus exposing AID to more types of ssDNA substrates and sequence contexts than the defined bubble substrate used in the alkaline cleavage assay. The data from both enzyme assays indicate that even when tested in its most optimal conditions, Gm-AID is an extremely lethargic cytidine deaminase.

These data were obtained with bacterially expressed and purified GST-AID. Since this system has been used to demonstrate catalytic activity of many bony fish AID orthologs [71–73, 80], it is unlikely but formally possible that the extreme enzymatic lethargy of Gm-AID is due to mis-folding when expressed in bacteria. To examine this possibility, we expressed Gm-AID in a different expression system (293 T cells), along with Dr-AID as a positive control. In contrast to Dr-AID, in eukaryotic expression system, Gm-AID exhibits no detectable cytidine deamination activity (Supplementary Fig. 4). These data further confirm that Gm-AID is a functionally ineffective cytidine deaminase.

### Alternative interactions with ssDNA partially contribute to the reduced catalytic activity of Atlantic cod AID

**Fig. 5:**
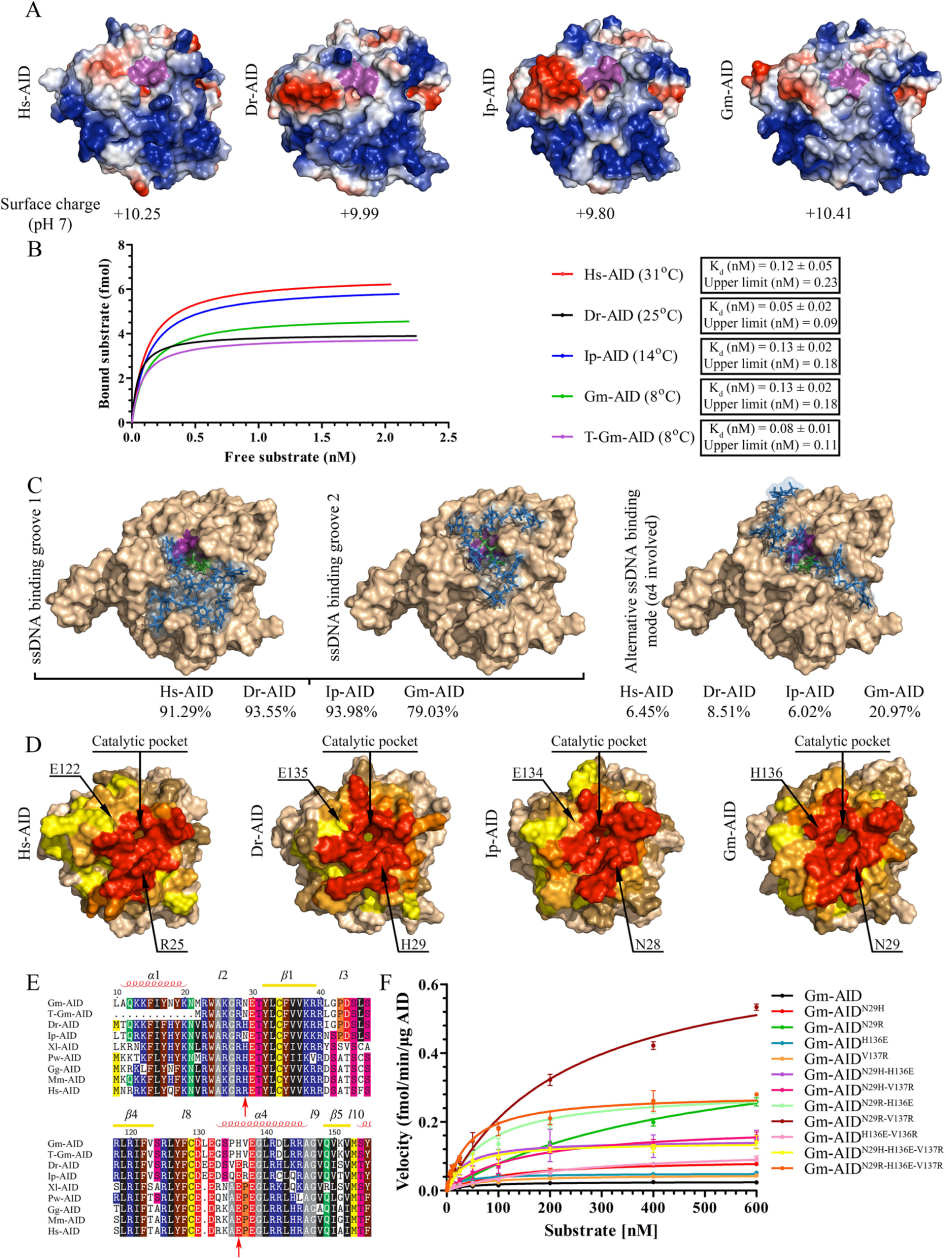
Structural modeling and mutagenesis to determine the basis of Atlantic cod AID lethargy.** A** Predicted surface topology of Gm-AID was compared to that of other AID orthologs. Positive, neutral, and negative residues are colored blue, white, and red, respectively. The putative catalytic pocket is colored in purple. Surface charge (at pH 7.00) is shown below each model. **B** The electrophoretic mobility shift assay (EMSA) was conducted to compare global ssDNA binding affinity of AID orthologs. Estimated *K*_d_ and upper limits show no significant difference among AID orthologs. **C** Docking of ssDNA on the surface of the Gm-AID model revealed the presence of the two main ssDNA binding groove 1 and 2 previously identified in Hs-AID, as well as alternative ssDNA binding mode which involved the α4 region. The contribution of different binding modes is shown for each AID ortholog. **D** Interactions between AID residues and ssDNA are shown as heatmaps. Amino acid residues interacting with substrate in 50–100%, 30–50%, 15–30%, 5–15%, 0–5%, and 0% of docking events are shown in red, dark orange, light orange, yellow, sand, and wheat colors, respectively. Shown with arrows are two potential amino acids that contribute to the increasing involvement of Gm-AID α4 and their counterparts in other AID orthologs. **E** Partial alignment of the AID orthologs surrounding Gm-AID^N29^, Gm-AID^H136^, and Gm-AID.^V137^ residues. The approximate secondary structure of α-helical (α), β-strand (β), and loop regions (l) are shown on top of the alignment. **F** The catalytic rate of Gm-AID mutants was compared to that of wildtype Gm-AID through Michaelis–Menten kinetics. Data are represented as mean ± SEM (*n* = 4)

We previously presented a functional and native structure for Hs-AID using a combined computational-biochemical method, which has been confirmed by its partial X-ray crystal structure [41, 81, 82]. Using the same methodology, we predicted the structure of Gm-AID by homology modeling based on various human APOBEC templates, as well as the aforementioned partial AID crystal structure. We found that its overall structure, catalytic pocket architecture, and surface charge were similar to those of other orthologs (Fig. 5A, supplementary data file 1). To examine Gm-AID binding to ssDNA, we conducted electrophoretic mobility shift assay (EMSA) and found that Gm-AID bind ssDNA with the same high nM range affinity previously measured in other orthologs (Fig. 5B) [25, 72].

EMSA provides a measure of global surface ssDNA binding but only a minor fraction of these interactions passes over the catalytic pocket and can be deaminated [41]. To evaluate catalytically viable ssDNA bindings, we performed docking simulations as used previously, which involves the docking of a simulated ssDNA polynucleotide onto the surface of AID [41, 81, 83]. Beside the previously defined putative ssDNA binding grooves, we noticed alternative ssDNA:AID interactions in which substrate was highly solvent exposed (Fig. 5C) [25, 41, 82, 84]. These alternative predicted ssDNA binding modes were due to the AID’s α4 region involvement in ssDNA binding which formed more interactions in Gm-AID relative to other orthologs (21, 6, 6, and 8% for Gm-AID, Ip-AID, Dr-AID, and Hs-AID, respectively; Supplementary Table 1).

Two potential residues responsible for this phenomenon, as suggested by docking, were Gm-AID^N29^ (equivalent of Hs-AID^R25^ and Dr-AID^H29^) and Gm-AID^H136^ (equivalent of a conserved E in all other orthologs; Hs-AID^E122^ and Dr-AID^E135^). The former is known to be important for efficient arching and positioning of dC into the catalytic pocket [41, 74, 81, 85, 86], and the latter appears to favor interactions with the − 1 position nucleotide upstream of dC (Supplementary Table 2). We generated several Gm-AID mutants and observed that Gm-AID^N29H^, Gm-AID^H136E^, and Gm-AID^N29H−H136E^ showed 5-, 3-, and 11-fold increase in catalytic efficiency (Fig. 5D–F, and Table 1). Therefore, these residues may partially contribute to, but do not fully explain the lethargic activity of Gm-AID.

### Lethargic enzymatic activity and extreme sub-zero cold adaptation of AID is a shared trait of other Gadinae species

**Fig. 6:**
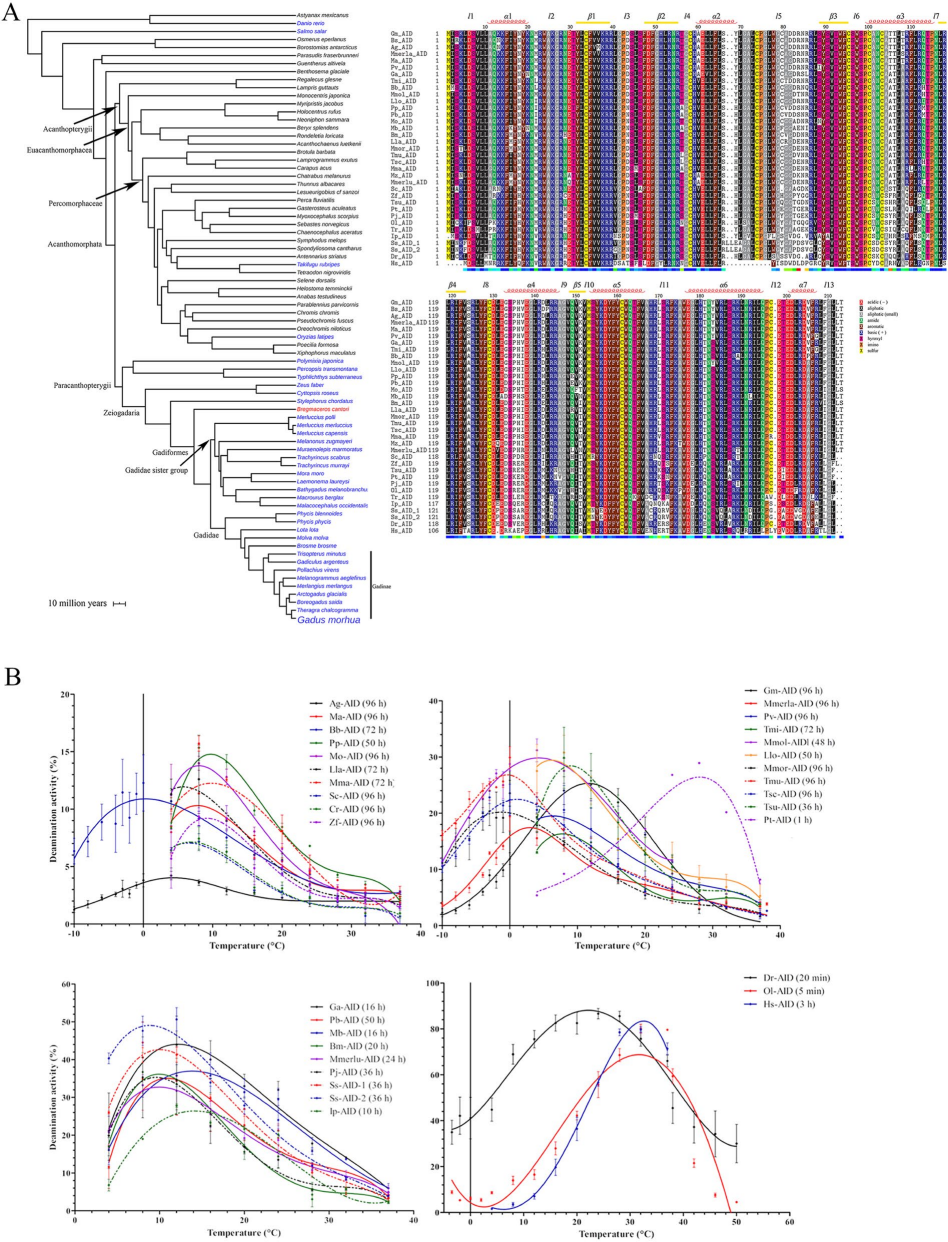
Biochemical characterization of 34 AID orthologs from species related closely to the Atlantic cod.** A** The left panel shows a species tree adapted from Malmstrom et al. [33]. AID proteins from species colored blue were synthesized in the lab to study their biochemical properties. Channel catfish and human AIDs were also purified and tested. We could not find any aicda gene in the genomic sequence of *B. cantori* (colored in red). The right panel shows the primary sequence of the AID enzymes that were expressed and purified. **B** The optimal temperature profiles of extant AID orthologs was assessed using our standard alkaline cleavage assay and bub7TGC substrate. Two to three independent protein preparations of each AID ortholog were tested in duplicates. Results are plotted as deamination activity percentage. The incubation duration, minimum, and maximum temperature limits were tailored to the activity level of each purified AID obtained in the preliminary results. For better representation, results were graphed based on the AIDs’ activity level. **A** through **D** show AIDs with low to high activity levels. Data is graphed as mean ± SEM (*n* = 4). Abbreviations: Gm: Gadus morhua; Bs: Boreogadus saida; Ag: Arctogadus glacialis; Memrla: Merlangius merlangus; Ma: Melanogrammus aeglefinus; Pv: Pollachius virens; Ga: Gadiculus argenteus; Tmi: Trisopterus minutus; Bb: Brosme brosme; Mmol: Molva molva; Llo: Lota lota; Pp: Phycis phycis; Pb: Phycis blennoides; Mo: Malacocephalus occidentalis; Mb: Macrourus berglax; Bm: Bathygadus melanobranchus; Lla: Laemonema laureysi; Mmor: Mora mora; Tmu: Trachyrincus murrayi; Tsc: T. scabrous; Mma: Muraenolepis marmoratus; Mz: Melanonus zugmayeri; Mmerlu: Merluccius merluccius; Sc: Stylepnorus chordates; Cr: Cyttopsis roseus; Zf: Zeus faber; Tsu: Typhlichthys subterraneus; Pt: Percopsis transmontane; Pj: Polymixia japonica; Ss: Salmo salar; Dr: Danio rerio; Ol: Oryzias latipes; Tr: Takifugu rubripes; Ip: Ictalurus punctatus; Hs: Homo sapiens

Having examined the AID of Atlantic cod as a Gadiformes representative species, we then explored whether this catalytic impairment of AID is a widespread trait within the Gadiformes lineage. We identified the gene for and expressed the AID protein of 34 extant Gadiformes and non-Gadiformes species (Fig. 6A, supplementary table 7, supplementary data file 2). Interestingly, we could not find a complete or partial *aicda* gene in the striped codlet (*Bregmaceros cantori*) genome, with the caveat that the currently available genome for this species has a low coverage. In our dataset, the striped codlet represents the most basal Gadiformes species and is characterized by the complete absence of *mhc I U*, *mhc II*, *cd4*, *cd8*, and *aicda* genes which are central to cell-mediated and humoral immune systems [33].

We then expressed and purified the 34 AID orthologs to examine their biochemical properties, as done for Atlantic cod AID. Of these, two orthologs of Bs-AID (*Boreogadus saida*, polar cod) and Mz-AID (*Melanonus zugmayeri*, arrowtail cod) did not exhibit cytidine deaminase activity (Fig. 6B). Through a mutagenesis approach, we determined that a single residue difference between Gm-AID and Bs-AID (L143P) was responsible for the absolute catalytic death of the latter (Supplementary Fig. 5). We discovered a cold adaptation of AID enzyme among species studied here which seemed to be governed by their habitat temperature as expected (Fig. 6B) [72, 77, 78, 87, 88].

Here we showed that some AID orthologs exhibit optimal cytidine deaminase activity in temperatures near or even below 0 °C (Fig. 6B, Table 1). Tsc-AID, Tmu-AID, and Mmor-AID demonstrated optimal temperature of 0 °C, remarkably maintaining more than 50% of their maximum catalytic activity at − 10 °C. *T. scarbus*, *T. murrayi*, and *M. mora* live in the deep-water (as low as 2000 m) and this might explain their lower optimal temperature (www.fishbase.se). We believe this finding to represent the coldest optimal temperature reported thus far for a vertebrate DNA/RNA-editing enzyme.

**Fig. 7:**
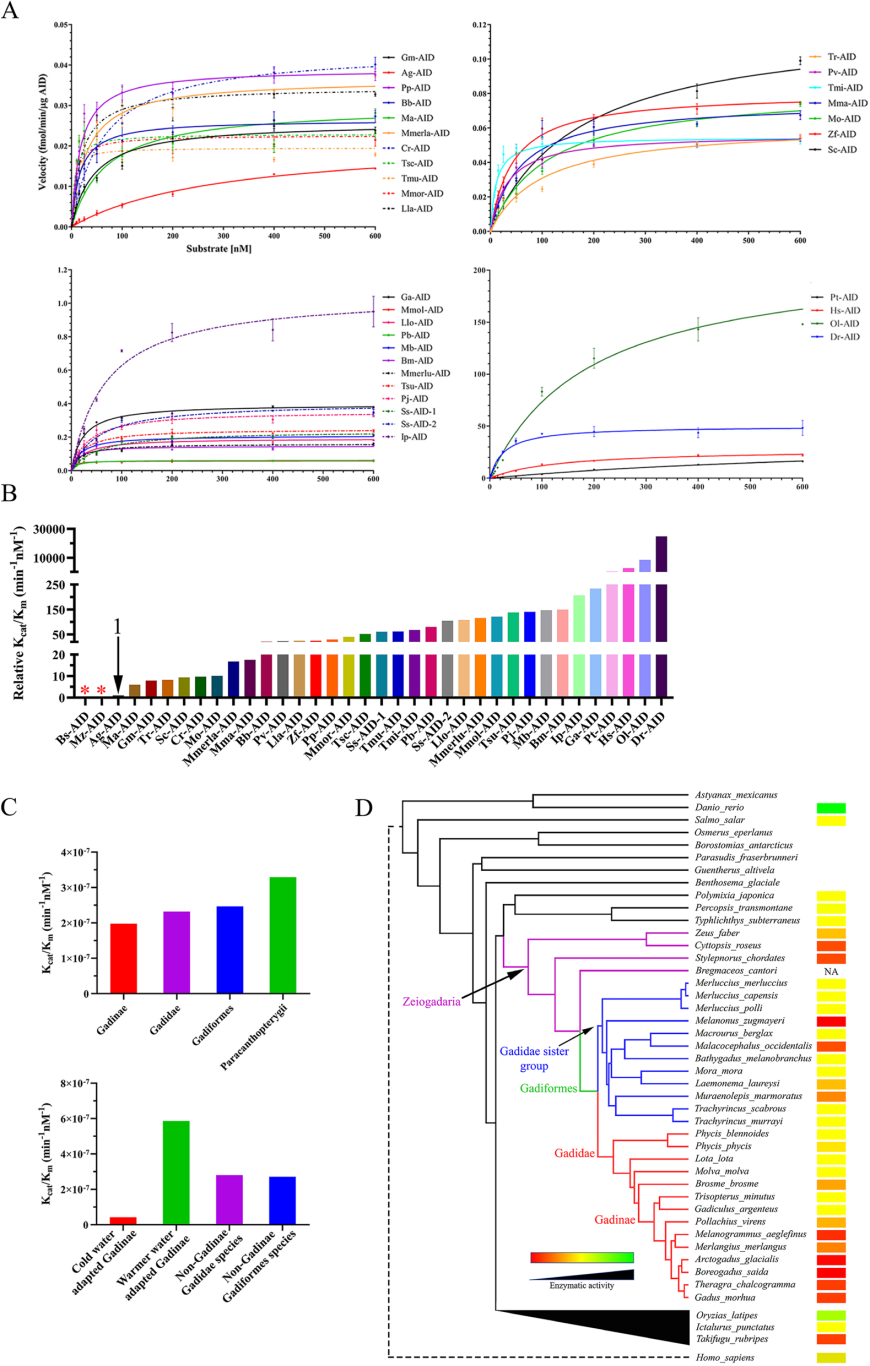
Comparison of catalytic efficiency of Gadiformes AIDs through Michaelis–Menten kinetics.** A** The catalytic rate of Gadiformes AIDs was compared to that of other AID orthologs through Michaelis–Menten kinetics. Thirty-six extant AID orthologs were expressed and purified as GST-tagged recombinant proteins. At least two independent protein preparations of each AID ortholog were incubated at their optimal pH and temperature with 0.03125–600 fmol range of TGCbub7 substrate. Each reaction was carried out in duplicate. For better visual representation, the data is graphed based on the AIDs’ activity level. **A** through **D** show AIDs with low to high activity levels. Data is represented as mean ± SEM (*n* ≥ 4). Abbreviations: Gm: *Gadus morhua*; Bs: *Boreogadus saida*; Ag: *Arctogadus glacialis*; Memrla: *Merlangius merlangus*; Ma: *Melanogrammus aeglefinus*; Pv: *Pollachius virens*; Ga: *Gadiculus argenteus*; Tmi: *Trisopterus minutus*; Bb: *Brosme brosme*; Mmol: *Molva molva*; Llo: *Lota lota*; Pp: *Phycis phycis*; Pb: *Phycis blennoides*; Mo: *Malacocephalus occidentalis*; Mb: *Macrourus berglax*; Bm: *Bathygadus melanobranchus*; Lla: *Laemonema laureysi*; Mmor: *Mora mora*; Tmu: *Trachyrincus murrayi*; Tsc: *T*. *scabrous*; Mma: *Muraenolepis marmoratus*; Mz: *Melanonus zugmayeri*; Mmerlu: *Merluccius merluccius*; Sc: *Stylepnorus chordates*; Cr: *Cyttopsis roseus*; Zf: *Zeus faber*; Tsu: *Typhlichthys subterraneus*; Pt: *Percopsis transmontane*; Pj: *Polymixia japonica*; Ss: *Salmo salar*; Dr: *Danio rerio*; Ol: *Oryzias latipes*; Tr: *Takifugu rubripes*; Ip: *Ictalurus punctatus*; Hs: *Homo sapiens*. **B** The relative catalytic efficiency (i.e., *K*_cat_/*K*_m_) of AID orthologs to the value of this parameter for Ag-AID (AID with lowest non-zero catalytic efficiency). We did not detect any cytidine deaminase activity for Bs-AID and Mz-AID (labelled with red asterisks). **C** The average catalytic efficiency of AIDs according to their phylogenetic classifications. **D** Schematic representation of catalytic efficiency of AID orthologs. Red to green color change indicates low (0: Bs-AID and Mz-AID) to high (9.62E − 05: Dr-AID) catalytic efficiency (Table 1). NA indicates species without *aicda* gene. The dashed line represents the manual addition of *Homo sapiens*. The black triangle represents the collapsed phylogenetic node

Having determined the optimal temperature for each of the 34 extant cod-related fish, we performed enzyme kinetics to determine the catalytic efficiency of each ortholog, defined as its *K*_cat_/*K*_m_ ratio (Fig. 7A, B, Table 1). We found that, on average, the catalytic efficiency of the Atlantic cod close relatives (i.e., Gadinae species) is slightly lower than the rest of Gadiformes lineage (1.99e − 07 *vs.* 2.47e − 07; Fig. 7C, Table 1). Within the Gadinae lineage, most of the fish species except for *G. argenteus* and *T*. *minutus* have adapted to cold water habitat (www.fishbase.se). Furthermore, the average catalytic efficiency of the cold water-adapted Gadinae AIDs was almost an order of magnitude lower than that of the warm water-adapted Gadinae species (4.25e − 08 *vs*. 5.86e − 07; Fig. 7D, Table 1, supplementary data file 3). These data indicate that the extremely weak AID catalytic efficiency is a common feature of the cold water-adapted Gadinae fish.

To examine the relationship between the catalytic efficiency of AID and its function in antibody diversification, we compared the average catalytic efficiency (*K*_cat_/*K*_m_ ratio) of AID of the cold water-adapted Gadinae which appear to lack SHM and antibody affinity maturation, to that of other bony fish in which AID-mediated SHM and antibody affinity maturation has been demonstrated, including the Atlantic salmon (Ss-AID), channel catfish (Ip-AID), human (Hs-AID), and zebrafish (Dr-AID) [13, 16, 18, 20, 89]. Indeed, we found that the latter group’s AID had an average catalytic efficiency (*K*_cat_/*K*_m_ ratio) of 2.70e − 05 which is a remarkable 3 orders of magnitude higher than that of the former group. Thus, the catalytic activity of AID in our dataset of > 30 orthologs corresponds to its function in mediating SHM and antibody affinity maturation.

### Ancestral reconstruction reveals the evolutionary trajectory of AID inactivation

The classical structure-guided mutagenesis approach (Fig. 5) yields insights into residues that can partially rescue the functionality of Gm-AID; however, this approach does not provide information on the actual nature of amino acid changes that during evolution have steered the AID of this branch of bony fish towards inactivation. In addition, the question remains as to at which evolutionary juncture relative to the unique loss of other central B cell activation genes (e.g.,* mhc II, cd4*) did the loss of AID’s enzymatic function occur; that is, was the former a prelude, or a consequence, of the latter? To gain insight into these questions, we utilized ancestral sequence reconstruction (ASR) to explore the evolutionary trajectory of AID enzyme within the Gadiformes lineage.

**Fig. 8:**
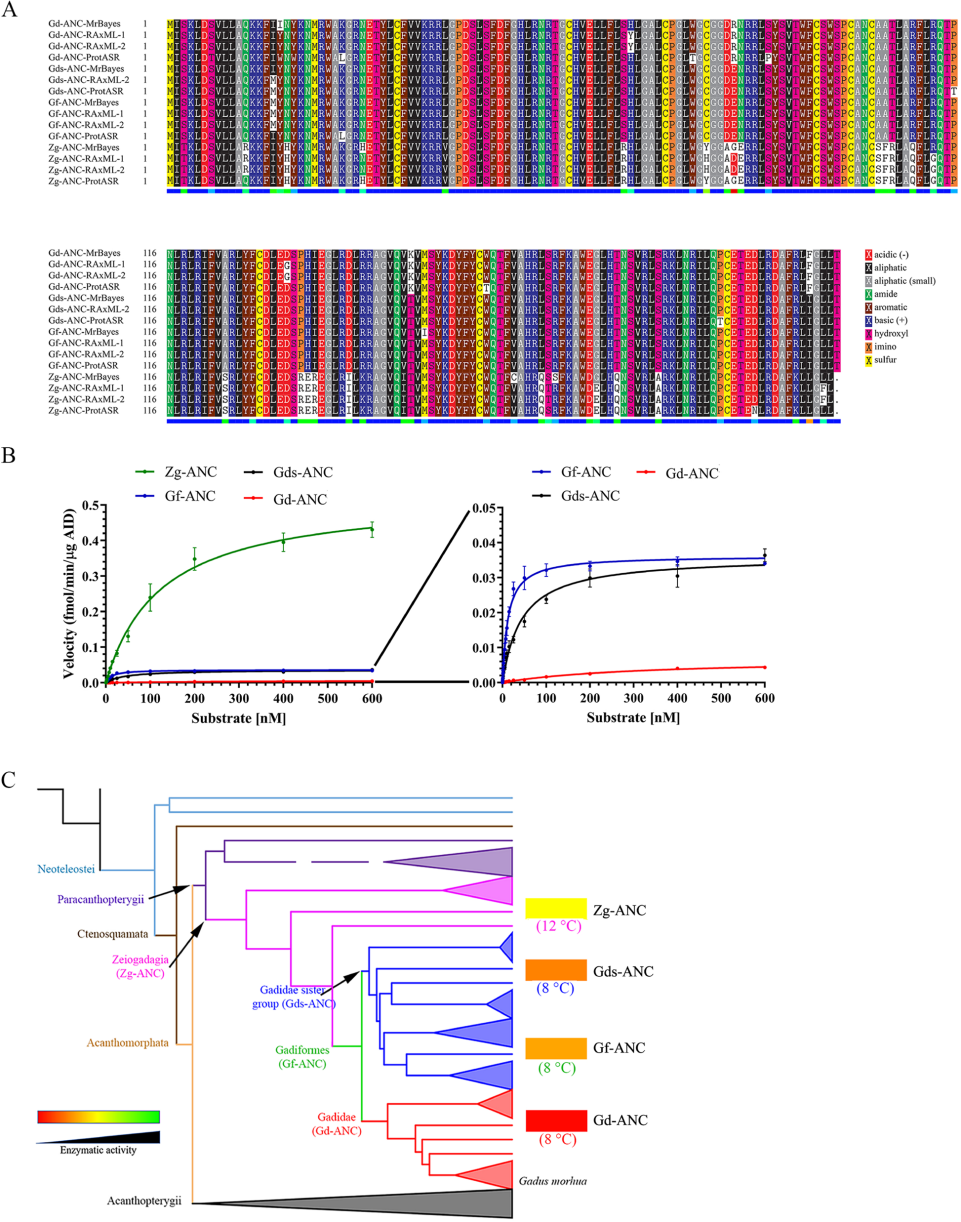
The catalytic efficiency of the resurrected ancestral AIDs.** A **Primary sequence alignment of predicted ancestral AID of four ancestral nodes: the common ancestor of Gadidae group (Gd-ANC); the common ancestor of Gadidae sister group (Gds-ANC); the common ancestor of Gadiformes lineage (Gf-ANC); and the common ancestor of Zeiogadaria group (Zg-ANC). Any amino acid positions with less than 0.8 probability were synthesized as mutants. Amino acids are colored based on their chemical properties as indicated in the bottom right corner legends. **B** The ancestral AID enzymes were expressed and purified and biochemically analyzed. For each resurrected ancestral AID, Michaelis–Menten kinetics was performed under its determined optimal pH and temperature conditions. **C** Schematic representation of the catalytic efficiency of predicted ancestral AIDs examined in this report. The same color scheme as Fig. 7 and as shown on the bottom right (green to red: highest to lowest catalytic efficiency) was used to assign a color code to the ancestral AIDs based on their catalytic efficiency

We based our ASR on the *aicda* gene sequence form 73 extant bony fish species and used the Pacific lamprey (*Lampetra tridentata*) CDA1 (AID-like protein) as the outgroup (Supplementary Fig. 6, supplementary data file 2, supplementary table 7). The amino acid alignment was guided by the predicted 3D structure of Gm-AID. We predicted the ancestral sequences using a previously published Gadiformes species tree [33], and three different methods implemented in MrBayes [90–94], RAxML [95], and ProtASR [96, 97] packages (supplementary data file 4). We thus predicted the ancestral *aicda* gene sequence of Gadidae (Gd-ANC), its sister group (Gds-ANC), Gadiformes (Gf-ANC), and Zeiogadaria (Zg-ANC). Previous studies have shown that ASR results obtained from Bayesian inference, especially the hierarchical Bayes approach (e.g., implemented in MrBayes package), outperform the results of other methods [98, 99]. Therefore, the consensus protein sequence for each ancestral node was predicted with a higher emphasis on MrBayes results (Fig. 8A). Variants of the ancestral AIDs were also generated if an amino acid position was predicted ambiguously (i.e., positions with a statistical uncertainty of 0.2 or higher; Supplementary Fig. 6) [100].

These predicted ancestral AID enzymes were then expressed, purified, and biochemically analyzed. We found that the optimal temperature of AID was reduced from 12 to 8 °C in Gadiformes common ancestor (Gf-ANC) (Supplementary Fig. 7). We found that the juncture at which AID’s catalytic activity was reduced by 99.2% to be the transition between the ancestors of the Gadiformes family (Gf-ANC) and the immediate Gadidae branch (Gd-ANC) that includes the Atlantic cod (1.53e − 07 vs. 1.22e − 09; Fig. 8B and Table 1).

To gain insight into the amino acid changes that drove the evolution of Gadidae AID to weaker enzyme activity, we compared the amino acid changes in the predicted ancestral AIDs. The ~ 35-fold reduction in the *K*_cat_ of the Gd-ANC compared with Gds-ANC was the result of four amino acid differences (i.e., I17Y, R83E, K151T, and F209I in Gd-ANC *vs*. Gds-ANC; Supplementary Fig. 6, Fig. 8A). Of these four differences, three (except for position 17) are shared between Gd-ANC and Gm-AID. We mutated the other three positions in Gm-AID into the corresponding amino acids in the Gds-ANC. Among these mutants, only Gm-AID^F209I^ showed higher catalytic efficiency compared with Gm-AID (4.81e − 08 *vs.* 3.09e − 08, Table 1), suggesting that the change of F209 to I may have contributed to the reduced catalytic efficiency of Gm-AID. This change only requires a T to A mutation in the first codon position (TTT and TTC encode F, and ATT and ATC encode I). However, this slight improvement in catalytic efficiency is far less than the 35-fold difference observed in the catalytic efficiency of Gds-ANC compared with Gd-ANC, suggesting the presence of epistatic mutations within Gm-AID which prevented the positive effect of causative mutation(s) to be observed. Also, comparison of the amino acid sequence of Gf-ANC with Gds-ANC and Gd-ANC revealed that residue 16 is uniquely different in the Gf-ANC which contains a methionine (Fig. 8A). Since Gds-ANC which contains an isoleucine in position 16 (similar to Gd-ANC) exhibited the same *K*_cat_ as Gf-ANC (1.89E − 06 *vs*. 1.90E − 06, respectively) but the same *K*_m_ as Gd-ANC (43.82 *vs*. 44.05, respectively), we concluded that position 16 could be responsible for the 376% increase in the *K*_m_ of the Gd-ANC compared with Gf-ANC.

### Gadidae Ig genes are co-evolved with their nearly inactivated AID

Previous studies have revealed a co-evolution between AID substrate specificity, i.e., WRC motifs, and the sequence of *Ig* variable (V) genes of mammals, birds, amphibians, and bony and cartilaginous fish [87, 101–106]. An accumulation of overlapping AID (especially AGCT) and Polη (WA) hotspots in the CDR1 and 2 compared to the framework (FRs) regions was observed in human IGHV3-23*01 region [106]. Also, among serine codons, a clear preference for AGY (WRC) over TCN (non-WRC) was observed in *IgV* CDRs *vs.* FRs [101–103, 105]. Moreover, the distribution of WGCW motifs, which contain AID hotspots on both strands, has been suggested as a key evolutionary feature of *IgV*_*H*_ genes in human [107] that attracts AID to the *IgV* regions [106, 108–110].

**Fig. 9:**
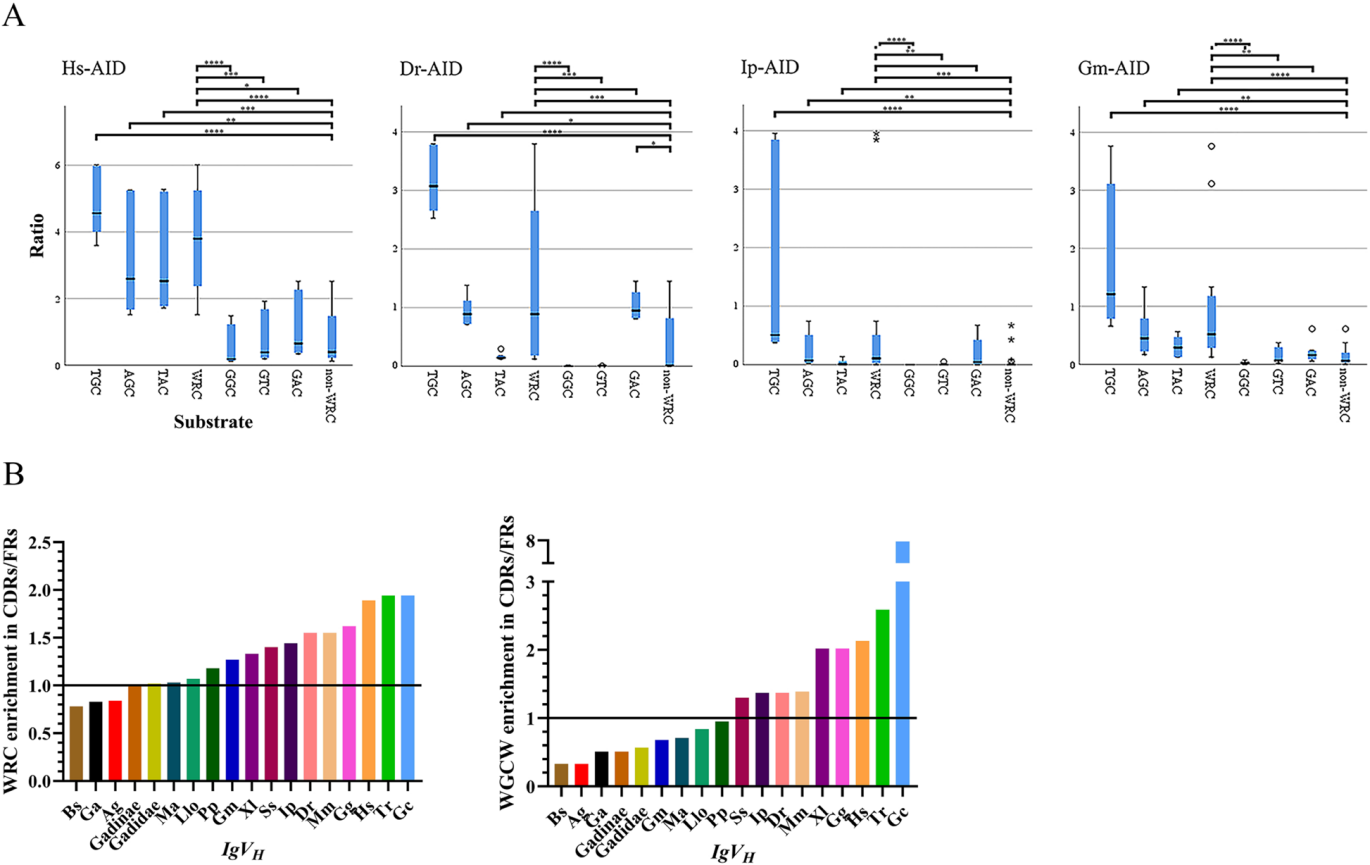
Co-evolution of the catalytic efficiency of AID with WRC enrichment in the Ig variable gene sequences in Gadidae species.** A** Substrate sequence preference of Gm-AID compared to that of other AID orthologs as determined by the statistical analyses of the difference observed between substrates of varying sequences’ relative deamination efficiency by various AID orthologs in the alkaline cleavage assay such as shown in Fig. 3B. The statistical difference between AID orthologs were calculated using the independent samples Rruskal-Wallis test. The null hypothesis was considered as the distribution is the same between each pair of samples (*n* = 6; *: *p* < 0.05; **: *p* < 0.01; ***: *p* < 0.005; ****: *p* < 0.001). Abbreviations: Gm-AID: Atlantic cod AID; Dr-AID: zebrafish AID; Ip-AID: channel catfish AID; Hs-AID: human AID. To assess the co-evolution of AID activity with *IgV*_*H*_ sequences in Gadidae species, enrichment of **B** WRC motifs (AID hotspots on both strands) and **C** WGCW motifs (overlapping AID hotspots on two strands) in CDRs of Gadidae species were compared to that of several other vertebrate species. The number of WRC/GYW or WGCW motifs were counted and normalized to the number of analyzed nucleotides. For each species, the average normalized value for CDR1 and CDR2 was divided by the average value of this number for FR1, 2, and 3. Abbreviations: Bs: *B. saida*; Ga: *G. argenteus*; Ag: *A. glacialis*; Ma: *M. aeglefinus*; Llo: *L. lota*; Pp: *P. phycis*; Gm: *G. morhua*; Dr: *D. rerio*; Ss: *S. salar*; Ip: *I. punctatus*; Tr: *T. rubripes*; Gc: *G. cirratum*; Xl: *X. laevis*; Gg: *G. gallus*; Mm: *M. musculus*; Hs: *H. sapiens*

Having determined that Gm-AID exhibits similar WRC specificity to other AID orthologs (Figs. 3B and 9A), we reasoned that if the functional impairment of AID is a purposeful event in the evolution of Gadidae species, there ought to have been a lower degree of evolutionary pressure to maintain enrichment of WRC motifs in the CDR regions of their *IgV* genes. We annotated the *IgH* loci in the Atlantic cod genome, which was then used to extract and annotate the *IgV*_*H*_ of Gadidae species. We also included *IgV*_*H*_ of Japanese puffer fish (*Tr-IgV*_*H*_), nurse shark (*Gc-IgV*_*H*_), human (*Hs-IgV*_*H*_), mouse (*Mm-IgV*_*H*_), chicken (*Gg-IgV*_*H*_*)*, South African toad (*Xl-IgV*_*H*_), catfish (*Ip-IgV*_*H*_), salmon *(Ss-IgV*_*H*_), and zebrafish (*Dr-IgV*_*H*_). The motif enrichment was calculated as the ratio of the average normalized index (i.e., the number of WRC/GYW or WGCW motifs divided by the numbers of analyzed nucleotide) in CDRs *vs*. FRs. We excluded CDR3 since the VDJ recombination is responsible for forming CDR3.

We found that, compared with other vertebrate species, the CDRs of Gadidae species have low/no AID hotspot (i.e., WRC and WGCW motifs) enrichment (Fig. 9B; Supplementary Tables 3 and 4) despite comparable abundance of AID targeting motif in their entire *IgV*_*H*_ fragments and higher GC content of their coding sequences (Supplementary Table 5). Within the Gadidae species, Gadinae fish revealed more drastic lack of AID hotspots in their CDRs. The CDRs of polar cod (*Bs*-*IgV*_*H*_), arctic cod (*Ag*-*IgV*_*H*_), and silvery pout (*Ga*-*IgV*_*H*_) contain less WRC/GYW motif than the FRs (enrichment of 0.78, 0.84, and 0.83, respectively). On average, Gadinae and Gadidae *IgV*_*H*_ regions had equal distribution of WRC/GYW motifs in their CDRs and FRs, while other species studied here exhibited WRC/GYW enrichment in their CDRs over the FR regions. When we compared the WGCW distribution in *IgV*_*H*_ regions, we noticed that all and only Gadidae species had more WGCW motifs in their FRs, with *Bs*-*IgV*_*H*_ and *Ag*-*IgV*_*H*_ exhibiting the least WGCW motif enrichment in the CDR regions, indeed being threefold less frequent in the CDRs relative to their FRs. This correlates well with Bs-AID being catalytically inactive. On average, the Gadinae and Gadidae species showed twofold enrichment of WGCW in their FRs, despite having a comparable abundance of AID targeting motifs in their entire *IgV*_*H*_ region and a higher GC content in their coding sequences (Supplementary Table 5). Thus, among the *IgV*_*H*_ CDR regions of all species examined here, that of the Gadidae species, especially the latest-emerged Gadinae that includes the Atlantic cod, exhibited a specific lack of AID hotspot motif enrichment. This correlates well with their near inactive AID enzyme.

## Discussion

**Fig. 10.**
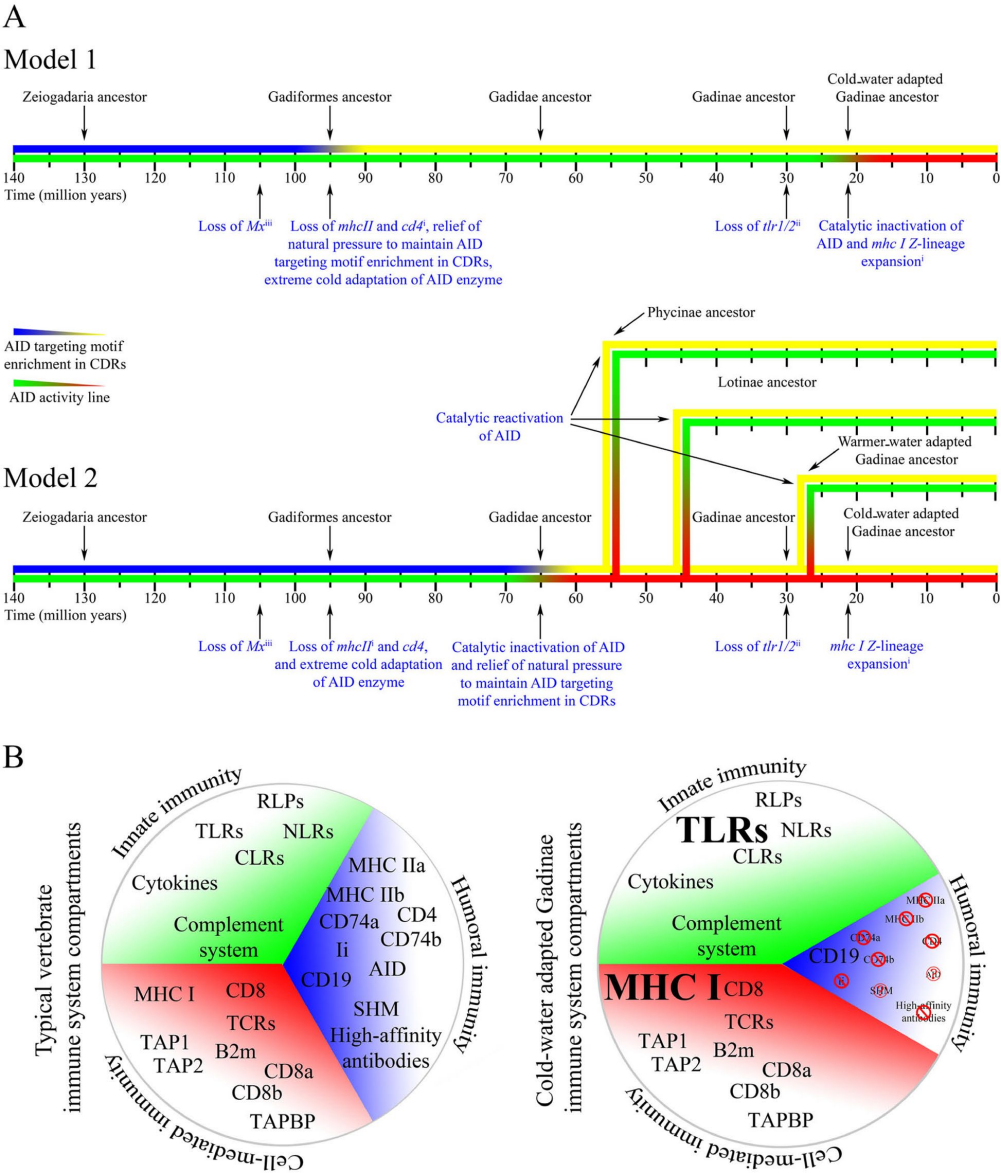
Proposed models of the evolutionary trajectory of the immune system in cold water-adapted Gadinae species. **A** Two possible scenarios are proposed to connect the relative timing and connection between the evolutionary events of the loss of many adaptive immune genes (e.g., *mhc II* and *cd4*), relief of natural selection to AID hotspot enrichment in Ig variable genes, and AID catalytic inactivation. **B** Cold water-adapted Gadinae species are unusual among bony fish and vertebrates in that they are missing many genes that are essential for a robust antibody response and contain an inactivated AID enzyme. On the other hand, they exhibit an expansion of other genes involved in cell-mediated and innate immunity

Here we demonstrated the catalytic inactivation of AID in cold water-adapted Gadinae species. Given that these fish have undergone a dramatic loss of many other key adaptive immune genes involved in the T cell–B cell interaction and activation [39], we propose two models to explain the timing of AID inactivation within the Gadiformes lineage (Fig. 10A). In model 1, the loss of pathways required for T cell-dependent B cell activation hindered antibody affinity maturation, resulting in relieving the natural selection to maintain AID targeting motif enrichment within Ig variable region CDRs. In cold water-adapted Gadinae, the ambient temperature of the species became closer to the optimal temperature of the ancestral AID, increasing the risk of AID-mediated collateral genome damage, while the lower load of pathogens in cold water settings relived the selection to maintain an active AID, resulting in AID functional impairment. The extreme cold adaptation of the Gadiformes ancestor AID (Gf-ANC) might be the evolutionary consequence of previously proposed adaptation of Gadiformes ancestor to deep-water settings [111] where the ambient temperature was lower and contained a lower microbial abundance [112]. The asynchronous cold adaptation (occurring first) and functional impairment (occurring later) in ancestral AIDs suggest that the functional impairment of AID is a purposeful event and not a by-product of enzyme cold adaptation. In the second model, the natural selection to maintain the AID’s targeting motif enrichment in CDRs was not relived until inactivation of AID in the Gadidae ancestor due to an unknown event. Later, AID was reactivated in Gadidae species living in warmer water environment due to higher load of pathogens. This model is less likely due to requiring both inactivation and re-activation events. Future studies of additional Gadidae species would assist in favoring one model over the other.

There are also two possible scenarios regarding the preservation of *aicda* gene in the Gadidae genomes while its functionality has been impaired. In the first scenario, AID might have evolved to take on another role in these species such as epigenetic reprogramming enzyme through 5mC demethylation as has been suggested in other bony fish [71]. However, our finding that the already lethargic activity of Gm-AID is weaker on substrates containing 5mC, as well as the fact that Gm-AID is not expressed during embryogenesis, argue against this model. Nevertheless, this still formally leaves open the possibility that Gm-AID might play an unknown biological role in these species that does not depend on its catalytic function. In the second scenario which is more supported by our evidence, the *aicda* gene within the Gadidae family is currently at a point in evolution that is headed towards elimination from the genome, like the Anglerfish and the Pipefish [30, 31]. Three lines of evidence strongly support this evolutionary trajectory by providing direct examples of other species along this evolutionary path: first, that in two closely related species, the polar “arctic” cod (*Boreogadus saida*) and Arrowtail (*Melanonus zugmayeri*) the catalytic activity of AID has been completely abolished, and further to this, that the catalytic death of the polar cod AID (Bs-AID) as opposed to the extreme catalytic lethargy of the Atlantic cod AID (Gm-AID) is mediated by a single-nucleotide difference in the gene (L143P, Supplementary Fig. 5) meaning that Gm-AID is a single nucleotide in its coding sequence away from total abolishment of any catalytic activity; second, that examples of *aicda* gene loss have been reported in other bony fish; and third, that the catalytic inactivation of AID in the Gadiformes clade of bony fish correlates precisely with the loss of WRC enrichment in the IgV regions, with the lowest degree of WRC motifs in the polar cod which, as mentioned above, has an absolutely inactivated AID, and the second lowest in species with AID that has the most lethargic (nearly inactive) catalytic activity. Altogether, these events illuminate the clear evolutionary path which will lead to the future loss of the *aicda* gene in Gadinae, assuming that environmental conditions also continue on trajectory.

We report the catalytic efficiency of fugu AID (Tr-AID) to be comparable to Gm-AID, while its AID is capable of inducing class switch recombination [77] and its CDRs are heavily enriched with AID hotspots. Although we previously showed that the initial velocity of nurse shark AID is 5 times lower than Hs-AID [25], we observed the highest level of WGCW enrichment in its CDRs despite the lowest occurrence of these motifs in the entire *IgV*_*H*_ regions compared with other vertebrate species, in line with the previous evidence of antibody affinity maturation in this species [12]. In these species, which are warm(er) environment adapted, the high degree of AID motif enrichment in CDRs could be a compensatory mechanism for the low catalytic efficiency of their AIDs. In contrast, the cold water-adapted Gadinae species have an extremely weak or inactive AID enzyme and their CDRs lack AID’s targeting motif enrichment, reflecting their lack of antibody affinity maturation.

In humans and mice, and presumably other mammals, AID deficiency leads to the hyper-IgM syndrome type II characterized by a lack of affinity-matured and isotype-switched antibodies and immunodeficiency. The recent reports that two other fish species, the anglerfish and pipefish, lack the aicda gene and RAG (in some species), brought to light a new level of flexibility in evolution of adaptive and innate immunity and challenging the long-standing paradigm that these enzymes are essential for functional immunity in all vertebrates [31]. These species presumably have downmodulated their adaptive immunity as a measure of co-evolution with their unique reproductive behaviors in order to avoid tissue rejection. Our work provides a second example of this type of adaptive immune loss in a vertebrate species, but in this case, due to the catalytic inactivation of an expressed AID protein, and not associated with unusual reproductive behavior, but rather with an unusually expanded innate and cell-based immunity and adaptation to cold temperatures.

We have resurrected the ancestors of an enzyme key to immunity and reported an instance of natural AID inactivation in vertebrates, providing yet more evidence that in the right environmental conditions, alternative immune strategies where the cellular/innate immune systems expand to compensate for the shrinkage of the adaptive humoral response can be successful in protecting the host (Fig. 10B). In the case of the Gadiformes, the aicda gene is maintained despite the enzymatic function loss, leading us to hypothesize that this group of bony fish has undergone a very recent AID function loss, wherein the gene has not yet been lost but is on trajectory for loss. The Gadiformes with functionally impaired AID are not only healthy but they are among the most thriving species within their habitats. This brings to light a previously unappreciated type of plasticity in the definition of what a successful immune system requires for protection in a species’ habitat. From an application point of view, our data are also informative for the design of immunomodulatory agents, given the importance of the Gadiformes lineage in aquaculture.

## Conclusions

The function of AID in driving secondary antibody diversification is conserved across jawed vertebrates with the recently reported exceptions of the anglerfish and pipefish, two species that have lost the *aicda* gene encoding AID. This is presumed to be due to unusual reproductive behavior including sexual parasitism and male pregnancy, requiring a downregulation of immunity to avoid tissue rejection. Here, we report a new scenario of AID loss in a clade of bony fish that is not associated with sexual behavior but rather with cold temperature adaptation. In this case, the gene encoding AID is maintained but the protein’s catalytic function has been largely or completely lost. These species have also reorganized and expanded components of their innate and cell-based immunity, which appears to compensate for the loss of the classical AID-driven antibody diversification in adaptive immunity.

Through ancestral reconstruction and the resurrection of extinct ancestor AID enzymes for functional enzymology studies, we delineated the timeline and amino acid changes leading to this catalytic inactivation. This work represents an application of ancestral reconstruction followed by functional enzymology to study the evolution of an enzyme involved in immunity and cancer. This represents an example of AID catalytic activity loss in a family of vertebrate species that are not only healthy but among the most thriving species in their habitat. Our findings provide yet another example of how the “classic” vertebrate immune system is considerably more plastic than previously thought in the evolutionary divisions between adaptive and innate immunity.

## Methods

### Gene locus structure and synteny analysis of aicda

Locus structure of Atlantic cod *aicda* was compared to that of zebrafish (*Danio rerio*), channel catfish (*Ictalurus punctatus*), African clawed frog (*Xenopus laevis*), tropical clawed frog (*Xenopus tropicalis*), chicken (*Gallus gallus*), mouse (*Mus musculus*), and human (*Homo spiens*). Sequences were retrieved from NCBI and Ensembl databases.

The *aicda* gene synteny was assessed both manually and using the synteny database. The 1-Mb regions containing *aicda* locus in Atlantic cod, three-spined stickleback (*Gasterosteus aculeatus*), Japanese pufferfish (*Takifugu rubripes*), zebrafish, spotted gar (*Lepisosteus oculatus*), coelacanth (*Latimeria chalumnae*), green anole (*Anolis carolinensis*), chicken, mouse, and human were derived using the assemblies from the Ensembl database. In the case of the tropical clawed frog, the genomic region was retrieved from Xenbase database (http://www.xenbase.org/entry/). The annotated genes within this 1-Mb region were then manually inspected to obtain Fig. 1B. Additionally, the synteny database was used (http://syntenydb.uoregon.edu/synteny_db/) [113]. The chromosomal location of zebrafish AID (*Dr-aicda*) was compared to that of Japanese pufferfish, three-spined stickleback, spotted gar, mouse, and human. Also, *Hs-aicda* synteny was compared to that of the mouse, spotted gar, and the tropical clawed frog.

### Identification and characterization of Gm-aicda transcript(s)

Samples used in this experiment were collected for a previously published study [114, 115]. Fish were sacrificed by submersion in an anesthetic bath containing tricaine methanesulfonate (MS-222, 400 mg L-1, Syndel Laboratories, Canada). Unless otherwise mentioned, all protocols were conducted according to the manufacturers’ instructions. RNA extraction and clean-up were conducted using TRIzol reagent (Invitrogen, Carlsdad, CA), followed by DNase-I treatment and RNeasy MinElute Clean-up Kit (Qiagen, Mississauga, ON) [114].

cDNA synthesis was performed using 1 µg of purified total RNA and SuperScript III Reverse Transcriptase (RT) (200 U, Invitrogen, Thermo Fisher Scientific, Waltham, MA). In a 25-µl PCR reaction, 1 µl of diluted cDNA (equivalent to ~ 100 ng of initial total RNA) was amplified using TopTaq DNA polymerase (QIAGEN) and 0.2 µM of Atlantic cod AID (*Gm-aicda*) specific primers. These primers were designed based on the *aicda* coding sequence identified in Atlantic cod genome project (Supplementary Table 6). No-template and no-RT reactions were also included in PCR-based experiments. Touchdown PCR cycling conditions were an initial denaturation step for 3 min at 94 °C followed by 35 cycles of [30 s at 94 °C; 30 s at 65 °C → 54.5 °C, decreasing 0.3 °C per cycle; and 1 min at 72 °C] and 10 min at 72 °C. PCR products were gel extracted (MinElute Gel Extraction Kit, Qiagen) and TA-cloned into pCR 2.1-TOPO TA vector (TOPO TA Cloning Kit, Invitrogen, USA) and transformed into *E. coli* Top10 cells. Purified TA-cloned plasmid preparations (Presto Mini Plasmid Kit, Geneaid, Thermo Fisher Scientific) were Sanger sequenced (Macrogen, South Korea).

To obtain full-length mRNA, rapid amplification of cDNA ends (RACE) PCR was performed. Sequencing results from the previous step were used to design gene-specific RACE-PCR primers (Supplementary Table 6). Splenic RNA extracted from pIC-stimulated fish (24HPI) was used, and RACE-PCR was carried out using SMARTer RACE cDNA Amplification kit (Clontech, Takara Bio Company, Mountain View, CA). To obtain 3′/5′-RACE-Ready cDNA, 1 µg of cleaned total RNA was reverse transcribed. cDNA was 3 × diluted in Tricine-EDTA buffer. For 3′-RACE and 5′-RACE, 2.5 µl of diluted 3′/5′-RACE-Ready cDNA (equivalent to ~ 75 ng of initial RNA) was amplified using gene-specific primers and Universal Primer A mix. PCR products were gel extracted using Qiagen gel extraction kit. For nested 3′-RACE or 5′-RACE, 5 µl of 50 × diluted primary PCR product (~ 400 pg/µl) was re-amplified using the same conditions, except Nested Universal Primer A mix, and nested primers were used. A PCR consisting of 25 cycles of [30 s at 94 °C; 30 s at 68 °C; 3 min at 72 °C] was conducted. PCR bands were gel extracted and sequenced as described above. Sequencing data were assembled and analyzed using Lasergene 7 MegAlign software (DNASTAR, Inc., Madison, WI).

To confirm the presence of two *Gm-aicda* transcripts, isoform-specific primers (ISPs) were designed (Supplementary Table 6) and the splenic RNA of pIC-stimulated fish sampled at 24 HPI were used. One microgram of clean total RNA was reverse transcribed using the SuperScript III-RT kit, as per the manufacturer’s recommendations. In a 25-μl reaction, the primary PCR was performed using 2.5 µl of 10 × diluted cDNA of pIC-stimulated spleen samples (equivalent to 25 ng initial RNA), ISPs (0.2 µM), and TopTaq DNA polymerase (0.625 U per reaction) following the manufacturer’s recommended protocol. Nested PCR was performed using 2.5 µl of 1:2 diluted cDNA of pIC-stimulated spleen samples, ISPs (0.2 µM), and TopTaq DNA polymerase. In the second round of PCR, 2.5 µl of the first-round PCR reaction was further amplified. For the full-length Gm-AID (*Gm-aicda*) isoform, both first and nested PCR reactions were incubated at 94 °C for 3 min, followed by 10 cycles of [94 °C for 30 s; 55 °C → 50 °C for 30 s, decreasing 0.5 °C per cycle; 72 °C for 90 s] and 25 cycles of [94 °C for 30 s; 50 °C for 30 s; 72 °C for 90 s] and 72 °C for 10 min. For truncated Gm-AID (*T-Gm-aicda*), 53 °C was used as the initial annealing temperature. PCR products were gel extracted and TA-cloned, and 10 colonies for each spleen sample and isoform were sequenced as detailed above. The Gm-AID cDNA accession number is OP856785.

### Delineation of Gm-aicda isoforms’ transcript expression in adult tissues, embryonic and early larval life stages

To investigate *Gm-aicda* tissue expression patterns, tissues from healthy individual adults were collected (two male fish: 758 and 1260 gr; two female fish: 1520 and 890 gr). Included tissues were blood, brain, eye, fin, gill, gonad, hindgut, midgut, heart, head kidney, posterior kidney, liver, dorsal muscle, ventral muscle, pyloric caecum, dorsal skin, ventral skin, spleen, and stomach. To assess *Gm-aicda* transcript expression during embryogenesis and early larval development, a mixture of fertilized eggs and cleavage-stage embryos were collected after communal spawning and distributed into three incubators, as described previously [116]. The collected floating fertilized eggs (0 days post-fertilization [DPF], i.e., day 0) were distributed into three 50-L conical incubator tanks (350 ml of eggs per tank). The tanks were kept at 5.5 to 6.1 °C, with a 25 L h − 1 flow rate, gentle aeration, and under an ambient photoperiod [116]. Using 500 μm Nitex, a mixture of ~ 180 eggs/embryos were daily collected from each tank, starting 12 h (i.e., 0 DPF) post-fertilization to 4 days post-hatch (i.e., 20 DPF). Samples were then flash frozen using liquid nitrogen. The blastula/gastrula stages were observed from day 1 to 6. The segmentation period started on day 7, and the golden eye stage was noticed on day 12. On day 15, hatching began and completed for all embryos on day 18 [117].

Total RNA was extracted from frozen samples using TRIzol reagent. Briefly, ~ 100 mg of flash frozen sample was disrupted by disposable pestles (Fisherbrand), or mortar and pestle (firm tissues, i.e., eye, gill, heart, stomach, pyloric caecum, midgut, hindgut, dorsal skin, ventral skin, dorsal muscle, ventral muscle, and fin; baked in at 220 °C for 5 h) in 1 ml of TRIzol reagent followed by further homogenization through QIAshredder spin columns (Qiagen). A standard TRIzol RNA extraction protocol was conducted. Liver samples were further purified through standard phenol–chloroform extraction and ethanol precipitation. Extracted RNA (30 µg) was purified using RNase-free DNase-I treatment (Qiagen) and column-purified by RNeasy MinElute Clean-up Kit (macrophage samples) or RNeasy Mini Kit (Qiagen; tissue panel and developmental samples). Quality and integrity of extracted RNA were assessed through agarose gel electrophoresis and Nanodrop spectrophotometry.

Since it has previously been shown that *ef1-α* (*elongation factor 1-α*) exhibits similar transcript expression in different Atlantic cod tissues, its transcript expression was studied alongside Gm-AID isoforms [118]. As positive controls for *Gm-aicda* transcript expression, we used splenic cDNA of immune-challenged individual Atlantic cod (24HPI). These samples were collected from different fish individual as a part of a previous study [114]. Since *aicda* is largely expressed in activated B cells, RNA obtained from pIC-stimulated Atlantic cod macrophages (at 24HPI) were used as a negative control. These samples were also collected from different fish individual as a part of a previous study [119]. cDNA synthesis was performed using M-MLV RT and 5 µg of clean total RNA. Two microliters of 1:10 diluted cDNA (equivalent to 50 ng of initial RNA) was amplified in a 25-µl reaction using Top Taq DNA polymerase and 0.2 µM *Gm-aicda* ISPs or *ef1-α* primers (Supplementary Table 6). PCR cycling conditions were an initial denaturation step for 5 min at 94 °C followed by 35 cycles of [30 s at 94 °C; 30 s at 54 °C; and 30 s at 72 °C] and 5 min at 72 °C. Amplicons were visualized on 2.5% agarose gel.

### Immune responsiveness of Gm-aicda transcript levels

To measure the changes in *aicda* transcription in response to immune stimulation, reverse transcription ‒ fluorescence-based quantitative real-time PCR (RT-qPCR) was performed. In these experiments, splenic clean RNA extracted from pIC, ASAL, and PBS-treated fish were used (intraperitoneally injected; 6HPI and 24HPI; 10 fish per treatment) [114, 115]. The total RNA was isolated, DNase treated, and cleaned up from each frozen sample as detailed above. M-MLV RT was used to synthesize cDNA using 5 µg of clean total RNA according to the manufacturer’s protocol. Primer quality control was conducted using splenic cDNA pool of pIC- and ASAL-stimulated samples. A 5-point and 1:3 dilution standard curve of cDNA (starting from 10 ng of input RNA) was used to test the quality and efficiency of primer pairs (Supplementary Table 6). Three fish per treatment and time point were used to select normalizers with stable expression. Two different sets of ISPs and 4 sets of normalizer primers were tested. The same ISPs as described above, along with gene-specific primers for *60S acidic ribosomal protein P1* (*rplp1*)[119] (geNorm M = 0.4150) and *ATP synthase H*^+^ *transporting, mitochondrial Fo complex, subunit F2* (*ATPS*) [114] (geNorm M = 0.4175), were qualified for qPCR analysis (Supplementary Table 6). Two microliters of 10 × diluted cDNA (10 ng input RNA) was amplified in a 13-µl reaction containing 6.5 µl of Power SYBR Green master mix (Applied Biosystems) and 0.52 µl of each primer (1.25 µM). qPCR assays were carried out using a ViiA7 System (Applied Biosystems, Thermo Fisher Scientific). Cycling conditions were one cycle of [2 min at 50 °C; 10 min at 95 °C], 40 cycles of [15 s at 95 °C; 30 s at 55 °C; 1 min at 60 °C]. The dissociation curves were also included to confirm the homogeneity of the qPCR products. The qPCR assays were performed in 384-well plates, and consistency of the assays between plates was checked using linker samples (C_T_ values were < 1 cycle between plates). All the samples, linkers, and no-template controls were carried out in triplicate.

To analyze qPCR results, ViiA 7 Software v1.2 (Applied Biosystems) was used. The expression of *Gm-aicda* isoforms (C_T_ values) were normalized to expression level of *rplp1* and *ATPS*, with incorporation of amplification efficiency of each primer pair. Then, relative quantity (RQ) of each transcript was calculated using a calibrator sample. For each transcript, the lowest expression sample was considered as the calibrator (RQ set as 1.0). Statistical analysis was conducted using IBM SPSS Statistics 20 software. Expression of Gm-AID isoforms at each immune-stimulated condition were compared to that of PBS-injected control using nonparametric *T*-test for independent samples.

### Purification and biochemical analysis of AID proteins

Extant and ancestral AID orthologs were expressed in the same pGEX5.3-based GST-fusion bacterial expression system and purified as described before and used to make several key discoveries on the biochemical properties of human and orthologous AID [25, 70–72]. Site-directed mutagenesis and PCR-based manipulation were conducted to create single point mutants and T-Gm-AID, respectively. For each of the 71 purified GST-AID enzymes used in this study, 2–6 independent protein preparations were purified [25, 70–72].

Briefly, a 500-ml culture of DE3 cells containing GST-AID expression vector was grown at 37 °C and 225 rpm in the presence of 100 µg/ml ampicillin. When the culture reached the log phase (an OD of 0.6), 1 mM of Isopropyl β-d-1-thiogalactopyranoside (IPTG) and 100 µg/ml ampicillin were added. Bacterial cultures were then incubated at 16 °C and 225 rpm for 16 h. The bacterial culture was centrifuged, and the pellet was resuspended in 20 ml of phosphate-buffered saline (PBS, Sigma) pH 7.5. Cells were lysed by a French Pressure cell and centrifuged to collect the supernatant. GST-AID was then column-purified from the supernatant of lysed cells using Glutathione Sepharose high-performance beads (Amersham) as per manufacturer’s recommendations. Briefly, the supernatant was applied twice to a purification column and washed with 50 ml of PBS, pH 7.5. GST-AID was eluted with elution buffer (50 mM Tris [pH 8.0] and 10 mM L-Glutathione reduced) into 0.5-ml fractions. The quantity of protein in each fraction was measured using NanoDrop spectrophotometry (ND-1000) and between four to five fractions containing > 0.5 mg/ml total protein were dialyzed overnight at 4 °C into the final storage buffer (20 mM Tris pH 7.5, 100 mM NaCl, and 1 mM dithiothreitol). Purified GST-AID was aliquoted into 50- to 100-µl aliquots, flash frozen, and stored at − 80 °C.

Eukaryotic expression of Gm-AID in HEK293T cells was also carried out as described [84, 120, 121]. Briefly, GST-AID fragment was inserted into pcDNA3.1-V5-6xHis-Topo vector and 5 µg of plasmid per plate was transfected into 10-cm plates of HEK 293 T cells (seeded with 5 × 10^5^ cells) using Polyjet transfection reagent (FroggaBio). Fifty plates were transiently transfected per GST-AID homolog. Following 48 h incubation at 37 °C, cells were resuspended in PBS (pH 7.5) containing 50 µg/ml RNase A (Invitrogen) and 0.2 mM phenylmethylsulfonyl fluoride (PMSF, Sigma). Cells were then lysed using a French Pressure cell. Samples were run through the French Pressure cell three times with a 30-min incubation at room temperature before the last run to allow the RNase A time to act. GST-AID was then purified from supernatant using Glutathione Sepharose high-performance beads (Amersham). Briefly, the supernatant was applied to the purification column twice and washed with 50 ml of PBS (pH 7.5) containing 0.2 mM PMSF. GST-AID was eluted off the beads using 50 mM Tris (pH 8) and 10 mM L-Glutathione reduced. 0.25-ml fractions were collected and analyzed by SDS-PAGE and stained with Coomassie blue. Fractions containing the band of interest (~ 48 kDa) were combined. Then, 5% glycerol and 50 µg/ml of bovine serum albumin (BSA, Invitrogen) were added before dialyzing the fractions overnight at 4 °C into the final storage buffer (20 mM Tris pH 7.5, 100 mM NaCl, 5% glycerol, and 1 mM dithiothreitol). Purified GST-AID was aliquoted into 50- to 100-µl aliquots, flash frozen, and stored at − 80 °C. Alternatively, beads with bound GST-AID were washed with PBS (pH 7.5) and stored in AID storage buffer as bead-bound AID. The quality and quantity of the purified prokaryotic and eukaryotic AID preparations were assessed using Coomassie staining and western blotting, respectively. In western blot analyses, anti-GST (SantaCruz) antibodies and Goat anti-Rabbit IgG (SantaCruz) were used as the primary and secondary antibodies.

To investigate the full spectrum of the biochemical properties of purified wild type and mutant AID, optimal temperature, time course, substrate specificity, enzyme kinetics, global ssDNA binding, and activity on 5-methylated cytidine (5-mC) were explored using established assays [70–72]. Data were graphed using GraphPad Prism 5 software (GraphPad, USA) and error bars were set to represent standard error. The statistical significance of the results was analyzed using one-way ANOVA (IBM SPSS Statistics 20, IBM Corp.). Experiments were conducted using 3–4 independent protein preparations of AID in 1–4 replicates.

To determine the optimal temperature of AID orthologs, 3 µl of purified GST-AID was incubated with 17 fmol of ^32^P-labelled TGCbub7 substrate at various temperature points (− 10 °C to 40 °C). To compare the catalytic rate of AID wildtypes and mutants through Michaelis–Menten kinetics, AID orthologs were incubated with a 0.0625–100 fmol range TGCbub7 substrate at their optimal temperature and pH. The results of the time course experiments were used to estimate the proper incubation time for each AID ortholog and mutant to ensure that the AID activity was within its initial velocity. Enzymatic velocity (fmol of deaminated product/min of incubation/µg of AID) was plotted against substrate concentration (nM). To estimate *K*_cat_, *K*_m_, and *V*_max_ parameters, the data was fitted into *Y* = Et × *K*_cat_ × *X*/(*K*_m_ + *X*) equation. This equation is a modified version of Michaelis–Menten kinetics where the *K*_cat_ can be calculated as well. In this equation, *Y* is the enzyme velocity, *X* is the substrate concentration, Et is the concentration of enzyme catalytic sites, *K*_cat_ is the number of times each enzyme site converts substrate to product per unit time (i.e., the turnover number), and *K*_m_ (i.e., the Michaelis–Menten constant) is the substrate concentration needed to achieve a half-maximum enzyme velocity (i.e.,* V*_max_). Since AID has one catalytic pocket, its Et is equal to the concentration of enzyme used in the experiment. To estimate the Et, the molecular weight of the GST-AID orthologs and mutants were calculated using Protein Molecular Weight web-based application (https://www.bioinformatics.org/sms/prot_mw.html).

To investigate substrate sequence specificity of Gm-AID, WRCbub7 (TGC, TAC, and AGC) or non-WRCbub7 substrates (GGC, GTC, and GAC) were incubated with 3 µl of AID orthologs at their optimal temperature and pH. Gm-AID, Ip-AID, Dr-AID, and Hs-AID were incubated for 96 h, 10 h, 20 min, and 3 h, respectively. The relative deamination efficiency for each substrate was calculated by dividing the average activity on the given substrate to the average activity of all substrates. Deamination activity of each AID ortholog on 5-mC was studied using 50 fmol of TG(mC)bub7, AG(mC)bub7, and GG(mC)bub7 which are substrates that contain a target 5-mC rather than dC [71].

Global ssDNA binding affinity of Gm-AID isoforms were compared to other AIDs using electrophoretic mobility shift assay (EMSA) [70]. Briefly, a 0.025–2.5 nM range of [TGCbub7] was incubated with 0.9 µg of purified AID in binding buffer (50 mM MgCl_2_; 50 mM NaCl; 1 mM DTT in 100 mM Phosphate buffer pH 7.21) for 1 h at their optimal temperature. Samples were then UV cross-linked on ice and electrophoresed on an 8% acrylamide native gel at 4 °C. Results were plotted as fmol bound substrate against nM of free substrate. To estimate half-saturation values, data was fitted into *Y* = *B*_max_*X/(*K*_d_ + *X*) equation where *Y* is the concentration of bound fraction, *X* is the concentration of free fraction, *B*_max_ is the maximum concentration of bound fraction, and *K*_d_ is the binding affinity of AID for the substrate.

### PCR-based AID activity assay

To compare the deamination activity of AID orthologs on various DNA sequence and secondary structure, a previously described PCR-based assay was conducted [21, 25]. Briefly, 200 ng of the substrate plasmid was denatured at 98 °C for 10 min in 100 mM phosphate buffer. Four microliters of purified AID and 1^−3^ U of uracil glycosylase inhibitor (UGI, New England Biolabs) were added to each reaction after snap-cooling in an ice bath (final volume: 10 µl). Samples were incubated for various time-points ranging from 1 to 16 h at optimal conditions of each AID ortholog. To detect AID-mutated plasmids, nested PCR using deamination-specific primers (Supplementary Table 6) was performed on serially diluted reactions (1/2 to 1/1,000,000). One microliter of each dilution was amplified under an initial denaturation step for 3 min at 96 °C followed by 30 cycles of [30 s at 96 °C; 30 s at 58 °C; and 1 min at 72 °C] and 10 min at 72 °C. One microliter of primary PCR product was then amplified under the same cycling conditions except that 57 °C was used as the annealing temperature. PCR products were analyzed on a 1.2% agarose gel.

### Structure prediction and AID-DNA binding simulations

We employed a similar structure prediction approach, as previously described [41, 81, 122]. Five APOBEC structures, and the recent partially truncated crystal AID structure were chosen as templates for homology modeling: mouse A2 NMR (PDB: 2RPZ), A3A NMR (PDB: 2M65), A3C (PDB: 3VOW), A3F-CTD X-ray (PDB: 4IOU), and A3G-CTD X-ray (PDB: 3E1U) [123–127]. The template AID/APOBEC structures were obtained from the protein databank (http://www.rcsb.org) and visualized using PyMOL v1.7.6 (http://www.pymol.org/). Using the default parameters of I-TASSER (http://zhanglab.ccmb.med.umich.edu/I-TASSER/), 125 models were constructed for each AID orthologs of which the best open conformations were chosen [128–130]. Ramachandran plots were created using Rampage and used to evaluate the quality of the proteins on an individual residue basis based on their stereochemical angles [131].

The catalytic pocket was defined by the indented space containing the Zn-coordinating and catalytic residues (Hs-AID: H56, E58, C87 and C90; Dr-AID: H60, E62, C99 and C102; Ip-AID: H59, E61, C98 and C101; Gm-AID: H60, E62, C100, C103). The catalytically accessible models were defined by accessibility of catalytic glutamate to the surface of the protein. For Hs-AID, Dr-AID, Ip-AID, and Gm-AID, respectively, 10, 12, 10, and 13 models were considered as open conformation. 5′-TTTGCTT-3′ ssDNA was chosen as our substrate, since it has been shown to be the preferred substrate of human and bony fish AID. To simulate AID-DNA binding, DNA substrate was docked to each AID model using Swiss-Dock (http://www.swissdock.ch) [132, 133]. The substrate was constructed using ChemDraw Prime v.16.0 (http://www.cambridgesoft.com/software/overview.aspx) and Marvin Sketch v.5.11.5 (http://www.chemaxon.com/products/marvin/marvinsketch/), while surface topology and docking parameters were generated using Swiss-Param (http://swissparam.ch) [134]. The 32 lowest-energy clusters were selected, thus representing 256 of the lowest-energy individual binding events for each AID. For each dock, we restricted the ssDNA binding within 30 × 30 × 30 Å (*x*, *y*, *z* coordinates) from the Zn-coordinating histidine. Each model was docked with a substrate 5 times creating 1600 clusters representing 13,250 conformations for Hs-AID and Gm-AID, 1920 clusters representing 15,900 conformations for Dr-AID, and 2080 clusters representing 17,225 conformations for Ip-AID. UCSF chimera v.1.11.2 (https://www.cgl.ucsf.edu/chimera) was used to view the conformations of substrate, and its interactions with AID models [135]. Deamination-conducive AID-DNA complexes were defined by the accessibility of the NH_2_-group of dC to the catalytic Zn-coordinating and glutamic acid residues. To analyze the interaction of each nucleotide with AID model, PyMol v1.3 (http://www.pymol.org/) was used to measure amino acid residues within 4 Å of the nitrogenous base and the 1st carbon of the deoxyribose sugar.

### Characterization of the cod IgVH region and WRC analysis

A partial immunoglobulin heavy chain locus of the Atlantic cod has previously been characterized (GenBank identifier: AJ871288.1). This sequence was BLAST aligned against the improved version of the Atlantic cod genome (gadMor2) using default parameters of blastn task in BLAST + program [38]. Complete protein sequences for IgM, IgD, and IgZ from GenBank were extracted to perform tblastn against the gadMor2 genome (Supplementary Table 7). Possible constant regions were identified manually from blast results and extracted from the genomic sequence, and a reciprocal blast was performed towards GenBank (blastx) to verify annotation. All sequences extracted from AJ871288.1 and gadMor2 genome were compared where the annotation from AJ871288.1 was preferred.

All variable (V) regions for the Atlantic cod were downloaded from NCBI and aligned against known variable regions of other bony fish (Supplementary Table 7). Sequences with similarity to other variable regions were blasted to gadMor2 genome with near to no restrictions. The blast output was sorted on scaffold/LG. Then, within each scaffold/LG, sequences were sorted on start position and *IgV* regions were extracted using bedtools software [136]. These sequences were aligned against reported cod variable regions in Mega software [137]. Reciprocal blast was used to filter out any non-*IgV* sequence. Short sequences that did not cover the entire variable region or contained many insertions or deletions were discarded. Cod *IgV* CDRs were mapped from *T. rubripes* Ig gene variable regions [138, 139]. The Atlantic cod *IgV*_*H*_ sequences were used to retrieve the *IgV*_*H*_ regions of other Gadidae species (the European Nucleotide Archive (ENA) accession number: PRJEB12469 and the Dryad repository: https://doi.org/10.5061/dryad.326r8).

For WRC motif analysis, Japanese puffer fish *IgV*_*H*_ (*Tr-IgV*_*H*_), and nurse shark *IgV*_*H*_ (*Gc-IgV*_*H*_) sequences were obtained from NCBI (Supplementary Table 7). The nurse shark complementarity-determining regions (CDRs) were mapped from *Tr-Ig* gene variable regions [138, 139]. *Hs-IgV*_*H*_, mouse *IgV*_*H*_ (*Mm-IgV*_*H*_), chicken *IgV*_*H*_ (*Gg-IgV*_*H*_*)*, South African toad *IgV*_*H*_ (*Xl-IgV*_*H*_), *Ip-IgV*_*H*_, salmon *IgV*_*H*_* (Ss-IgV*_*H*_), *Dr-IgV*_*H*_, and *Gm*-*IgV*_*H*_ sequences were obtained from IMGT (the international ImMunoGeneTics information system) database (http://www.imgt.org/). [140–148]. For these sequences, the CDRs and framework regions (FRs) were identified using IMGT database. In these analyses, the number of motifs were counted in each region using Python (Version 3.8) [149]. For WRC/GYW motifs, TGC, TAC, AGC, AAC, GCA, GTA, GCT, and GTT and for WGCW motifs AGCA, AGCT, TGCA, and TGCT were counted. Then, the sum of WRC/GYW or WGCW motifs for each region was divided to the number of nucleotides analyzed for that given region to normalize for the variation in the length of each region. The average of these normalized WRC/GYW or WGCW indexes were calculated for CDRs and FRs. The enrichment of the motifs in CDRs was estimated by dividing the average index of CDR 1 and 2 by the average index of FR 1, 2, and 3. Also, the GC content of the coding sequences was retrieved from Codon and Codon-Pair Usage Tables (CoCoPUTs) server [150]. This database is available on https://hive.biochemistry.gwu.edu/review/codon2.

### Ancestral sequence reconstruction

ASR methodology is comprised of four steps of (1) selecting extant species, (2) creating a multiple sequence alignment, (3) computing a phylogenetic tree, and (4) predicting the ancestral sequences. To infer the ancestral protein sequences of AID within and outside of the Gadiformes lineage, the orthologous aicda sequences were retrieved from 66 teleost genomes sequenced previously [33]. The Atlantic cod aicda gene locus (Ensemble gene identifier: ENSGMOG00000004114) was BLAST aligned against the assembled and raw genomic data of each species (the European Nucleotide Archive (ENA) accession number: PRJEB12469 and the Dryad repository: https://doi.org/10.5061/dryad.326r8) using default parameters of blastn task in BLAST + program [33]. The genomic region was then retrieved as the aicda locus. The *aicda* mRNA transcript was then predicted using the AUGUSTUS server (http://bioinf.uni-greifswald.de/augustus/submission.php) [151]. The initiation codon, coding sequence, and the stop codon for identified *aicda* transcripts were confirmed using the ATGpr website (https://atgpr.dbcls.jp/) [152]. In total, the *aicda* gene sequence from 73 species (74 gene sequences) was used to perform ASR analyses (Supplementary Data 2).

The amino acid multiple alignments were built based on our predicted structure of Gm-AID (Supplementary Data 3) using the PROMALS3D web interface (http://prodata.swmed.edu/promals3d/promals3d.php) [153]. The generated amino acid alignment was then used to guide the nucleotide sequences alignment using the TranslatorX server (http://translatorx.co.uk) [154]. Since the accuracy of the MSA impacts the ASR results [155], the final nucleotide and amino acid alignments were manually inspected to assure the quality of the alignment.

Another important factor contributing to the accuracy of ASR results is the topology of the phylogenetic tree [155–157]. We used the previously estimated species tree for our dataset as the start tree in ASR calculations [33]. As an outgroup, Lampetra tridentata CDA1 cytidine deaminase gene was used.

We applied three approaches to predict the ancestral states (Supplementary Data 4). First, we used RAxML, which is based on the protein alignment and takes advantage of the ML algorithm [95]. Second, we used the ProtASR package to infer the ancestral sequences based on protein structure and ML algorithms [96, 97]. Finally, we used MrBayes software to predict ancestral states based on the protein alignment and Bayesian statistics [90–94].

We used RAxML package version 8.2.9. First the best substitution model was selected. The GTRCAT substitution model (i.e., the General Time Reversible model with the CAT model of rate heterogeneity) gave us the highest ML in the model test runs. Then, the initial rearrangement settings (i.e., -i) and the number of categories (i.e., -c) were calculated. The best ML tree and bootstrap values were estimated using -i 10 and -c 55. Therefore, ancestral sequences were predicted using the GTRCAT substitution model, -i 10, -c 55, and the best ML tree obtained in this thesis or the species tree previously published.

In ASR analyses using MrBayes version 3.2.7, we used the GTR model with Gamma distribution of rate variation. Additionally, the 1st, 2nd, and 3rd nucleotide positions of a codon were unlinked. Each run was continued until the standard deviation of split frequencies of 0.01 or less was achieved, and the potential scale reduction factor (PSRF) for all parameters was reasonably close to 1.0. The previously published species tree [33] was used as the start tree for the MyBayes analyses. Proper tree topology constraints were defined to infer the ancestral sequence of the desired node. For each ancestral node, analyses were run four independent times, summed up, and reported as the results.

In ASR analyses using ProtASR versions 2.0 and 2.2 [96, 97], we used our computationally predicted Gm-AID 3D structure and the previously published species tree. Since the length of the alignment was different from the length of the PDB file, we used version B of ProtASR. Unlike other ASR frameworks, ProtASR implements a structurally constrained substitution model of evolution called “Mean-field”.

The results of RAxML, ProtASR, and MrBayes were compared. The consensus ancestral sequences were obtained with more weight on the MrBayes results since the previous studies concluded that Bayesian inference with rate variation model might outperform other methods [98, 99]. For any amino acid position with ambiguity above 0.2, any prediction above 0.2 was synthesized as a mutant.

## Supplementary Information

**Additional file 1: Supplementary Figure 1.** Comparison of the *aicda* genomic structure amongst vertebrates. **Supplementary Figure 2.** Comparison of the *aicda* synteny amongst vertebrates. **Supplementary Figure 3.** Atlantic cod AID purification and enzymatic characterization. **Supplementary Figure 4.** Expression and testing of Gm-AID produced in HEK293T cells. **Supplementary Figure 5.** Deciphering the basis of the absolute catalytic death of the polar cod AID. **Supplementary Figure 6.** Amino acid alignment of extant AIDs used for ASR analyses and predicted ancestral sequences. **Supplementary Figure 7.** Determination of the basic biochemical properties of resurrected ancestral AIDs to determine conditions for measurement of catalytic efficiency. **Supplementary Table 1.** Comparison of DNA interaction with substrate binding grooves on the surface of AID orthologs. **Supplementary Table 2.** Comparison of Gm-AID^H136^ residue in interaction with -1 position nucleotide upstream of the target dC and total interactions with substrate to its equivalent residue in other AID orthologs. **Supplementary Table 3.** WRC/GYW enrichment in complementarity determining regions (CDRs) *vs.* frameworks (FRs) of *IgV*_*H*_ genes of various Gadidae and vertebrate species. **Supplementary Table 4.** WGCW enrichment in complementarity determining regions (CDRs) *vs.* frameworks (FRs) of *IgV*_*H*_ genes of various Gadidae and vertebrate species. **Supplementary Table 5.** AID hotspot abundance in the entire *IgV*_*H*_ genes and GC content of annotated complete protein coding genes (CDSs) of various Gadidae and vertebrate species. **Supplementary Table 6.** The sequence of primers used in this study. **Supplementary Table 7.** GenBank accession number of the *teleost aicda and *Ig genes used in this *study.*

**Additional file 2: Supplementary Data 1.** computationally predicted 3D structure of Gm-AID used to guide amino acid alignment and as the structure template in ProtASR analyses.

**Additional file 3: Supplementary Data 2.** Aligned nucleotide sequence of genes used in ancestral sequence reconstruction analyses.

**Additional file 4: Supplementary Data 3.** The Python code used to assign and RGB color to each AID enzyme based on their catalytic efficiency.

**Additional file 5: Supplementary Data 4.** The combined scripts, input files, and setting files used to predict ancestral sequences using RAxML, MrBayes, and ProtASR packages.

## Data Availability

The datasets supporting the conclusions of this article are included within the article, its additional supplementary figures, and supplementary data files. The raw data used for enzyme assay analysis (e.g., quantitated enzyme assay gels and excel files) and reagents are available by request to the corresponding author. The accession numbers for the *aicda* sequences of the 67 analyzed teleost species as well as the teleost Ig sequences used for WRC enrichment analyses are available in supplementary table 7. The Gm-AID cDNA sequence reported in this study is available at Genbank with the accession number OP856785 [158].

## Abbreviations

UTR: Untranslated region
5-mC: 5-Methylcytidine
*aicda*: Activation-induced cytidine deaminase gene
AID: Activation-induced cytidine deaminase
APOBEC: Apolipoprotein B-mRNA-editing enzyme catalytic polypeptide-like complex family of cytidine deaminases
ASAL: Formalin-killed typical *A. salmonicida*
ASR: Ancestral sequence reconstruction
BCR: B cell receptor
C: Immunoglobulin constant domain
CDR: Complementarity-determining region of antibodies
C_H_: Heavy chain constant gene
CSR: Class switch recombination
dC: Deoxycytidine
ds: Double-stranded
DSBs: Double-stranded breaks in DNA
EMSA: Electrophoretic mobility shift assay
HIGM: Hyper-IgM syndrome
Ig: Immunoglobulin
*K*_d_: Dissociation constant
MHC: Histocompatibility complex
NLS: Nuclear localization sequence
NLS: Nuclear localization signal
NMR: Nuclear magnetic resonance
PCR: Polymerase chain reaction
PDB: Protein databank
pIC: Polyinosinic:polycytidylic acid
RACE: Rapid amplification of cDNA ends
RAG: Recombination activating gene
RT-PCR: Reverse transcription polymerase chain reaction
S: Immunoglobulin switch region
UNG: Uracil-N-glycosylase
V: Immunoglobulin variable domain
*V*_H_: V region heavy chain
*V*_L_: V region light chain
