# Supplementary material for: Ancestral reconstruction reveals catalytic inactivation of activation-induced cytidine deaminase concomitant with cold water adaption in the Gadiformes bony fish
Source: BMC Biol. 2022 Dec 27;20:293. doi: 10.1186/s12915-022-01489-8 (PMC9795746; doi:10.1186/s12915-022-01489-8)
Supplement: Supplementary file 1 — Additional file 1: Supplementary Figure 1. Comparison of the aicda genomic structure amongst vertebrates. Supplementary Figure 2. Comparison of the aicda synteny amongst vertebrates. Supplementary Figure 3. Atlantic cod AID purification and enzymatic characterization. Supplementary Figure 4. Expression and testing of Gm-AID produced in HEK293T cells. Supplementary Figure 5. Deciphering the basis of the absolute catalytic death of the polar cod AID. Supplementary Figure 6. Amino acid alignment of extant AIDs used for ASR analyses and predicted ancestral sequences. Supplementary Figure 7. Determination of the basic biochemical properties of resurrected ancestral AIDs to determine conditions for measurement of catalytic efficiency. Supplementary Table 1. Comparison of DNA interaction with substrate binding grooves on the surface of AID orthologs. Supplementary Table 2. Comparison of Gm-AIDH136 residue in interaction with -1 position nucleotide upstream of the target dC and total interactions with substrate to its equivalent residue in other AID orthologs. Supplementary Table 3. WRC/GYW enrichment in complementarity determining regions (CDRs) vs. frameworks (FRs) of IgVH genes of various Gadidae and vertebrate species. Supplementary Table 4. WGCW enrichment in complementarity determining regions (CDRs) vs. frameworks (FRs) of IgVH genes of various Gadidae and vertebrate species. Supplementary Table 5. AID hotspot abundance in the entire IgVH genes and GC content of annotated complete protein coding genes (CDSs) of various Gadidae and vertebrate species. Supplementary Table 6. The sequence of primers used in this study. Supplementary Table 7. GenBank accession number of the teleost aicda and Ig genes used in this study. [file 12915_2022_1489_MOESM1_ESM.docx]

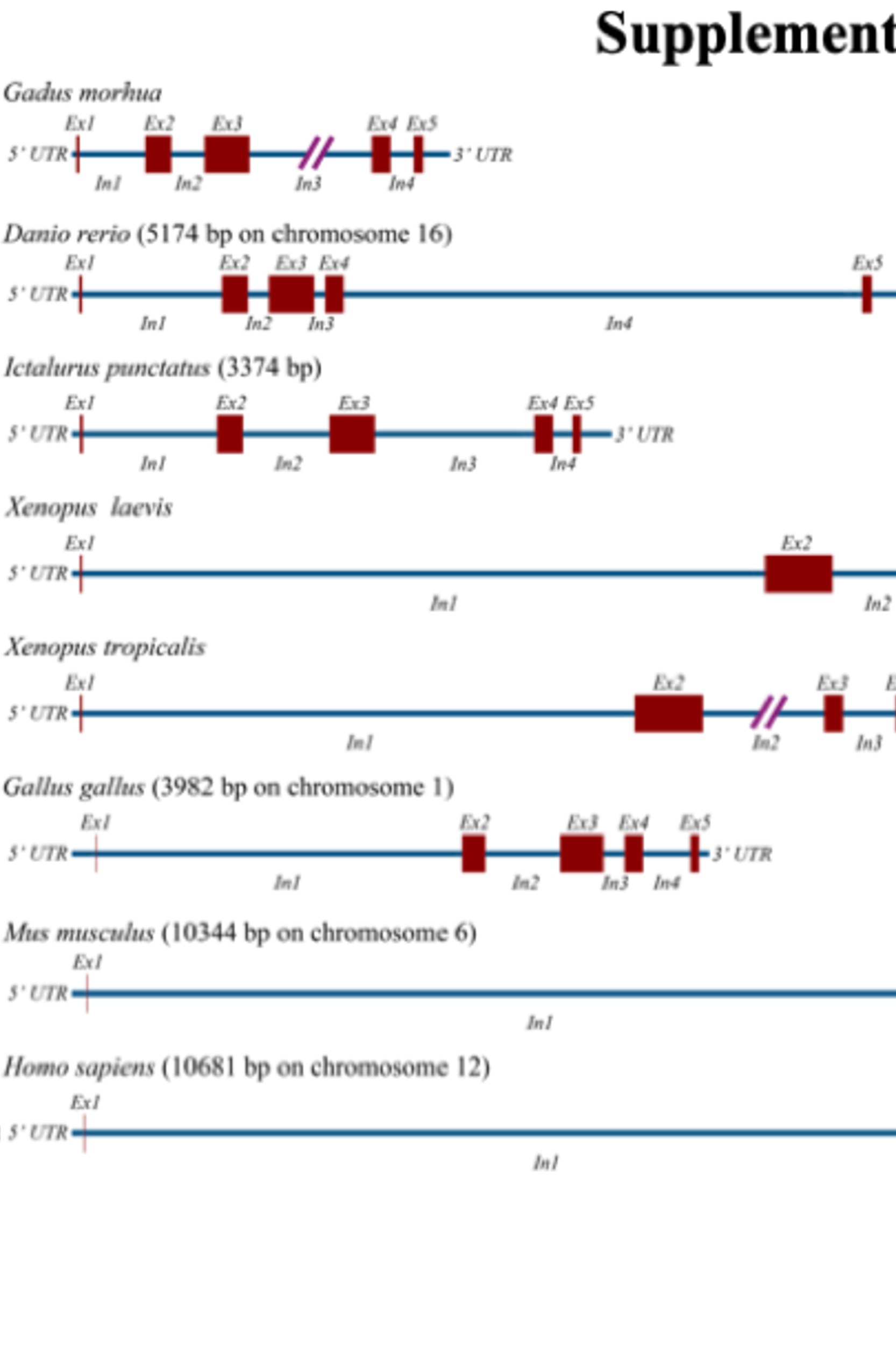


| Isoform | # of ATG from 5ʹ end | Reliability  score | Identity to Kozak rule A/GXXATGG | Start (bp) | Finish (bp) | ORF length (aa) | Stop codon found? | Protein sequence |  |
| --- | --- | --- | --- | --- | --- | --- | --- | --- | --- |
| *Gm-aicda* | 2 | 0.35 | AXXATGa | 28 | 666 | 213 | Yes | MISKLDSVLLAQKKFIYNYKNMRWAKGRNETYLCFVVKRRLGPDSLSFDFGHLRNRTGCHAELLFLSYLGALCPGLWGCADDRNRRLSYSVTWFCSWSPCANCATTLTRFLRQTPNLRLRIFVSRLYFCDLEGSPHVEGLRDLRRAGVQVKVMSYKDYFYCWQTFVAHRLSRFKAWEGLHTNYVRLSRKLNRILQPCETEDLRDVFRLFGLLT |  |
| *T-Gm*-*aicda* | 7 | 0.23 | AXXATGc | 152 | 727 | 192 | Yes | MRWAKGRNETYLCFVVKRRLGPDSLSFDFGHLRNRTGCHAELLFLSYLGALCPGLWGCADDRNRRLSYSVTWFCSWSPCANCATTLTRFLRQTPNLRLRIFVSRLYFCDLEGSPHVEGLRDLRRAGVQVKVMSYKDYFYCWQTFVAHRLSRFKAWEGLHTNYVRLSRKLNRILQPCETEDLRDVFRLFGLLT |  |
| Abbreviations: *Gm*-*aicda*: Atlantic cod *aicda*; *T-Gm-aicda*: Atlantic cod truncated *aicda* isoform. | | | | | | | | | |

Supplementary Figure 1. Comparison of the *aicda* genomic structure amongst vertebrates. The top panel shows the proportional schematic of the *aicda* locus exon-intron structure. *Aicda* locus of Atlantic cod (*Gadus morhua*), zebrafish (*Danio rerio*), channel catfish (*Ictalurus punctatus*), African clawed frog (*Xenopus laevis*), tropical clawed frog (*X. tropicalis*), chicken (*Gallus gallus*), mouse (*Mus musculus*), and human (*Homo sapiens*) were retrieved from the NCBI and Ensembl database. Exons and introns are shown as red boxes and blue lines, respectively. Discontinued lines represent introns or untranslated regions (UTRs) that are not fully sequenced. The bottom panel shows the predicted amino acid sequence of the open reading frame of the aicda transcripts.

_
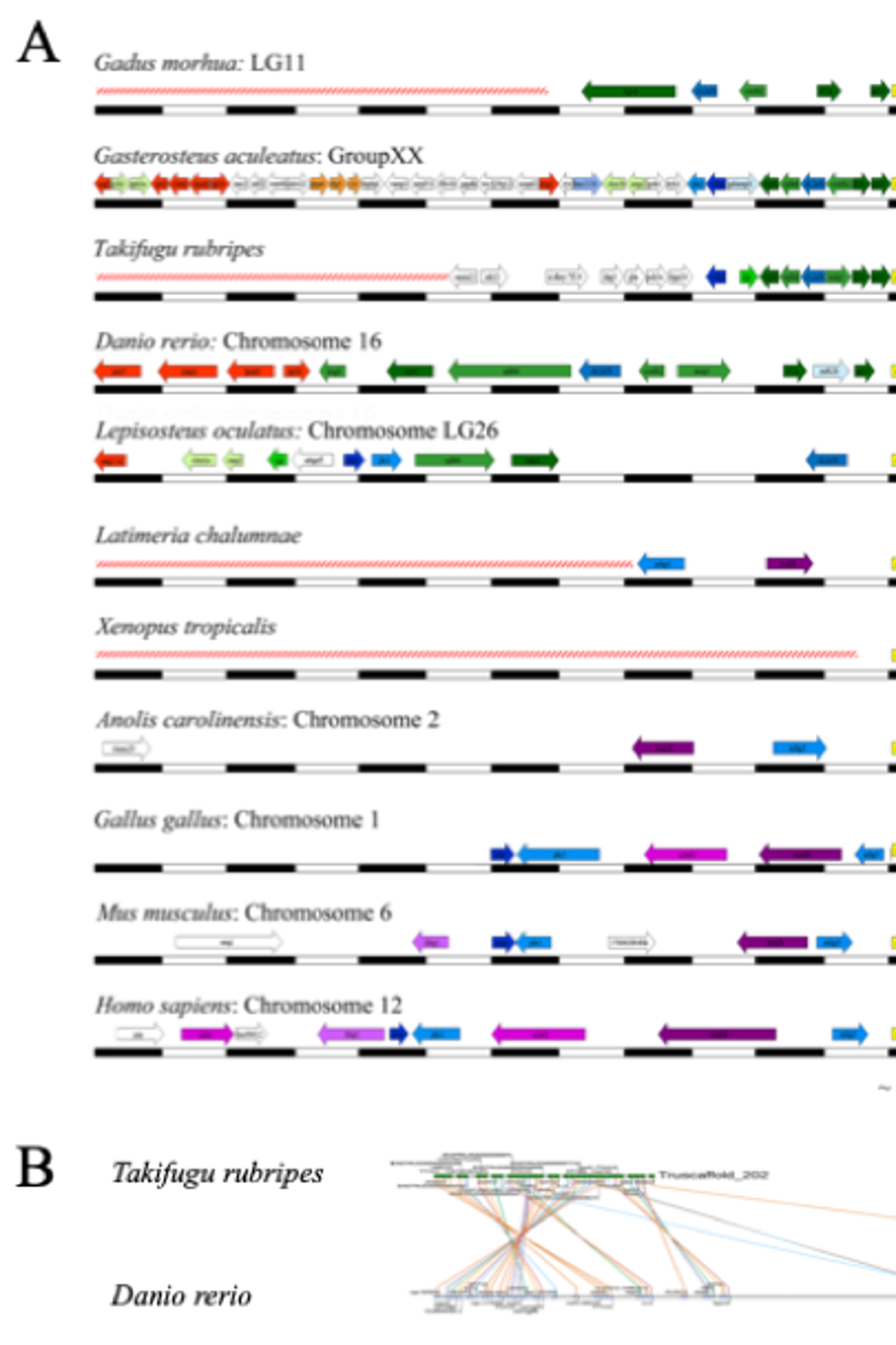
_

**Supplementary Figure 2. Comparison of the *aicda* synteny amongst vertebrates.** (**A**) Approximately 1 Mb region surrounding the *aicda* locus (colored in yellow) of Atlantic cod (*Gadus morhua*), three-spined stickleback (*Gasterosteus aculeatus*), Japanese pufferfish (*Takifugu rubripes*), zebrafish (*Danio rerio*), spotted gar (*Lepisosteus oculatus*), coelacanth (*Latimeria chalumnae*), green anole (*Anolis carolinensis*), chicken (*Gallus gallus*), mouse (*Mus musculus*), and human (*Homo sapiens*) were retrieved from the Ensembl database. *Aicda* genomic region of tropical clawed frog (*Xenopus. tropicalis*) was retrieved from Xenbase database. The retrieved region was manually analyzed to draw this figure. Red diagonal striped lines represent regions of genomic DNA with no sequencing data available. Genes conserved in all vertebrates, or only in tetrapods, or in bony fish are colored blue, violet, or green, respectively. Genes colored different shades of orange represent those found in select bony fish and amphibian species. (**B**) *Aicda* synteny analysis was performed using synteny database (<http://syntenydb.uoregon.edu/synteny_db/>) -based on Ensemble version 70 dataset. Using default parameters, *Dr-aicda* chromosomal location was compared to that of the Japanese pufferfish, the three-spined stickleback, the spotted gar, mouse, and human. Also, *Hs-aicda* synteny was compared to that of mouse and the spotted gar. The tropical clawed frog *aicda* synteny was compared with the human.


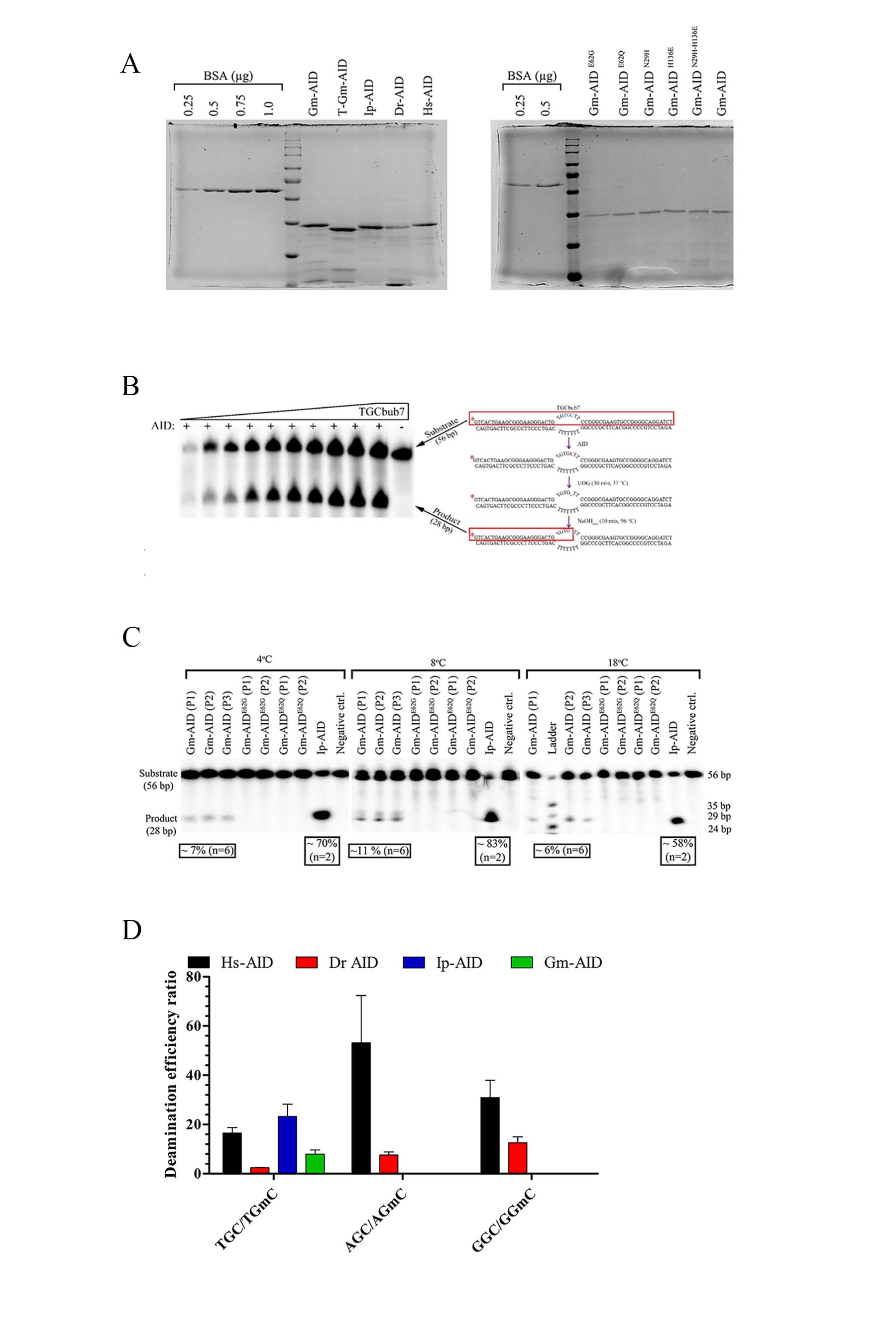


**Supplementary Figure 3. Atlantic cod AID purification and enzymatic characterization.** (**A**) Representative coomassie-stained SDS protein electrophoresis gels. After expression of GST-AID in bacteria (*E. coli*) and purification of GST-AID protein through GST affinity column, purity and yield of GST-AID were assessed by coomassie-stained SDS protein electrophoresis in comparison to BSA standards. (**B**) Experimental scheme for standard alkaline cleavage assay. TGCbub7 denotes a substrate bearing the WRC motif TGC located in a seven-nucleotide-long bubble region. The right panel shows the scheme for the standard alkaline cleavage assay. The left panel shows a representative denaturing acrylamide gel. The AID activity is reported as the percentage of initial substrate which was converted into product. **(C)** *Bona* *fide* cytidine deaminase activity of Atlantic cod AID. To confirm that the unusually low activity of Gm-AID is *bona* *fide* cytidine deaminase activity, wild type Gm-AID catalytic activity was compared to that of two mutants targeting essential catalytic pocket cytidine deamination residues (E62G and E62Q). Two independent protein preparations of each mutant were tested at 4, 8, and 18 °C for 63 h. Ip-AID was tested as a positive control. (**D**) Atlantic cod AID activity on 5-mC. To examine AID deamination activity on methylated cytidine, activity of AID orthologs on TGCbub7, AGCbub7, and GGCbub7 were compared to that of TGmCbub7, AGmCbub7, and GGmCbub7. Two independent protein preparations of each AID ortholog were tested in duplicate at their corresponding optimal temperature (*i.e.,* 31, 25, 14, and 8 °C for Hs-AID, Dr-AID, Ip-AID, and Gm-AID, respectively). The incubation time was set at 30 min, 3 h, 10 h, and 96 h for Dr-AID, Hs-AID, Ip-AID, and Gm-AID, respectively. Consistent with previous publications, Dr-AID showed the highest efficiency in deaminating 5-mC; while, Ip-AID and Gm-AID showed low activity on 5-mC, only on TGmCbub7 substrate. Data is represented as mean ± SEM (n=4)


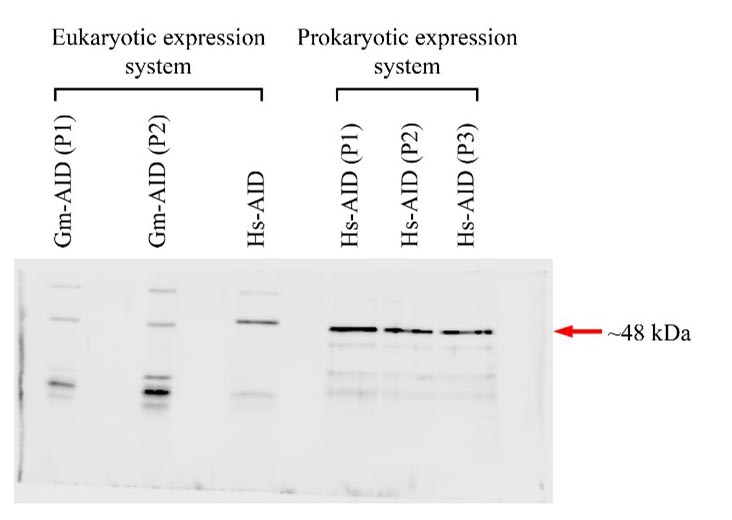

Supplementary Figure 4. Expression and testing of Gm-AID produced in HEK293T cells. To exclude the effect of expression system in our analysis, we expressed AID in HEK 293T cells. The top panel shows a representative western blot. After expression of GST-AID in human embryonic kidney cells 293 (HEK239T) and purification of GST-AID protein through GST affinity batch binding, purity and yield of GST-AID were assessed using western blotting. AID was probed with anti-GST (SantCruz) antibodies, followed by the secondary detection by Goat anti-Rabbit IgG (SantaCruz). The bottom panel shows the cytidine deaminase activity of GST-AID expressed in this system tested using the standard alkaline cleavage assay. GST-AID was analyzed in the form of cell lysate, purified on GST beads, or eluted from GST beads. AIDs were incubated with TGCbub7 substrate for various time point at 8 or 18°C.

**
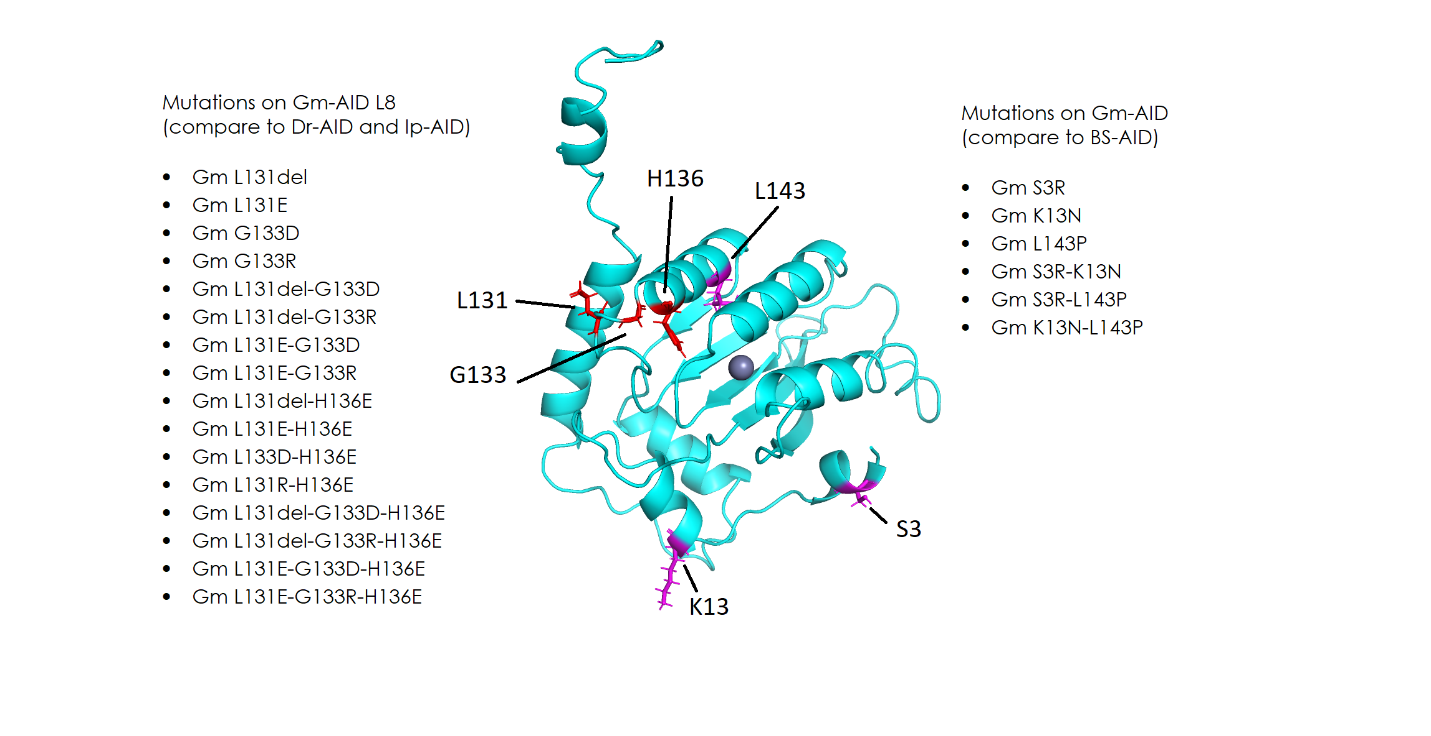

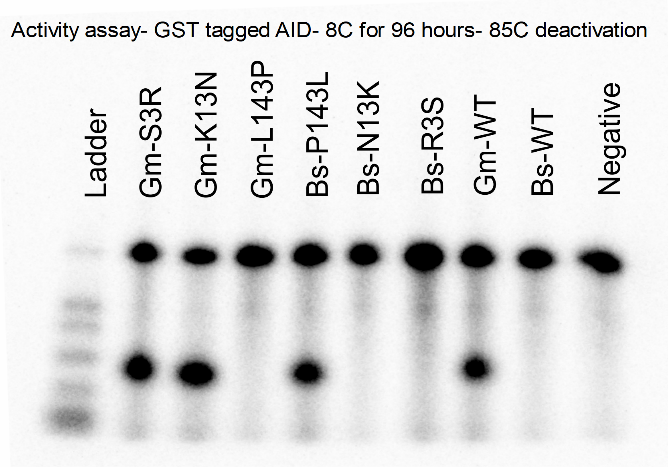
**

**Supplementary Figure 5. Deciphering the basis of the absolute catalytic death of the polar cod AID.** Residues that were different between the polar cod AID (Bs-AID) and the Atlantic cod AID (Gm-AID) were mutated in each protein to that of the other followed by testing the catalytic activity. One residue P143 was found to be responsible for the difference between the two enzymes and the absolute catalytic inactivity of Bs-AID.


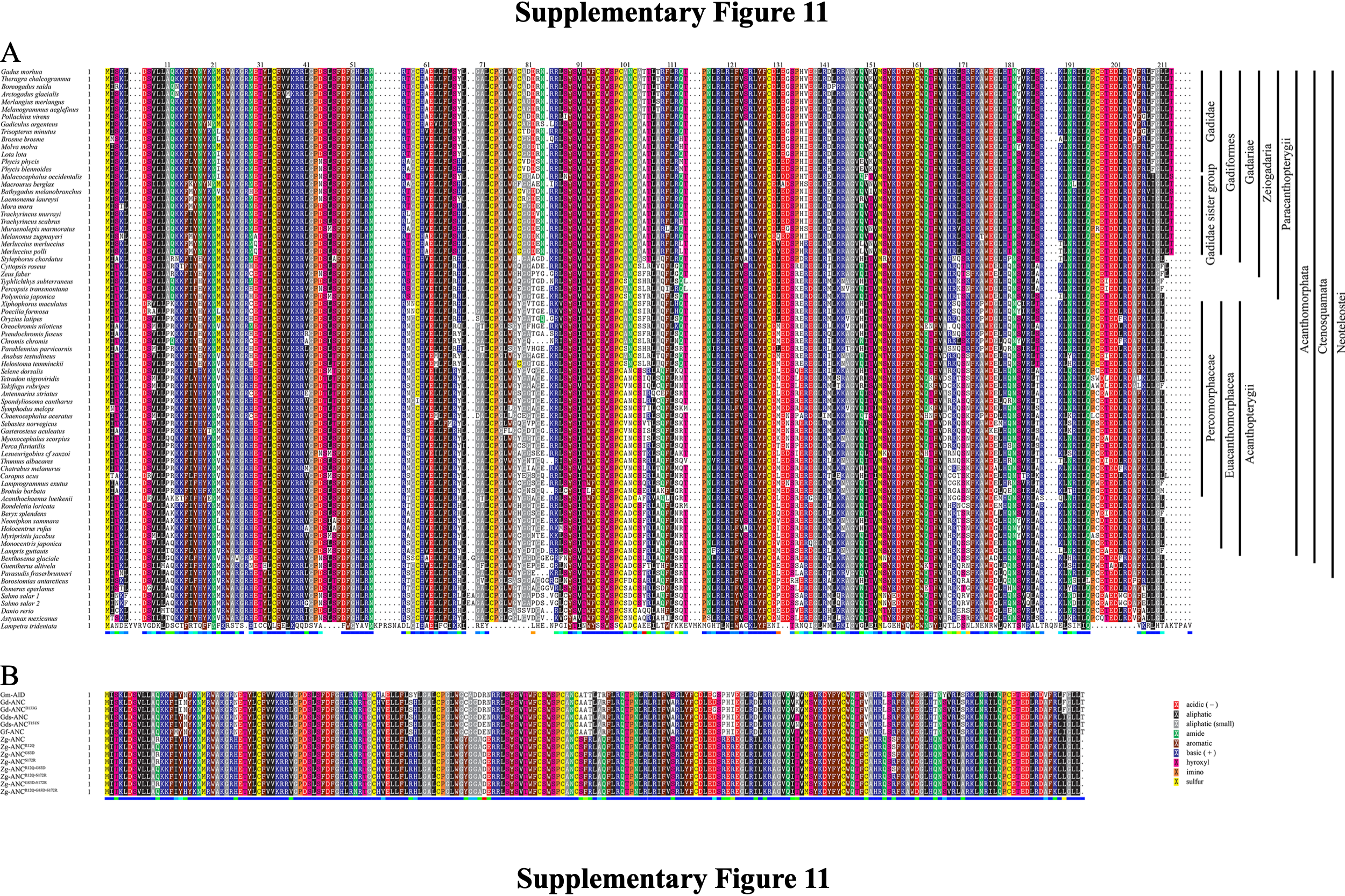


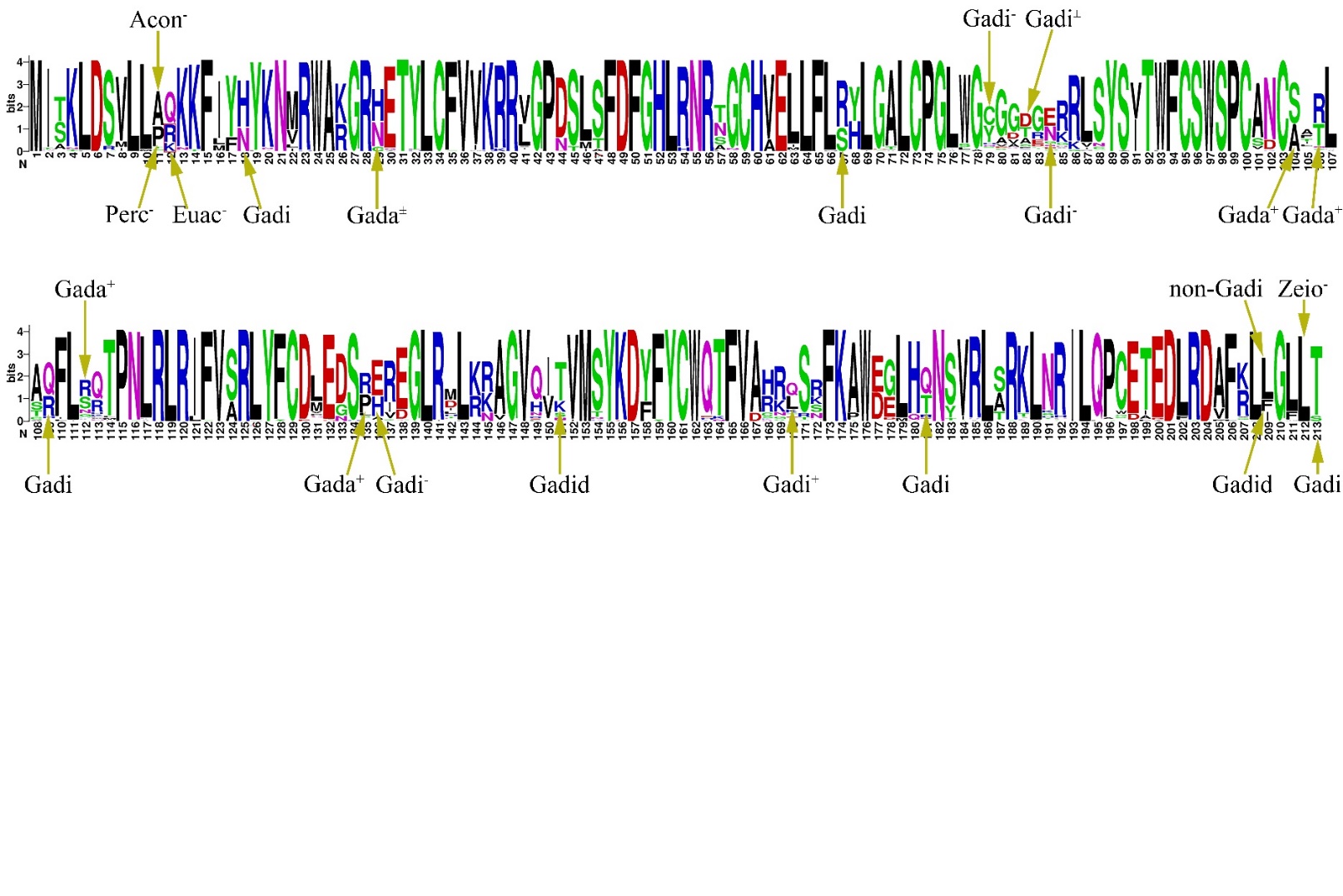


Supplementary Figure 6. Amino acid alignment of extant AIDs used for ASR analyses and predicted ancestral sequences. (A) 73 extant AIDs from 72 species within and outside of Gadiformes lineage were selected. Lamprey’s CDA1 (*L. tridentata*; AID-like protein) was used as an outgroup. The taxonomy information is shown on the right. (B) The top panel shows the amino acid alignment of the expressed ancestral AIDs. Ancestral AIDs were predicted using three methods of ancestral sequence reconstruction (ASR) implemented in MrBayes (Bayesian inference), RAxML (maximum likelihood based on nucleotide multiple alignment), and ProtASR (maximum likelihood based on nucleotide multiple alignments and the predicted 3D structure of Gm-AID) packages. The consensus sequences were chosen with higher emphasis on MrBayes results. AID sequence of four ancestral nodes were predicted: the common ancestor of Gadidae group (Gd-ANC); the common ancestor of Gadidae sister group (Gds-ANC); the common ancestor of Gadiformes lineage (Gf-ANC); and the common ancestor of Zeiogadaria group (Zg-ANC). Amino acids are colored based on their chemical properties as indicated in the bottom right corner legends. The arbitrary cut-off of 0.2 was used to generate variants where a position was predicted with ambiguity. The bottom panel shows the Amino acid conservation of extant AID homologs used in ASR analyses. Amino acid positions where a distinctive difference between various groups was observed are labeled. + sign emphasizes that a few members of other groups also contain the labeled amino acid at that position. – sign means that a few of the specified group are exceptions. ± sign indicates that while a few members of the specified group are exception, a few members of other groups show the spe­­­cified amino acid at the labeled position. Abbreviation: Acon: Acanthomorphata; Euac: Euacanthomorphacea; Perc: Percomorphaceae; Zeio: Zeiogadaria; Gada: Gadariae; Gadi: Gadiformes; non-Gadi: non-Gadiformes; Gadid: Gadidae.


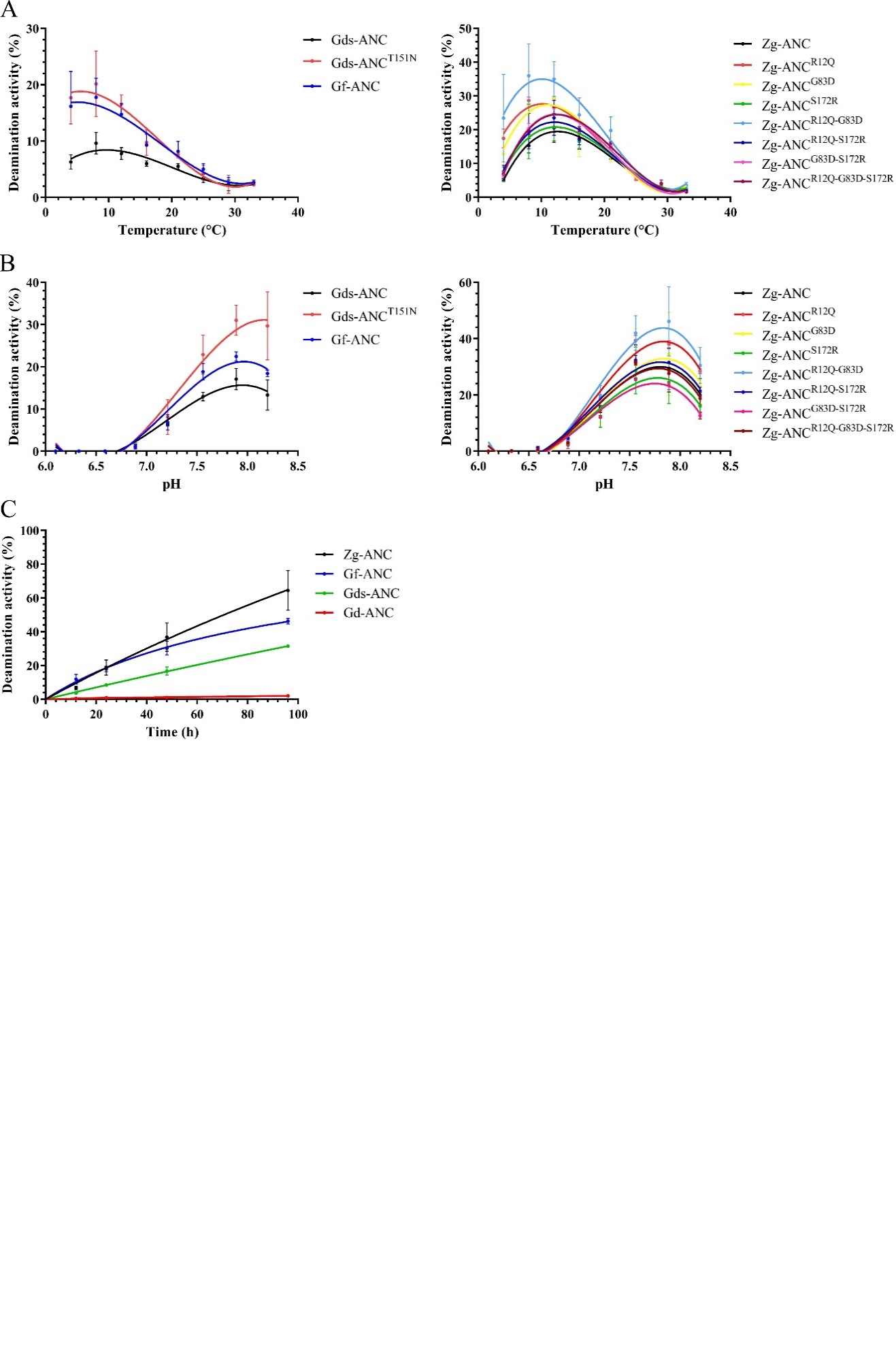


Supplementary Figure 7. Determination of the basic biochemical properties of resurrected ancestral AIDs to determine conditions for measurement of catalytic efficiency. Optimal temperature (A), optimal pH (B), and time-course kinetic (C) of predicted ancestral AIDs were measured using our standard alkaline cleavage assay. Two independent protein preparations of each ancestral AID were tested in duplicate. Data is presented as Mean ± SEM (n ≥ 4). Abbreviations: Gd-ANC: Gadidae ancestor; Gds-ANC: Gadidae sister group ancestor; Gf-ANC: Gadiformes ancestor; and Zg-ANC: Zeiogadaria ancestor.

**Supplementary Table 1.** Comparison of DNA interaction with substrate binding grooves on the surface of AID orthologs

|  | Hs-AID (%) | Dr-AID (%) | Ip-AID (%) | Gm-AID (%) |
| --- | --- | --- | --- | --- |
| ssDNA binding groove 1 | 75.53 | 38.71 | 54.22 | 48.39 |
| ssDNA binding groove 2 | 7.45 | 29.03 | 30.12 | 16.13 |
| ssDNA binding groove 1 and 2 | 7.45 | 19.35 | 8.43 | 14.52 |
| Direct involvement of α4 | 8.51 | 6.45 | 6.02 | 20.97 |
| Abbreviations: Gm-AID: Atlantic cod AID; Dr-AID: zebrafish AID; Ip-AID: channel catfish AID; Hs-AID: human AID. | | | | |

**Supplementary Table 2.** Comparison of Gm-AID^H136^ residue in interaction with -1 position nucleotide upstream of the target dC and total interactions with substrate to its equivalent residue in other AID orthologs

|  | Interaction with G in TGC motif (%) | Total interactions with substrate (%) |
| --- | --- | --- |
| Hs-AID^E122^ | 5.319% | 18.685% |
| Dr-AID^E135^ | 3.226% | 9.677% |
| Ip-AID^E134^ | 2.410% | 28.916% |
| Gm-AID^H136^ | 16.129% | 53.226% |
| Abbreviations: Gm-AID: Atlantic cod AID; Dr-AID: zebrafish AID; Ip-AID: channel catfish AID; Hs-AID: human AID. | | |

**Supplementary Table 3.** WRC/GYW enrichment in complementarity determining regions (CDRs) *vs.* frameworks (FRs) of *IgV_H_* genes of various Gadidae and vertebrate species

|  | FR1 | | | CDR1 | | | FR2 | | | CDR2 | | | FR3 | | |  |  |  |
| --- | --- | --- | --- | --- | --- | --- | --- | --- | --- | --- | --- | --- | --- | --- | --- | --- | --- | --- |
|  | # AID hotspots | # nt. analyzed | Index* | # AID hotspots | # nt. analyzed | Index* | # AID hotspots | # nt. analyzed | Index* | # AID hotspots | # nt. analyzed | Index* | # AID hotspots | # nt. analyzed | Index* | Ave. FRs | Ave. CDRs | CDRs/FRs |
| *Ag-IgV_H_* | 879 | 5905 | 0.15 | 224 | 1328 | 0.17 | 168 | 1863 | 0.09 | 114 | 1968 | 0.06 | 1186 | 7224 | 0.16 | 0.13 | 0.11 | 0.84 |
| *Bs-IgV_H_* | 405 | 2362 | 0.17 | 84 | 465 | 0.18 | 77 | 660 | 0.12 | 38 | 672 | 0.06 | 375 | 2201 | 0.17 | 0.15 | 0.12 | 0.78 |
| *Ma-IgV_H_* | 167 | 882 | 0.19 | 35 | 156 | 0.22 | 23 | 231 | 0.10 | 21 | 240 | 0.09 | 128 | 775 | 0.17 | 0.15 | 0.16 | 1.03 |
| *Ga-IgV_H_* | 226 | 1603 | 0.14 | 74 | 422 | 0.18 | 63 | 645 | 0.10 | 36 | 729 | 0.05 | 407 | 2407 | 0.17 | 0.14 | 0.11 | 0.83 |
| *Llo-IgV_H_* | 345 | 1935 | 0.18 | 76 | 357 | 0.21 | 37 | 495 | 0.07 | 44 | 528 | 0.08 | 253 | 1566 | 0.16 | 0.14 | 0.15 | 1.07 |
| *Pp-IgV_H_* | 377 | 2207 | 0.17 | 107 | 450 | 0.24 | 40 | 623 | 0.06 | 56 | 670 | 0.08 | 356 | 2042 | 0.17 | 0.14 | 0.16 | 1.18 |
| *Gadinae* | 2467 | 18589 | 0.13 | 852 | 4744 | 0.18 | 694 | 9009 | 0.08 | 383 | 5598 | 0.07 | 4051 | 25372 | 0.16 | 0.12 | 0.12 | 1.01 |
| *Gadidae* | 3189 | 22731 | 0.14 | 1035 | 5551 | 0.19 | 771 | 10127 | 0.08 | 483 | 6796 | 0.07 | 4660 | 28980 | 0.16 | 0.13 | 0.13 | 1.02 |
| *Gm-IgV_H_* | 790 | 7837 | 0.10 | 435 | 2373 | 0.18 | 363 | 5610 | 0.06 | 174 | 1989 | 0.09 | 1955 | 12765 | 0.15 | 0.11 | 0.14 | 1.27 |
| *Ip-IgV_H_* | 652 | 7199 | 0.09 | 482 | 2709 | 0.18 | 662 | 5498 | 0.12 | 379 | 2360 | 0.16 | 1753 | 12381 | 0.14 | 0.12 | 0.17 | 1.44 |
| *Tr-IgV_H_* | 309 | 3675 | 0.08 | 219 | 1245 | 0.18 | 183 | 2361 | 0.08 | 268 | 1215 | 0.22 | 803 | 5517 | 0.15 | 0.10 | 0.20 | 1.94 |
| *Dr-IgV_H_* | 410 | 5234 | 0.08 | 307 | 1786 | 0.17 | 396 | 3774 | 0.10 | 220 | 1510 | 0.15 | 1127 | 9143 | 0.12 | 0.10 | 0.16 | 1.55 |
| *Ss-IgV_H_* | 2509 | 28445 | 0.09 | 1571 | 9215 | 0.17 | 2201 | 19629 | 0.11 | 1196 | 8333 | 0.14 | 6042 | 44363 | 0.14 | 0.11 | 0.16 | 1.40 |
| *Gc-IgV_H_* | 727 | 7407 | 0.10 | 578 | 3102 | 0.19 | 664 | 6579 | 0.10 | 569 | 3027 | 0.19 | 1284 | 14250 | 0.09 | 0.10 | 0.19 | 1.94 |
| *Xl-IgV_H_* | 88 | 902 | 0.10 | 50 | 292 | 0.17 | 67 | 611 | 0.11 | 33 | 252 | 0.13 | 192 | 1449 | 0.13 | 0.11 | 0.15 | 1.33 |
| *Gg-IgV_H_* | 1218 | 15455 | 0.08 | 995 | 5010 | 0.20 | 1391 | 10627 | 0.13 | 1011 | 5031 | 0.20 | 3903 | 24359 | 0.16 | 0.12 | 0.20 | 1.62 |
| *Mm-IgV_H_* | 3112 | 20493 | 0.15 | 1054 | 4209 | 0.25 | 689 | 11341 | 0.06 | 1730 | 13394 | 0.13 | 3907 | 25318 | 0.15 | 0.12 | 0.19 | 1.55 |
| *Hs-IgV_H_* | 3322 | 27855 | 0.12 | 1452 | 5900 | 0.25 | 932 | 15503 | 0.06 | 2424 | 19590 | 0.12 | 4328 | 38075 | 0.11 | 0.10 | 0.18 | 1.89 |
| *: The index was calculated as # AID hotspots/# nt. analysed.  Abbreviations: Ag: Arctic cod; Bs: Polar cod; Ma: Haddock; Ga: Silvery pout; Llo: Burbot; Pp: Forkbeard; Gm: Atlantic cod; Dr: zebrafish; Ss: Atlantic salmon; Ip: channel catfish; Tr: Japanese puffer fish; Gc: nurse shark; Xl: South African clawed toad; Gg: chicken; Mm: mouse; Hs: human. | | | | | | | | | | | | | | | | | | |

**Supplementary Table 4.** WGCW enrichment in complementarity determining regions (CDRs) *vs.* frameworks (FRs) of *IgV_H_* genes of various Gadidae and vertebrate species

|  | FR1 | | | CDR1 | | | FR2 | | | CDR2 | | | FR3 | | |  |  |  |
| --- | --- | --- | --- | --- | --- | --- | --- | --- | --- | --- | --- | --- | --- | --- | --- | --- | --- | --- |
|  | # AID hotspots | # nt. analyzed | Index* | # AID hotspots | # nt. analyzed | Index* | # AID hotspots | # nt. analyzed | Index* | # AID hotspots | # nt. analyzed | Index* | # AID hotspots | # nt. analyzed | Index* | Ave. FRs | Ave. CDRs | CDRs/FRs |
| *Ag-IgV_H_* | 170 | 5905 | 0.03 | 22 | 1328 | 0.02 | 38 | 1863 | 0.02 | 2 | 1968 | 0.00 | 228 | 7224 | 0.03 | 0.03 | 0.01 | 0.33 |
| *Bs-IgV_H_* | 83 | 2362 | 0.04 | 10 | 465 | 0.02 | 20 | 660 | 0.03 | 0 | 672 | 0.00 | 73 | 2201 | 0.03 | 0.03 | 0.01 | 0.33 |
| *Ma-IgV_H_* | 33 | 882 | 0.04 | 7 | 156 | 0.04 | 6 | 231 | 0.03 | 0 | 240 | 0.00 | 24 | 775 | 0.03 | 0.03 | 0.02 | 0.71 |
| *Ga-IgV_H_* | 43 | 1603 | 0.03 | 9 | 422 | 0.02 | 13 | 645 | 0.02 | 3 | 729 | 0.00 | 66 | 2407 | 0.03 | 0.02 | 0.01 | 0.51 |
| *Llo-IgV_H_* | 75 | 1935 | 0.04 | 14 | 357 | 0.04 | 6 | 495 | 0.01 | 4 | 528 | 0.01 | 51 | 1566 | 0.03 | 0.03 | 0.02 | 0.84 |
| *Pp-IgV_H_* | 82 | 2207 | 0.04 | 21 | 450 | 0.05 | 6 | 623 | 0.01 | 3 | 670 | 0.00 | 69 | 2042 | 0.03 | 0.03 | 0.03 | 0.95 |
| *Gadinae* | 435 | 18589 | 0.02 | 109 | 4744 | 0.02 | 165 | 9009 | 0.02 | 7 | 5598 | 0.00 | 765 | 25372 | 0.03 | 0.02 | 0.01 | 0.51 |
| *Gadidae* | 592 | 22731 | 0.03 | 144 | 5551 | 0.03 | 177 | 10127 | 0.02 | 14 | 6796 | 0.00 | 885 | 28980 | 0.03 | 0.02 | 0.01 | 0.57 |
| *Gm-IgV_H_* | 106 | 7837 | 0.01 | 61 | 2373 | 0.03 | 88 | 5610 | 0.02 | 2 | 1989 | 0.00 | 374 | 12765 | 0.03 | 0.02 | 0.01 | 0.68 |
| *Ip-IgV_H_* | 100 | 7199 | 0.01 | 95 | 2709 | 0.04 | 159 | 5498 | 0.03 | 72 | 2360 | 0.03 | 358 | 12381 | 0.03 | 0.02 | 0.03 | 1.37 |
| *Tr-IgV_H_* | 28 | 3675 | 0.01 | 79 | 1245 | 0.06 | 47 | 2361 | 0.02 | 58 | 1215 | 0.05 | 204 | 5517 | 0.04 | 0.02 | 0.06 | 2.59 |
| *Dr-IgV_H_* | 81 | 5234 | 0.02 | 75 | 1786 | 0.04 | 107 | 3774 | 0.03 | 34 | 1510 | 0.02 | 244 | 9143 | 0.03 | 0.02 | 0.03 | 1.37 |
| *Ss-IgV_H_* | 214 | 28445 | 0.01 | 269 | 9215 | 0.03 | 507 | 19629 | 0.03 | 207 | 8333 | 0.02 | 1289 | 44363 | 0.03 | 0.02 | 0.03 | 1.30 |
| *Gc-IgV_H_* | 5 | 7407 | 0.00 | 128 | 3102 | 0.04 | 50 | 6579 | 0.01 | 82 | 3027 | 0.03 | 70 | 14250 | 0.00 | 0.00 | 0.03 | 7.77 |
| *Xl-IgV_H_* | 10 | 902 | 0.01 | 12 | 292 | 0.04 | 8 | 611 | 0.01 | 7 | 252 | 0.03 | 39 | 1449 | 0.03 | 0.02 | 0.03 | 2.02 |
| *Gg-IgV_H_* | 226 | 15455 | 0.01 | 219 | 5010 | 0.04 | 149 | 10627 | 0.01 | 179 | 5031 | 0.04 | 740 | 24359 | 0.03 | 0.02 | 0.04 | 2.02 |
| *Mm-IgV_H_* | 841 | 20493 | 0.04 | 336 | 4209 | 0.08 | 219 | 11341 | 0.02 | 200 | 13394 | 0.01 | 1061 | 25318 | 0.04 | 0.03 | 0.05 | 1.39 |
| *Hs-IgV_H_* | 952 | 27855 | 0.03 | 355 | 5900 | 0.06 | 47 | 15503 | 0.00 | 316 | 19590 | 0.02 | 625 | 38075 | 0.02 | 0.02 | 0.04 | 2.13 |
| *: The index was calculated as # AID hotspots/# nt. analysed.  Abbreviations: Ag: Arctic cod; Bs: Polar cod; Ma: Haddock; Ga: Silvery pout; Llo: Burbot; Pp: Forkbeard; Gm: Atlantic cod; Dr: zebrafish; Ss: Atlantic salmon; Ip: channel catfish; Tr: Japanese puffer fish; Gc: nurse shark; Xl: South African clawed toad; Gg: chicken; Mm: mouse; Hs: human. | | | | | | | | | | | | | | | | | | |

**Supplementary Table 5.** AID hotspot abundance in the entire *IgV_H_* genes and GC content of annotated complete protein coding genes (CDSs) of various Gadidae and vertebrate species

|  | *IgV_H_* gene analysis | | | | | | Genomic analysis | |
| --- | --- | --- | --- | --- | --- | --- | --- | --- |
|  | # transcripts | # nt. analyzed | WRC/GYW | | WGCW | |  |  |
|  |  |  | # motifs | Index* | # motifs | Index* | # CDSs | GC% |
| *Ag-IgV_H_* | 87 | 18288 | 2571 | 0.1406 | 460 | 0.0252 | 8 | 60.35 |
| *Bs-IgV_H_* | 20 | 6360 | 979 | 0.1539 | 186 | 0.0292 | 73 | 61.66 |
| *Ma-IgV_H_* | 7 | 2284 | 374 | 0.1637 | 70 | 0.0306 | 44 | 54.80 |
| *Ga-IgV_H_* | 25 | 5806 | 806 | 0.1388 | 134 | 0.0231 | 6 | 60.87 |
| *Llo-IgV_H_* | 15 | 4881 | 755 | 0.1547 | 150 | 0.0307 | 32 | 59.19 |
| *Pp-IgV_H_* | 19 | 5992 | 936 | 0.1562 | 181 | 0.0302 | 9 | 60.23 |
| *Gadinae* | 251 | 63312 | 8447 | 0.1334 | 1481 | 0.0234 | NA | 59.44 |
| *Gadidae* | 285 | 74185 | 10138 | 0.1367 | 1812 | 0.0244 | NA | 59.52 |
| *Gm-IgV_H_* | 112 | 30574 | 3717 | 0.1216 | 631 | 0.0206 | 44330 | 59.53 |
| *Ip-IgV_H_* | 109 | 30147 | 3928 | 0.1303 | 784 | 0.0260 | 47956 | 51.46 |
| *Tr-IgV_H_* | 49 | 14013 | 1782 | 0.1272 | 416 | 0.0297 | 46294 | 54.11 |
| *Dr-IgV_H_* | 76 | 21447 | 2460 | 0.1147 | 541 | 0.0252 | 57060 | 49.85 |
| *Ss-IgV_H_* | 405 | 109985 | 13519 | 0.1229 | 2486 | 0.0226 | 97576 | 55.12 |
| *Gc-IgV_H_* | 129 | 34365 | 3822 | 0.1112 | 335 | 0.0097 | 1507 | 47.97 |
| *Xl-IgV_H_* | 44 | 3506 | 430 | 0.1226 | 76 | 0.0217 | 49356 | 45.62 |
| *Gg-IgV_H_* | 239 | 60482 | 8518 | 0.1408 | 1513 | 0.0250 | 56680 | 50.23 |
| *Mm-IgV_H_* | 420 | 74755 | 10492 | 0.1404 | 2657 | 0.0355 | 88579 | 51.96 |
| *Hs-IgV_H_* | 727 | 106923 | 12458 | 0.1165 | 2295 | 0.0215 | 120426 | 51.02 |
| *: The index was calculated as # AID hotspots/# nt. analysed.  NA: Since the number of analyzed *IgV_H_* transcripts was very low for Gadidae species other than Atlantic cod, we decided not to report this parameter for Gadidae and Gadinae groups.  Abbreviations: Ag: Arctic cod; Bs: Polar cod; Ma: Haddock; Ga: Silvery pout; Llo: Burbot; Pp: Forkbeard; Gm: Atlantic cod; Dr: zebrafish; Ss: Atlantic salmon; Ip: channel catfish; Tr: Japanese puffer fish; Gc: nurse shark; Xl: South African clawed toad; Gg: chicken; Mm: mouse; Hs: human*.* | | | | | | | | |

Supplementary Table 6. The sequence of primers used in this study

| Gene | | Direction | Primer sequence (5' to 3') | Amplification efficiency (%) | R^2^ | Amplicon size (bp) | Application |
| --- | --- | --- | --- | --- | --- | --- | --- |
| *Activation induced cytidine deaminase (aicda)*; *Gm-aicda* | Set 1 | Forward | TAGTAAGCTAGACAGTGTGCTCTTGG | NA | NA | 608 | Detecting *Gm-aicda* ORF |
|  |  | Reverse | CATCTCTTAAATCTTCTGTTTCACATGG |  |  |  |  |
|  | Set 2 | Forward | CTCTGCTTCGTAGTAAAGAGAAGGC | NA | NA | 473 |  |
|  |  | Reverse | AGTTTTCTTGACAGACGCACATAATTGG |  |  |  |  |
| *Gm-aicda* | First PCR | Forward | GACTTCGGACACCTACGCAATCGCACTGGC | NA | NA | NA | 3' RACE-PCR |
|  |  | Reverse | CCTCAGGTCCCTCAAGCCCTCTACATGCGG |  |  |  | 5' RACE-PCR |
|  | Nested PCR | Forward | CGCAATCGCACTGGCTGCCACGCAGAGCTG | NA | NA | NA | 3' RACE-PCR |
|  |  | Reverse | GCCCTCTACATGCGGACTGCCCTCCAGGTC |  |  |  | 5' RACE-PCR |
| *Gm-aicda* | First PCR | Forward | GACTTTCAAAATGATTAGTAAGCTAGACAG | NA | NA | 780^ⅰ^ | Confirming *Gm-aicda* isoforms |
| *T-Gm-aicda* |  | Forward | GAATGGTTGATGATTACAGACCC |  |  | 822 |  |
| *Gm-aicda* -3'UTR-r1 |  | Reverse | TTGGACTACATAGGCGGTTTCAC |  |  |  |  |
| *Gm-aicda* | Nested PCR | Forward | TAAGCTAGACAGTGTGCTCTTGG | NA | NA | 747^ⅱ^ |  |
| *T-Gm-aicda* |  | Forward | GATTACAGACCCTTACCGCAG |  |  | 799 |  |
| *Gm-aicda*-3'UTR-r1 |  | Reverse | GGTTTCACAAAGTTCTACAGTTTGC |  |  |  |  |
| *Eukaryotic translation elongation factor 1 alpha* (*ef1-α*)^ⅲ^ | | Forward | CCCTCCAGGACGTCTACAAG | NA | NA | 150 | Tissue and developmental panel (normalizer) |
|  |  | Reverse | GAGACTCGTGGTGCATCTCA |  |  |  |  |
| *Gm-aicda* | | Forward | AGTAAGCTAGACAGTGTGCTC | 101.57 | 0.989 | 125 | Tissue and developmental panel; qPCR |
|  |  | Reverse | CAGGTCCAAGCCTTCTCTT |  |  |  |  |
| *T-Gm-aicda* | | Forward | TTCTCTCCTATGTCTCAGTGTGC | 100.47 | 0.989 | 133 |  |
|  |  | Reverse | GGAATCAGGTCCAAGCCTTC |  |  |  |  |
| *60S acidic ribosomal protein P1* (*rplp1*)^ⅲ^ | | Forward | TCTGAAGCTAAGGCCCTCAA | 104.8 | 0.998 | 141 | qPCR (normalizers) |
|  |  | Reverse | ATCGTCGTGGAGGATCAGAG |  |  |  |  |
| *ATP synthase H+ transporting, mitochondrial Fo complex, subunit F2* (*atps*)^ⅳ^ | | Forward | ACATGGATAAATGGCTTTTTGC | 99.43 | 0.994 | 155 |  |
|  |  | Reverse | TTGAAGAAGTAGTGTGGCTGGA |  |  |  |  |
| Deamination-specific primers^v^ | First PCR | Forward | GGGATATAGGGGTTTTTTGAGGTTTGGTATTATTTAAAT | NA | NA | 548 | PCR-based AID activity assay |
|  |  | Reverse | ACACAACCAACTTTCATTCCAACCACAAACTTTCAATA | NA | NA |  |  |
|  | Nested PCR | Forward | CTTATCTTGGTTCTGTGGCAACCGACTGCCTGCTAACAGG | NA | NA | 442 |  |
|  |  | Reverse | CCAACTTTCATTCCAACCACAAACTTTCAATAAATT | NA | NA |  |  |
| ^ⅰ^: If used with *Gm-aicda*-3'UTR-r1  ^ⅱ^: If used with *Gm-aicda*-3'UTR-r2  ^ⅲ^: The primer sequences for these genes were previously published in Inkpen *et al.*, (2015)  ^ⅳ^: The primer sequences for these genes were previously published in Hori *et al.,* (2012)  ^v^: The primer sequences for this gene are modified to specifically amplify heavily C-to-U-mutated sequence | | | | | | | |

Supplementary Table 7. GenBank accession number of the *teleost aicda and* Ig genes used in this *study*

| **Aicda sequences** | | | |
| --- | --- | --- | --- |
| Acession ID | Description | Species | |
| OP856785 | The Atlantic cod full AID cDNA | *Gadus Morhua* | |
| PRJEB12469 | AID genes used for the expression of extant enzymes and for ASR (sequences  Listed in t | 66 teleost species | |
| **Protein queries (full-length IgM and IgD):** | | | |
| Accession ID | Description | | Species |
| ACO88906.1 | IgD | | *Siniperca chuatsi* |
| BAD34541.1 | IgD | | *Takifugu rubripes* |
| AIC33830.1 | IgD | | *Lutjanus sanguineus* |
| AFI33218.1 | IgD | | *Epinephelus coioides* |
| AAX78205.1 | IgM | | *Epinephelus coioides* |
| BAB60868.1 | IgM | | *Paralichthys olivaceus* |
| A0A126CRL5 | IgM | | *Oreochromis niloticus* |
| A0A0G3VMZ6 | IgM | | *Gadus macrocephalus* |
| **Full-length IgZ gene:** | | | |
| ID | Description | | Species |
| AIC33829.1 | IgZ heavy chain transmembrane | | *Lutjanus sanguineus* |
| AIC33828.1 | immunoglobulin Z heavy chain | | *Lutjanus sanguineus* |
| ADD82653.1 | immunoglobulin Z heavy chain, partial | | *Ctenopharyngodon idella* |
| ADD82655.1 | secretory IgZ | | *Ctenopharyngodon idella* |
| ABY76180.1 | membrane bound IgZ, partial | | *Ctenopharyngodon idella* |
| **IgV_H_ genes:** | | | |
| ID | Description | | Species |
| AJ274705.1 | partial mRNA for immunoglobulin heavy chain variable region clone 0997031136 (0936) Family I | | *Gadus morhua* |
| AJ274706.1 | partial mRNA for immunoglobulin heavy chain variable region clone 1297030733 (1233) Family I | | *Gadus morhua* |
| AJ274707.1 | partial mRNA for immunoglobulin heavy chain variable region clone 1297030741 (1241) Family I | | *Gadus morhua* |
| AJ274708.1 | partial mRNA for immunoglobulin heavy chain variable region clone 1997102105 (1905) Family II | | *Gadus morhua* |
| AJ274709.1 | partial mRNA for immunoglobulin heavy chain variable region clone 1997102107 (1907a) Family II | | *Gadus morhua* |
| AJ274710.1 | partial mRNA for immunoglobulin heavy chain variable region clone 1997111806 (1906) Family II | | *Gadus morhua* |
| AJ274711.1 | partial mRNA for immunoglobulin heavy chain variable region clone 0997031139 (1139) Family IV | | *Gadus morhua* |
| AJ274712.1 | partial mRNA for immunoglobulin heavy chain variable region clone 1297021302 (1202a) Family IV | | *Gadus morhua* |
| AJ274713.1 | partial mRNA for immunoglobulin heavy chain variable region clone 1297021409 (1209) Family IV | | *Gadus morhua* |
| AJ274714.1 | partial mRNA for immunoglobulin heavy chain variable region clone 1297030705 (1205b) Family IV | | *Gadus morhua* |
| AJ274715.1 | partial mRNA for immunoglobulin heavy chain variable region clone 1297021305 (1205a) Family III | | *Gadus morhua* |
| AJ274716.1 | partial mRNA for immunoglobulin heavy chain variable region clone 0997021408 (0908) Family III | | *Gadus morhua* |
| AJ274717.1 | partial mRNA for immunoglobulin heavy chain variable region clone 1297021402 (1202b) Family III | | *Gadus morhua* |
| AJ274718.1 | partial mRNA for immunoglobulin heavy chain variable region clone 1297021408 (1208) | | *Gadus morhua* |
| AJ274719.1 | partial mRNA for immunoglobulin heavy chain variable region clone 1297021411 (1211) Family III | | *Gadus morhua* |
| AJ274720.1 | partial mRNA for immunoglobulin heavy chain variable region clone 1297030702 (1202c) Family III | | *Gadus morhua* |
| AJ274721.1 | partial mRNA for immunoglobulin heavy chain variable region clone 1297030715 (1215) family III | | *Gadus morhua* |
| AJ274722.1 | partial mRNA for immunoglobulin heavy chain variable region clone 1297030722 (1222) Family III | | *Gadus morhua* |
| AJ274723.1 | partial mRNA for immunoglobulin heavy chain variable region clone 2096110714 (2014) Family III | | *Gadus morhua* |
| AJ274724.1 | partial mRNA for immunoglobulin heavy chain variable region clone 2096110629 (2029) Family III | | *Gadus morhua* |
| AJ274725.1 | partial mRNA for immunoglobulin heavy chain variable region clone 2096110631 (2031) Family III | | *Gadus morhua* |
| AJ274726.1 | partial mRNA for immunoglobulin heavy chain variable region clone 0997021401 (0901) Family III | | *Gadus morhua* |
| AJ274727.1 | partial mRNA for immunoglobulin heavy chain variable region clone 0997031130 (0930) Family III | | *Gadus morhua* |
| AJ274728.1 | partial mRNA for immunoglobulin heavy chain variable region clone 1998012302 (1902) Family III | | *Gadus morhua* |
| AJ274729.1 | partial mRNA for immunoglobulin heavy chain variable region clone 1297030732 (1232) Family III | | *Gadus morhua* |
| AJ274730.1 | partial mRNA for immunoglobulin heavy chain variable region clone 1297030745 (1245) Family III | | *Gadus morhua* |
| AJ274731.1 | partial mRNA for immunoglobulin heavy chain variable region clone 0997031129 (0929) Family III | | *Gadus morhua* |
| AJ274732.1 | partial mRNA for immunoglobulin heavy chain variable region clone 1297021304 (1204) Family III | | *Gadus morhua* |
| AJ274733.1 | partial mRNA for immunoglobulin heavy chain variable region clone 1297030714 (1214) Family III | | *Gadus morhua* |
| AJ274734.1 | partial mRNA for immunoglobulin heavy chain variable region clone 1997111807 (1907b) Family III | | *Gadus morhua* |
| AJ274735.1 | partial mRNA for immunoglobulin heavy chain variable region clone 0997031143 (0943) Family III | | *Gadus morhua* |
| AJ274736.1 | partial mRNA for immunoglobulin heavy chain variable region clone 0997031127 (0927) Family III | | *Gadus morhua* |
| AJ274737.1 | partial mRNA for immunoglobulin heavy chain variable region clone 1297030703 (1203b) family III | | *Gadus morhua* |
| AJ274738.1 | partial mRNA for immunoglobulin heavy chain variable region clone 1997102101 (1901) Family III | | *Gadus morhua* |
| AJ274739.1 | partial mRNA for immunoglobulin heavy chain variable region clone 1498012214 (1414) Family III | | *Gadus morhua* |
| AJ274740.1 | partial mRNA for immunoglobulin heavy chain variable region clone 0997031134 (0934) Family III | | *Gadus morhua* |
| AJ274741.1 | partial mRNA for immunoglobulin heavy chain variable region clone 2098012010 (2010) Family III | | *Gadus morhua* |
| AJ274742.1 | partial mRNA for immunoglobulin heavy chain variable region clone 0997031138 (0938) Family III | | *Gadus morhua* |
| AJ274743.1 | partial mRNA for immunoglobulin heavy chain variable region clone 0997021404 (0904) Family III | | *Gadus morhua* |
| AJ274744.1 | partial mRNA for immunoglobulin heavy chain variable region clone 1297030706 (1206) Family III | | *Gadus morhua* |
| AJ274745.1 | partial mRNA for immunoglobulin heavy chain variable region clone 1497103004 (1404) Family III | | *Gadus morhua* |
| AJ274746.1 | partial mRNA for immunoglobulin heavy chain variable region clone 1297030728 (1228) | | *Gadus morhua* |
| AJ274747.1 | partial mRNA for immunoglobulin heavy chain variable region clone 1297021303 (1203a) Family III | | *Gadus morhua* |
| AJ274748.1 | partial mRNA for immunoglobulin heavy chain variable region clone 2098011603 (2003) Family III | | *Gadus morhua* |
| AJ274749.1 | partial mRNA for immunoglobulin heavy chain variable region clone 1297030710 (1210b) Family III | | *Gadus morhua* |
| AJ274750.1 | partial mRNA for immunoglobulin heavy chain variable region clone 1297030719 (1219) Family III | | *Gadus morhua* |
| AJ274751.1 | partial mRNA for immunoglobulin heavy chain variable region clone 0997021407 (0907) Family III | | *Gadus morhua* |
| AJ274752.1 | partial mRNA for immunoglobulin heavy chain variable region clone 1297021310 (1210a) Family III | | *Gadus morhua* |
| AJ274753.1 | partial mRNA for immunoglobulin heavy chain variable region clone 0997031140 (0940) Family III | | *Gadus morhua* |
| AJ274754.1 | partial mRNA for immunoglobulin heavy chain variable region clone 1498020906 (1406) Family III | | *Gadus morhua* |
| AJ274755.1 | partial mRNA for immunoglobulin heavy chain variable region clone 2096110626 (2026) Family III | | *Gadus morhua* |
| AJ274756.1 | partial mRNA for immunoglobulin heavy chain variable region clone 2096102205 (2005) family III | | *Gadus morhua* |
| AJ279353.1 | partial mRNA for immunoglobulin heavy chain variable region clone 21 | | *Gadus morhua* |
| AJ279354.1 | partial mRNA for immunoglobulin heavy chain variable region clone 34 | | *Gadus morhua* |
| AJ279355.1 | partial mRNA for immunoglobulin heavy chain variable region clone 40 | | *Gadus morhua* |
| AJ279356.1 | partial mRNA for immunoglobulin heavy chain variable region clone 49 | | *Gadus morhua* |
| AJ279357.1 | partial mRNA for immunoglobulin heavy chain variable region clone 2 | | *Gadus morhua* |
| AJ279358.1 | partial mRNA for immunoglobulin heavy chain variable region clone 14 | | *Gadus morhua* |
| AJ279359.1 | partial mRNA for immunoglobulin heavy chain variable region clone 15 | | *Gadus morhua* |
| AJ279360.1 | partial mRNA for immunoglobulin heavy chain variable region clone 29 | | *Gadus morhua* |
| AJ279361.1 | partial mRNA for immunoglobulin heavy chain variable region clone 44 | | *Gadus morhua* |
| AJ279362.1 | partial mRNA for immunoglobulin heavy chain variable region clone 19 | | *Gadus morhua* |
| AJ279363.1 | partial mRNA for immunoglobulin heavy chain variable region clone 38 | | *Gadus morhua* |
| AJ279365.1 | partial mRNA for immunoglobulin heavy chain variable region clone 39 | | *Gadus morhua* |
| AJ279366.1 | partial mRNA for immunoglobulin heavy chain variable region clone 31 | | *Gadus morhua* |
| AJ279367.1 | partial mRNA for immunoglobulin heavy chain variable region clone 25 | | *Gadus morhua* |
| AJ279368.1 | partial mRNA for immunoglobulin heavy chain variable region clone 23 | | *Gadus morhua* |
| AJ279369.1 | partial mRNA for immunoglobulin heavy chain variable region clone 263 | | *Gadus morhua* |
| AJ279370.1 | partial mRNA for immunoglobulin heavy chain variable region clone 35 | | *Gadus morhua* |
| AJ279371.1 | partial mRNA for immunoglobulin heavy chain variable region clone 33 | | *Gadus morhua* |
| AJ279372.1 | partial mRNA for immunoglobulin heavy chain variable region clone 98 | | *Gadus morhua* |
| AJ279373.1 | partial mRNA for immunoglobulin heavy chain variable region clone 9 | | *Gadus morhua* |
| AJ279374.1 | partial mRNA for immunoglobulin heavy chain variable region clone 28 | | *Gadus morhua* |
| AJ279375.1 | partial mRNA for immunoglobulin heavy chain variable region clone 127 | | *Gadus morhua* |
| AJ279376.1 | partial mRNA for immunoglobulin heavy chain variable region clone 4 | | *Gadus morhua* |
| AJ279377.1 | partial mRNA for immunoglobulin heavy chain variable region clone 11 | | *Gadus morhua* |
| AJ279378.1 | partial mRNA for immunoglobulin heavy chain variable region clone 22 | | *Gadus morhua* |
| AJ279380.1 | partial mRNA for immunoglobulin heavy chain variable region clone 264 | | *Gadus morhua* |
| AJ279381.1 | partial mRNA for immunoglobulin heavy chain variable region clone 48 | | *Gadus morhua* |
| AJ279382.1 | partial mRNA for immunoglobulin heavy chain variable region clone 109 | | *Gadus morhua* |
| AJ279383.1 | partial mRNA for immunoglobulin heavy chain variable region clone 110 | | *Gadus morhua* |
| AJ279384.1 | partial mRNA for immunoglobulin heavy chain variable region clone 45 | | *Gadus morhua* |
| AJ279385.1 | partial mRNA for immunoglobulin heavy chain variable region clone 32 | | *Gadus morhua* |
| AJ279386.1 | partial mRNA for immunoglobulin heavy chain variable region clone 244 | | *Gadus morhua* |
| AJ279387.1 | partial mRNA for immunoglobulin heavy chain variable region clone 12 | | *Gadus morhua* |
| AJ279388.1 | partial mRNA for immunoglobulin heavy chain variable region clone 20 | | *Gadus morhua* |
| AJ279389.1 | partial mRNA for immunoglobulin heavy chain variable region clone 1 | | *Gadus morhua* |
| AJ279390.1 | partial mRNA for immunoglobulin heavy chain variable region clone 30 | | *Gadus morhua* |
| AJ279391.1 | partial mRNA for immunoglobulin heavy chain variable region clone 36 | | *Gadus morhua* |
| AJ279392.1 | partial mRNA for immunoglobulin heavy chain variable region clone 82 | | *Gadus morhua* |
| AJ279393.1 | partial mRNA for immunoglobulin heavy chain variable region clone 42 | | *Gadus morhua* |
| AJ279394.1 | partial mRNA for immunoglobulin heavy chain variable region clone 8 | | *Gadus morhua* |
| AJ279395.1 | partial mRNA for immunoglobulin heavy chain variable region clone 46 | | *Gadus morhua* |
| AJ279396.1 | partial mRNA for immunoglobulin heavy chain variable region clone 17 | | *Gadus morhua* |
| AJ279397.1 | partial mRNA for immunoglobulin heavy chain variable region clone 90 | | *Gadus morhua* |
| DQ230541.1 | clone 1B07AVH1 CS3 immunoglobulin heavy chain variable region mRNA, partial cds | | *Ictalurus punctatus* |
| DQ230547.1 | clone 3B11AVH1 immunoglobulin heavy chain variable region mRNA, partial cds | | *Ictalurus punctatus* |
| DQ230550.1 | clone 3D04AVH1 CS1 immunoglobulin heavy chain variable region mRNA, partial cds | | *Ictalurus punctatus* |
| DQ230551.1 | clone 3D08AVH1 CS4 immunoglobulin heavy chain variable region mRNA, partial cds | | *Ictalurus punctatus* |
| DQ230552.1 | clone 3E09AVH1 immunoglobulin heavy chain variable region mRNA, partial cds | | *Ictalurus punctatus* |
| DQ230553.1 | clone 3F02AVH1 CS3 immunoglobulin heavy chain variable region mRNA, partial cds | | *Ictalurus punctatus* |
| DQ230555.1 | clone 3G07AVH1 immunoglobulin heavy chain variable region mRNA, partial cds | | *Ictalurus punctatus* |
| DQ230557.1 | clone 3G12AVH1 immunoglobulin heavy chain variable region mRNA, partial cds | | *Ictalurus punctatus* |
| DQ230558.1 | clone 6E04AVH1 immunoglobulin heavy chain variable region mRNA, partial cds | | *Ictalurus punctatus* |
| DQ230560.1 | clone 6G04AVH1 immunoglobulin heavy chain variable region mRNA, partial cds | | *Ictalurus punctatus* |
| DQ230562.1 | clone 6H05AVH1 CS4 immunoglobulin heavy chain variable region mRNA, partial cds | | *Ictalurus punctatus* |
| AY238358.1 | immunoglobulin heavy chain variable region mRNA, partial cds | | *Ictalurus punctatus* |
| EU492547.1 | clone 15B02VH1PBL immunoglobulin heavy chain variable region mRNA, partial cds | | *Ictalurus punctatus* |
| EU492548.1 | clone 15B03VH1PBL immunoglobulin heavy chain variable region mRNA, partial cds | | *Ictalurus punctatus* |
| EU492549.1 | clone 15B04VH1PBL immunoglobulin heavy chain variable region mRNA, partial cds | | *Ictalurus punctatus* |
| EU492550.1 | clone 15B05VH1PBL immunoglobulin heavy chain variable region mRNA, partial cds | | *Ictalurus punctatus* |
| EU492551.1 | clone 15B06VH1PBL immunoglobulin heavy chain variable region mRNA, partial cds | | *Ictalurus punctatus* |
| EU492558.1 | clone 15C08VH1PBL immunoglobulin heavy chain variable region mRNA, partial cds | | *Ictalurus punctatus* |
| EU492557.1 | clone 15C07VH1PBL immunoglobulin heavy chain variable region mRNA, partial cds | | *Ictalurus punctatus* |
| EU492554.1 | clone 15B12VH1PBL immunoglobulin heavy chain variable region mRNA, partial cds | | *Ictalurus punctatus* |
| EU492555.1 | clone 15C02VH1PBL immunoglobulin heavy chain variable region mRNA, partial cds | | *Ictalurus punctatus* |
| EU492553.1 | clone 15B10VH1PBL immunoglobulin heavy chain variable region mRNA, partial cds | | *Ictalurus punctatus* |
| EU492587.1 | clone 19D12VH1PBL CS2 immunoglobulin heavy chain variable region mRNA, partial cds | | *Ictalurus punctatus* |
| EU492591.1 | clone 15D01VH1AK immunoglobulin heavy chain variable region mRNA, partial cds | | *Ictalurus punctatus* |
| EU492596.1 | clone 15D10VH1AK immunoglobulin heavy chain variable region mRNA, partial cds | | *Ictalurus punctatus* |
| EU492595.1 | clone 15D07VH1AK immunoglobulin heavy chain variable region mRNA, partial cds | | *Ictalurus punctatus* |
| EU492597.1 | clone 15D11VH1AK immunoglobulin heavy chain variable region mRNA, partial cds | | *Ictalurus punctatus* |
| EU492590.1 | clone 15C11VH1AK immunoglobulin heavy chain variable region mRNA, partial cds | | *Ictalurus punctatus* |
| EU492598.1 | clone 15D12VH1AK immunoglobulin heavy chain variable region mRNA, partial cds | | *Ictalurus punctatus* |
| EU492594.1 | clone 15D06VH1AK immunoglobulin heavy chain variable region mRNA, partial cds | | *Ictalurus punctatus* |
| EU492592.1 | clone 15D02VH1AK immunoglobulin heavy chain variable region mRNA, partial cds | | *Ictalurus punctatus* |
| EU492637.1 | clone 15E01VH1SP immunoglobulin heavy chain variable region mRNA, partial cds | | *Ictalurus punctatus* |
| EU492642.1 | clone 15E07VH1SP immunoglobulin heavy chain variable region mRNA, partial cds | | *Ictalurus punctatus* |
| EU492643.1 | clone 15E09VH1SP immunoglobulin heavy chain variable region mRNA, partial cds | | *Ictalurus punctatus* |
| EU492641.1 | clone 15E06VH1SP immunoglobulin heavy chain variable region mRNA, partial cds | | *Ictalurus punctatus* |
| EU492695.1 | clone 15A01VH1GL immunoglobulin heavy chain variable region mRNA, partial cds | | *Ictalurus punctatus* |
| EU492696.1 | clone 15A02VH1GL immunoglobulin heavy chain variable region mRNA, partial cds | | *Ictalurus punctatus* |
| EU492697.1 | clone 15A04VH1GL CS3 immunoglobulin heavy chain variable region mRNA, partial cds | | *Ictalurus punctatus* |
| EU492698.1 | clone 15A06VH1GL immunoglobulin heavy chain variable region mRNA, partial cds | | *Ictalurus punctatus* |
| EU492699.1 | clone 15A07VH1GL immunoglobulin heavy chain variable region mRNA, partial cds | | *Ictalurus punctatus* |
| EU492700.1 | clone 15A08VH1GL CS1 immunoglobulin heavy chain variable region mRNA, partial cds | | *Ictalurus punctatus* |
| EU492701.1 | clone 15A10VH1GL CS3 immunoglobulin heavy chain variable region mRNA, partial cds | | *Ictalurus punctatus* |
| EU492702.1 | clone 15A11VH1GL immunoglobulin heavy chain variable region mRNA, partial cds | | *Ictalurus punctatus* |
| EU492733.1 | clone 18D12VH1GL CS1 immunoglobulin heavy chain variable region mRNA, partial cds | | *Ictalurus punctatus* |
| EU492763.1 | clone 18C11VH1SK CS1 immunoglobulin heavy chain variable region mRNA, partial cds | |  |
| EU492734.1 | clone 18E01VH1GL CS1 immunoglobulin heavy chain variable region mRNA, partial cds | | *Ictalurus punctatus* |
| EU492746.1 | clone 18C12VH1SK immunoglobulin heavy chain variable region mRNA, partial cds | | *Ictalurus punctatus* |
| EU492747.1 | clone 3F02AVH1 CS3 immunoglobulin heavy chain variable region mRNA, partial cds | | *Ictalurus punctatus* |
| EU492750.1 | clone 19B04VH1SK CS1 immunoglobulin heavy chain variable region mRNA, partial cds | | *Ictalurus punctatus* |
| EU492751.1 | clone 19B09VH1SK CS1 immunoglobulin heavy chain variable region mRNA, partial cds | | *Ictalurus punctatus* |
| EU492835.1 | clone 19G12VH1I3 CS2 immunoglobulin heavy chain variable region mRNA, partial cds | | *Ictalurus punctatus* |
| EU492837.1 | clone 20B06VH1I3 CS2 immunoglobulin heavy chain variable region mRNA, partial cds | | *Ictalurus punctatus* |
| EU492838.1 | clone 20B12VH1I3 CS2 immunoglobulin heavy chain variable region mRNA, partial cds | | *Ictalurus punctatus* |
| EU492840.1 | clone 20F07VH1I3 CS6 immunoglobulin heavy chain variable region mRNA, partial cds | | *Ictalurus punctatus* |
| EU492845.1 | clone 21D11VH1I3 immunoglobulin heavy chain variable region mRNA, partial cds | | *Ictalurus punctatus* |
| EU492851.1 | clone 21H11VH1I3 immunoglobulin heavy chain variable region mRNA, partial cds | | *Ictalurus punctatus* |
| EU492850.1 | clone 21H10VH1I3 immunoglobulin heavy chain variable region mRNA, partial cds | | *Ictalurus punctatus* |
| EU492844.1 | clone 21D08VH1I3 immunoglobulin heavy chain variable region mRNA, partial cds | | *Ictalurus punctatus* |
| EU492842.1 | clone 21D06VH1I3 immunoglobulin heavy chain variable region mRNA, partial cds | | *Ictalurus punctatus* |
| EU492795.1 | clone 19A07VH1I2 CS1 immunoglobulin heavy chain variable region mRNA, partial cds | | *Ictalurus punctatus* |
| EU492796.1 | clone 19A10VH1I2 CS1 immunoglobulin heavy chain variable region mRNA, partial cds | | *Ictalurus punctatus* |
| EU492797.1 | clone 19A11VH1I2 CS1 immunoglobulin heavy chain variable region mRNA, partial cds | | *Ictalurus punctatus* |
| EU492802.1 | clone 20A01VH1I2 CS1 immunoglobulin heavy chain variable region mRNA, partial cds | | *Ictalurus punctatus* |
| EU492805.1 | clone 21C10VH1I2 immunoglobulin heavy chain variable region mRNA, partial cds | | *Ictalurus punctatus* |
| EU492806.1 | clone 21C11VH1I2 immunoglobulin heavy chain variable region mRNA, partial cds | | *Ictalurus punctatus* |
| EU492807.1 | clone 21D01VH1I2 immunoglobulin heavy chain variable region mRNA, partial cds | | *Ictalurus punctatus* |
| EU492808.1 | clone 21D03VH1I2 immunoglobulin heavy chain variable region mRNA, partial cds | | *Ictalurus punctatus* |
| EU492809.1 | clone 21D04VH1I2 immunoglobulin heavy chain variable region mRNA, partial cds | | *Ictalurus punctatus* |
| EU492813.1 | clone 19E02RevI2 CS2 immunoglobulin heavy chain variable region mRNA, partial cds | | *Ictalurus punctatus* |
| EU492816.1 | clone 15G05VH1I3 CS2 immunoglobulin heavy chain variable region mRNA, partial cds | | *Ictalurus punctatus* |
| EU492818.1 | clone 15H07VH1I3 CS2 immunoglobulin heavy chain variable region mRNA, partial cds | | *Ictalurus punctatus* |
| EU492819.1 | clone 15H11VH1I3 CS2 immunoglobulin heavy chain variable region mRNA, partial cds | | *Ictalurus punctatus* |
| EU492820.1 | clone 15H12VH1I3 CS2 immunoglobulin heavy chain variable region mRNA, partial cds | | *Ictalurus punctatus* |
| EU492843.1 | clone 21D07VH1I3 CS6 immunoglobulin heavy chain variable region mRNA, partial cds | | *Ictalurus punctatus* |
| EU492752.1 | clone 19B10VH1SK CS1 immunoglobulin heavy chain variable region mRNA, partial cds | | *Ictalurus punctatus* |
| EU492754.1 | clone 20A06VH1SK CS1 immunoglobulin heavy chain variable region mRNA, partial cds | | *Ictalurus punctatus* |
| EU492755.1 | clone 20A07VH1SK CS1 immunoglobulin heavy chain variable region mRNA, partial cds | | *Ictalurus punctatus* |
| DQ230542.1 | clone 1B10AVH1 CS2 immunoglobulin heavy chain variable region mRNA, partial cds | | *Ictalurus punctatus* |
| DQ230544.1 | clone 1F07AVH1 immunoglobulin heavy chain variable region mRNA, partial cds | | *Ictalurus punctatus* |
| DQ230548.1 | clone 3C11AVH1 immunoglobulin heavy chain variable region mRNA, partial cds | | *Ictalurus punctatus* |
| GU296460.1 | clone 5r21 immunoglobulin delta heavy chain membrane bound form mRNA, partial cds | | *Ictalurus punctatus* |
| EU492836.1 | clone 19H09VH1I3 immunoglobulin heavy chain variable region mRNA, partial cds | | *Ictalurus punctatus* |
| EU492834.1 | clone 19C10VH1I3 CS2 immunoglobulin heavy chain variable region mRNA, partial cds | | *Ictalurus punctatus* |
| EU492794.1 | clone 19A02VH1I2 CS1 immunoglobulin heavy chain variable region mRNA, partial cds | | *Ictalurus punctatus* |
| DQ230556.1 | clone 3G11AVH1 immunoglobulin heavy chain variable region mRNA, partial cds | | *Ictalurus punctatus* |
| EU492552.1 | clone 15B07VH1PBL immunoglobulin heavy chain variable region mRNA, partial cds | | *Ictalurus punctatus* |
| EU492640.1 | clone 15E05VH1SP immunoglobulin heavy chain variable region mRNA, partial cds | | *Ictalurus punctatus* |
| EU492761.1 | clone 21C07VH1SK CS1 immunoglobulin heavy chain variable region mRNA, partial cds | | *Ictalurus punctatus* |
| EU492759.1 | clone 21C05VH1SK CS1 immunoglobulin heavy chain variable region mRNA, partial cds | | *Ictalurus punctatus* |
| EU492762.1 | clone 21H01VH1SK CS1 immunoglobulin heavy chain variable region mRNA, partial cds | | *Ictalurus punctatus* |
| EU492815.1 | clone 15F08VH1I2 CS1 immunoglobulin heavy chain variable region mRNA, partial cds | | *Ictalurus punctatus* |
| EU492756.1 | clone 20A10VH1SK CS1 immunoglobulin heavy chain variable region mRNA, partial cds | | *Ictalurus punctatus* |
| EU492757.1 | clone 20A11VH1SK CS1 immunoglobulin heavy chain variable region mRNA, partial cds | | *Ictalurus punctatus* |
| EU492764.1 | clone 15F02VH1I2 immunoglobulin heavy chain variable region mRNA, partial cds | | *Ictalurus punctatus* |
| EU492765.1 | clone 15F06VH1I2 CS1 immunoglobulin heavy chain variable region mRNA, partial cds | | *Ictalurus punctatus* |
| EU492766.1 | clone 15F07VH1I2 CS1 immunoglobulin heavy chain variable region mRNA, partial cds | | *Ictalurus punctatus* |
| EU492767.1 | clone 15F11VH1I2 CS1 immunoglobulin heavy chain variable region mRNA, partial cds | | *Ictalurus punctatus* |
| EU492768.1 | clone 15F12VH1I2 CS1 immunoglobulin heavy chain variable region mRNA, partial cds | | *Ictalurus punctatus* |
| EU492769.1 | clone 15G02VH1I2 CS1 immunoglobulin heavy chain variable region mRNA, partial cds | | *Ictalurus punctatus* |
| EU492770.1 | clone 15G03VH1I2 CS1 immunoglobulin heavy chain variable region mRNA, partial cds | | *Ictalurus punctatus* |
| DQ230559.1 | clone 6F04AVH1 immunoglobulin heavy chain variable region mRNA, partial cds | | *Ictalurus punctatus* |
| M58673.1 | Ig heavy chain mRNA V-region clone NG64 | | *Ictalurus punctatus* |
| DQ230561.1 | clone 6G05AVH1 CS2 immunoglobulin heavy chain variable region mRNA, partial cds | | *Ictalurus punctatus* |
| EU492772.1 | clone 16F07VH2I2 immunoglobulin heavy chain variable region mRNA, partial cds | | *Ictalurus punctatus* |
| EU492773.1 | clone 16F08VH2I2 immunoglobulin heavy chain variable region mRNA, partial cds | | *Ictalurus punctatus* |
| EU492774.1 | clone 16F10VH2I2 CS7 immunoglobulin heavy chain variable region mRNA, partial cds | | *Ictalurus punctatus* |
| EU492776.1 | clone 16F12VH2I2 immunoglobulin heavy chain variable region mRNA, partial cds | | *Ictalurus punctatus* |
| DQ230565.1 | clone 1D11AVH2 CS1 immunoglobulin heavy chain variable region mRNA, partial cds | | *Ictalurus punctatus* |
| DQ230567.1 | clone 2C05AVH2 CS2 immunoglobulin heavy chain variable region mRNA, partial cds | | *Ictalurus punctatus* |
| DQ230568.1 | clone 2F06AVH2 CS1 immunoglobulin heavy chain variable region mRNA, partial cds | | *Ictalurus punctatus* |
| DQ230571.1 | clone 3B10AVH2 immunoglobulin heavy chain variable region mRNA, partial cds | | *Ictalurus punctatus* |
| DQ230572.1 | clone 3C07AVH2 immunoglobulin heavy chain variable region mRNA, partial cds | | *Ictalurus punctatus* |
| DQ230573.1 | clone 3C12AVH2 immunoglobulin heavy chain variable region mRNA, partial cds | | *Ictalurus punctatus* |
| DQ230574.1 | clone 3D09AVH2 immunoglobulin heavy chain variable region mRNA, partial cds | | *Ictalurus punctatus* |
| DQ230576.1 | clone 3D11AVH2 immunoglobulin heavy chain variable region mRNA, partial cds | | *Ictalurus punctatus* |
| DQ230577.1 | clone 6A03AVH2 immunoglobulin heavy chain variable region mRNA, partial cds | | *Ictalurus punctatus* |
| DQ230579.1 | clone 6D03AVH2 CS3 immunoglobulin heavy chain variable region mRNA, partial cds | | *Ictalurus punctatus* |
| DQ230580.1 | clone 6D06AVH2 CS3 immunoglobulin heavy chain variable region mRNA, partial cds | | *Ictalurus punctatus* |
| DQ230581.1 | clone 6E05AVH2 immunoglobulin heavy chain variable region mRNA, partial cds | | *Ictalurus punctatus* |
| EU492749.1 | clone 18E03VH2SK CS3 immunoglobulin heavy chain variable region mRNA, partial cds | | *Ictalurus punctatus* |
| EU492777.1 | clone 16G01VH2I2 immunoglobulin heavy chain variable region mRNA, partial cds | | *Ictalurus punctatus* |
| EU492704.1 | clone 16A01VH2GL immunoglobulin heavy chain variable region mRNA, partial cds | | *Ictalurus punctatus* |
| EU492648.1 | clone 16E04VH2SP immunoglobulin heavy chain variable region mRNA, partial cds | | *Ictalurus punctatus* |
| EU492649.1 | clone 16E05VH2SP immunoglobulin heavy chain variable region mRNA, partial cds | | *Ictalurus punctatus* |
| EU492650.1 | clone 16E07VH2SP immunoglobulin heavy chain variable region mRNA, partial cds | | *Ictalurus punctatus* |
| EU492651.1 | clone 16E08VH2SP immunoglobulin heavy chain variable region mRNA, partial cds | | *Ictalurus punctatus* |
| EU492652.1 | clone 16E09VH2SP immunoglobulin heavy chain variable region mRNA, partial cds | | *Ictalurus punctatus* |
| EU492653.1 | clone 16E10VH2SP immunoglobulin heavy chain variable region mRNA, partial cds | | *Ictalurus punctatus* |
| EU492655.1 | clone 16E12VH2SP immunoglobulin heavy chain variable region mRNA, partial cds | | *Ictalurus punctatus* |
| EU492656.1 | clone 16F01VH2SP immunoglobulin heavy chain variable region mRNA, partial cds | | *Ictalurus punctatus* |
| EU492657.1 | clone 16F02VH2SP immunoglobulin heavy chain variable region mRNA, partial cds | | *Ictalurus punctatus* |
| EU492658.1 | clone 16F03VH2SP immunoglobulin heavy chain variable region mRNA, partial cds | | *Ictalurus punctatus* |
| EU492585.1 | clone 20C02RevPBL CS2 immunoglobulin heavy chain variable region mRNA, partial cds | | *Ictalurus punctatus* |
| EU492589.1 | clone 19E03VH2PBL CS2 immunoglobulin heavy chain variable region mRNA, partial cds | | *Ictalurus punctatus* |
| EU492600.1 | clone 16D01VH2AK immunoglobulin heavy chain variable region mRNA, partial cds | | *Ictalurus punctatus* |
| EU492602.1 | clone 16D06VH2AK CS2 immunoglobulin heavy chain variable region mRNA, partial cds | | *Ictalurus punctatus* |
| EU492603.1 | clone 16D07VH2AK CS5 immunoglobulin heavy chain variable region mRNA, partial cds | | *Ictalurus punctatus* |
| EU492604.1 | clone 16D08VH2AK immunoglobulin heavy chain variable region mRNA, partial cds | | *Ictalurus punctatus* |
| EU492605.1 | clone 16D09VH2AK immunoglobulin heavy chain variable region mRNA, partial cds | | *Ictalurus punctatus* |
| EU492606.1 | clone 16D10VH2AK immunoglobulin heavy chain variable region mRNA, partial cds | | *Ictalurus punctatus* |
| EU492607.1 | clone 16D11RevAK immunoglobulin heavy chain variable region mRNA, partial cds | | *Ictalurus punctatus* |
| EU492608.1 | clone 16D12VH2AK immunoglobulin heavy chain variable region mRNA, partial cds | | *Ictalurus punctatus* |
| EU492633.1 | clone 19E04VH2AK CS2 immunoglobulin heavy chain variable region mRNA, partial cds | | *Ictalurus punctatus* |
| EU492638.1 | clone 15E03VH2SP immunoglobulin heavy chain variable region mRNA, partial cds | | *Ictalurus punctatus* |
| EU492639.1 | clone 15E04VH2SP immunoglobulin heavy chain variable region mRNA, partial cds | | *Ictalurus punctatus* |
| EU492647.1 | clone 16E03VH2SP immunoglobulin heavy chain variable region mRNA, partial cds | | *Ictalurus punctatus* |
| EU492659.1 | clone 16F04VH2SP immunoglobulin heavy chain variable region mRNA, partial cds | | *Ictalurus punctatus* |
| EU492660.1 | clone 16F05VH2SP immunoglobulin heavy chain variable region mRNA, partial cds | | *Ictalurus punctatus* |
| EU492691.1 | clone 20C04RevSP CS2 immunoglobulin heavy chain variable region mRNA, partial cds | | *Ictalurus punctatus* |
| EU492692.1 | clone 20E01RevSP CS1 immunoglobulin heavy chain variable region mRNA, partial cds | | *Ictalurus punctatus* |
| EU492705.1 | clone 16A02VH2GL immunoglobulin heavy chain variable region mRNA, partial cds | | *Ictalurus punctatus* |
| EU492706.1 | clone 16A03VH2GL immunoglobulin heavy chain variable region mRNA, partial cds | | *Ictalurus punctatus* |
| EU492708.1 | clone 16A05VH2GL CS9 immunoglobulin heavy chain variable region mRNA, partial cds | | *Ictalurus punctatus* |
| EU492709.1 | clone 16A06VH2GL immunoglobulin heavy chain variable region mRNA, partial cds | | *Ictalurus punctatus* |
| EU492710.1 | clone 16A07VH2GL immunoglobulin heavy chain variable region mRNA, partial cds | | *Ictalurus punctatus* |
| EU492711.1 | clone 16A09VH2GL immunoglobulin heavy chain variable region mRNA, partial cds | | *Ictalurus punctatus* |
| EU492712.1 | clone 16A11VH2GL immunoglobulin heavy chain variable region mRNA, partial cds | | *Ictalurus punctatus* |
| EU492713.1 | clone 16A12VH2GL immunoglobulin heavy chain variable region mRNA, partial cds | | *Ictalurus punctatus* |
| EU492735.1 | clone 20F02VH2GL immunoglobulin heavy chain variable region mRNA, partial cds | | *Ictalurus punctatus* |
| DQ230582.1 | clone 6F06AVH2 immunoglobulin heavy chain variable region mRNA, partial cds | | *Ictalurus punctatus* |
| DQ230583.1 | clone 6G06AVH2 CS2 immunoglobulin heavy chain variable region mRNA, partial cds | | *Ictalurus punctatus* |
| DQ230584.1 | clone 6H06AVH2 immunoglobulin heavy chain variable region mRNA, partial cds | | *Ictalurus punctatus* |
| AY238359.1 | immunoglobulin heavy chain variable region mRNA, partial cds | | *Ictalurus punctatus* |
| EU492559.1 | clone 16B04VH2PBL immunoglobulin heavy chain variable region mRNA, partial cds | | *Ictalurus punctatus* |
| EU492560.1 | clone 16B06VH2PBL immunoglobulin heavy chain variable region mRNA, partial cds | | *Ictalurus punctatus* |
| EU492561.1 | clone 16B08VH2PBL immunoglobulin heavy chain variable region mRNA, partial cds | | *Ictalurus punctatus* |
| EU492562.1 | clone 16B10VH2PBL immunoglobulin heavy chain variable region mRNA, partial cds | | *Ictalurus punctatus* |
| EU492563.1 | clone 16B12VH2PBL immunoglobulin heavy chain variable region mRNA, partial cds | | *Ictalurus punctatus* |
| EU492564.1 | clone 16C01VH2PBL immunoglobulin heavy chain variable region mRNA, partial cds | | *Ictalurus punctatus* |
| EU492565.1 | clone 16C02VH2PBL immunoglobulin heavy chain variable region mRNA, partial cds | | *Ictalurus punctatus* |
| EU492566.1 | clone 16C03VH2PBL immunoglobulin heavy chain variable region mRNA, partial cds | | *Ictalurus punctatus* |
| EU492821.1 | clone 16H03VH2I3 CS1 immunoglobulin heavy chain variable region mRNA, partial cds | | *Ictalurus punctatus* |
| EU492568.1 | clone 16C05VH2PBL immunoglobulin heavy chain variable region mRNA, partial cds | | *Ictalurus punctatus* |
| EU492822.1 | clone 16H09VH2I3 immunoglobulin heavy chain variable region mRNA, partial cds | | *Ictalurus punctatus* |
| EU492812.1 | clone 20F03RevI2 CS8 immunoglobulin heavy chain variable region mRNA, partial cds | | *Ictalurus punctatus* |
| EU492736.1 | clone 16A08RevI2 CS8 immunoglobulin heavy chain variable region mRNA, partial cds | | *Ictalurus punctatus* |
| EU492760.1 | clone 21C06VH2SK immunoglobulin heavy chain variable region mRNA, partial cds | | *Ictalurus punctatus* |
| EU492778.1 | clone 16G02VH2I2 CS9 immunoglobulin heavy chain variable region mRNA, partial cds | | *Ictalurus punctatus* |
| EU492853.1 | clone 16H05VH2I3 CS5 immunoglobulin heavy chain variable region mRNA, partial cds | | *Ictalurus punctatus* |
| DQ230570.1 | clone 3B05AVH2 CS4 immunoglobulin heavy chain variable region mRNA, partial cds | | *Ictalurus punctatus* |
| DQ230575.1 | clone 3D10AVH2 CS1 immunoglobulin heavy chain variable region mRNA, partial cds | | *Ictalurus punctatus* |
| DQ230578.1 | clone 6B05AVH2 CS1 immunoglobulin heavy chain variable region mRNA, partial cds | | *Ictalurus punctatus* |
| EU492599.1 | clone 16C12VH2AK CS2 immunoglobulin heavy chain variable region mRNA, partial cds | | *Ictalurus punctatus* |
| EU492654.1 | clone 16E11VH2SP immunoglobulin heavy chain variable region mRNA, partial cds | | *Ictalurus punctatus* |
| EU492694.1 | clone 19E11VH2SP CS2 immunoglobulin heavy chain variable region mRNA, partial cds | | *Ictalurus punctatus* |
| EU492758.1 | clone 20C09VH2SK immunoglobulin heavy chain variable region mRNA, partial cds | | *Ictalurus punctatus* |
| EU492810.1 | clone 16G06RevI2 CS2 immunoglobulin heavy chain variable region mRNA, partial cds | | *Ictalurus punctatus* |
| EU492823.1 | clone 16H10VH2I3 CS1 immunoglobulin heavy chain variable region mRNA, partial cds | | *Ictalurus punctatus* |
| EU492846.1 | clone 21H05VH2I3 immunoglobulin heavy chain variable region mRNA, partial cds | | *Ictalurus punctatus* |
| EU492847.1 | clone 21H06RevI3 immunoglobulin heavy chain variable region mRNA, partial cds | | *Ictalurus punctatus* |
| EU492849.1 | clone 21H09VH2I3 immunoglobulin heavy chain variable region mRNA, partial cds | | *Ictalurus punctatus* |
| EU492852.1 | clone 16H07RevI3 CS1 immunoglobulin heavy chain variable region mRNA, partial cds | | *Ictalurus punctatus* |
| M58670.1 | Ig heavy chain mRNA V-region clone NG22 | | *Ictalurus punctatus* |
| EU492779.1 | clone 16G04VH2I2 immunoglobulin heavy chain variable region mRNA, partial cds | | *Ictalurus punctatus* |
| M58675.1 | Ig heavy chain mRNA V-region, clone NG77 | | *Ictalurus punctatus* |
| EU492841.1 | clone 20G04VH2I3 CS6 immunoglobulin heavy chain variable region mRNA, partial cds | | *Ictalurus punctatus* |
| EU492780.1 | clone 16G08VH2I2 CS7 immunoglobulin heavy chain variable region mRNA, partial cds | | *Ictalurus punctatus* |
| EU492707.1 | clone 16A04VH2GL immunoglobulin heavy chain variable region mRNA, partial cds | | *Ictalurus punctatus* |
| EU492569.1 | clone 16C06VH2PBL immunoglobulin heavy chain variable region mRNA, partial cds | | *Ictalurus punctatus* |
| EU492601.1 | clone 16D04VH2AK CS6 immunoglobulin heavy chain variable region mRNA, partial cds | | *Ictalurus punctatus* |
| AF273412.1 | clone 01-09 immunoglobulin heavy chain variable region (IgH) mRNA, partial cds | | *Salmo salar* |
| AF273415.1 | clone 05-12 immunoglobulin heavy chain variable region (IgH) mRNA, partial cds | | *Salmo salar* |
| AF273410.1 | clone 04-09 immunoglobulin heavy chain variable region (IgH) mRNA, partial cds | | *Salmo salar* |
| AF273411.1 | clone 01-06 immunoglobulin heavy chain variable region (IgH) mRNA, partial cds | | *Salmo salar* |
| AF273413.1 | clone 01-10 immunoglobulin heavy chain variable region (IgH) mRNA, partial cds | | *Salmo salar* |
| AF273414.1 | clone 05-11 immunoglobulin heavy chain variable region (IgH) mRNA, partial cds | | *Salmo salar* |
| AF273416.1 | clone 08-06 immunoglobulin heavy chain variable region (IgH) mRNA, partial cds | | *Salmo salar* |
| AF273417.1 | clone 08-07 immunoglobulin heavy chain variable region (IgH) mRNA, partial cds | | *Salmo salar* |
| AF273418.1 | clone 10-11 immunoglobulin heavy chain variable region (IgH) mRNA, partial cds | | *Salmo salar* |
| AF269076.1 | clone d27 immunoglobulin heavy chain variable region (IgH) mRNA, partial cds | | *Salmo salar* |
| AF269078.1 | clone 09-10 immunoglobulin heavy chain variable region (IgH) mRNA, partial cds | | *Salmo salar* |
| AF273398.1 | clone 08-01 immunoglobulin heavy chain variable region (IgH) mRNA, partial cds | | *Salmo salar* |
| AF273399.1 | clone 08-10 immunoglobulin heavy chain variable region (IgH) mRNA, partial cds | | *Salmo salar* |
| AF273396.1 | clone 04-04 immunoglobulin heavy chain variable region (IgH) mRNA, partial cds | | *Salmo salar* |
| AF273397.1 | clone 07-02 immunoglobulin heavy chain variable region (IgH) mRNA, partial cds | | *Salmo salar* |
| AF269079.1 | clone 02-04 immunoglobulin heavy chain variable region (IgH) mRNA, partial cds | | *Salmo salar* |
| AF269080.1 | clone 02-10 immunoglobulin heavy chain variable region (IgH) mRNA, partial cds | | *Salmo salar* |
| AF269081.1 | clone 06-07 immunoglobulin heavy chain variable region (IgH) mRNA, partial cds | | *Salmo salar* |
| AF269082.1 | clone 09-04 immunoglobulin heavy chain variable region (IgH) mRNA, partial cds | | *Salmo salar* |
| AF269083.1 | clone 09-06 immunoglobulin heavy chain variable region (IgH) mRNA, partial cds | | *Salmo salar* |
| AF273429.1 | clone 07-04 immunoglobulin heavy chain variable region (IgH) mRNA, partial cds | | *Salmo salar* |
| AF269084.1 | clone 09-14 immunoglobulin heavy chain variable region (IgH) mRNA, partial cds | | *Salmo salar* |
| AF273425.1 | clone 03-08 immunoglobulin heavy chain variable region (IgH) mRNA, partial cds | | *Salmo salar* |
| AF273426.1 | clone 10-08 immunoglobulin heavy chain variable region (IgH) mRNA, partial cds | | *Salmo salar* |
| AF273419.1 | clone 01-03 immunoglobulin heavy chain variable region (IgH) mRNA, partial cds | | *Salmo salar* |
| AF273421.1 | clone 08-05 immunoglobulin heavy chain variable region (IgH) mRNA, partial cds | | *Salmo salar* |
| AF273422.1 | clone 09-15 immunoglobulin heavy chain variable region (IgH) mRNA, partial cds | | *Salmo salar* |
| AF273423.1 | clone 09-12 immunoglobulin heavy chain variable region (IgH) mRNA, partial cds | | *Salmo salar* |
| AF273424.1 | clone 10-02 immunoglobulin heavy chain variable region (IgH) mRNA, partial cds | | *Salmo salar* |
| AF273420.1 | clone 07-01 immunoglobulin heavy chain variable region (IgH) mRNA, partial cds | | *Salmo salar* |
| AF273427.1 | clone 02-05 immunoglobulin heavy chain variable region (IgH) mRNA, partial cds | | *Salmo salar* |
| AF273428.1 | clone 03-06 immunoglobulin heavy chain variable region (IgH) mRNA, partial cds | | *Salmo salar* |
| AF273430.1 | clone 08-03 immunoglobulin heavy chain variable region (IgH) mRNA, partial cds | | *Salmo salar* |
| AF273431.1 | clone 09-03 immunoglobulin heavy chain variable region (IgH) mRNA, partial cds | | *Salmo salar* |
| AF273432.1 | clone 10-10 immunoglobulin heavy chain variable region (IgH) mRNA, partial cds | | *Salmo salar* |
| AF269085.1 | clone 10-06 immunoglobulin heavy chain variable region (IgH) mRNA, partial cds | | *Salmo salar* |
| AY646275.1 | isolate 4-8.2.1 immunoglobulin zeta heavy chain mRNA, partial cds | | *Danio rerio* |
| AY646273.1 | isolate 4-3.2.1 immunoglobulin zeta heavy chain mRNA, partial cds | | *Danio rerio* |
| AY646274.1 | isolate 4-6.0.2 immunoglobulin zeta heavy chain mRNA, partial cds | | *Danio rerio* |
| AY646252.1 | isolate 4-8.3.5 immunoglobulin mu heavy chain mRNA, partial cds | | *Danio rerio* |
| AY646251.1 | isolate 4-6.5.5 immunoglobulin mu heavy chain mRNA, partial cds | | *Danio rerio* |
| AY646250.1 | isolate 4-6.4.2 immunoglobulin mu heavy chain mRNA, partial cds | | *Danio rerio* |
| AF273884.1 | clone VH124 immunoglobulin heavy chain variable region mRNA, partial cds | | *Danio rerio* |
| AF273876.1 | clone VH101 immunoglobulin heavy chain variable region mRNA, partial cds | | *Danio rerio* |
| AF273877.1 | clone VH103 immunoglobulin heavy chain variable region mRNA, partial cds | | *Danio rerio* |
| AF273878.1 | clone VH119 immunoglobulin heavy chain variable region mRNA, partial cds | | *Danio rerio* |
| AF273882.1 | clone VH23 immunoglobulin heavy chain variable region mRNA, partial cds | | *Danio rerio* |
| AF273885.1 | clone VH88 immunoglobulin heavy chain variable region mRNA, partial cds | | *Danio rerio* |
| AF273880.1 | clone VH114 immunoglobulin heavy chain variable region mRNA, partial cds | | *Danio rerio* |
| AF273886.1 | clone VH350-6 immunoglobulin heavy chain variable region mRNA, partial cds | | *Danio rerio* |
| AF273889.1 | clone VH350-3 immunoglobulin heavy chain variable region mRNA, partial cds | | *Danio rerio* |
| AY646245.1 | isolate 1-2.1.1 immunoglobulin mu heavy chain mRNA, partial cds | | *Danio rerio* |
| DQ106021.1 | isolate A variant immunoglobulin heavy chain variable region gene, partial cds | | *Danio rerio* |
| AF273897.1 | clone VHE1 immunoglobulin heavy chain variable region gene, partial cds | | *Danio rerio* |
| DQ106019.1 | isolate A immunoglobulin heavy chain variable region gene, partial cds | | *Danio rerio* |
| AY646263.1 | isolate 1-2.2.1 immunoglobulin zeta heavy chain mRNA, partial cds | | *Danio rerio* |
| AY646264.1 | isolate 1-1.2.1 immunoglobulin zeta heavy chain mRNA, partial cds | | *Danio rerio* |
| AY646267.1 | isolate 1-2.1.1 immunoglobulin zeta heavy chain mRNA, partial cds | | *Danio rerio* |
| AY608342.1 | isolate MaryM7 immunoglobulin mu heavy chain variable region mRNA, partial cds | | *Ginglymostoma cirratum* |
| AY608355.1 | isolate MaryM33 immunoglobulin mu heavy chain variable region mRNA, partial cds | | *Ginglymostoma cirratum* |
| AY608358.1 | isolate JosefM2 immunoglobulin mu heavy chain variable region mRNA, partial cds | | *Ginglymostoma cirratum* |
| AY608362.1 | isolate JosefM7 immunoglobulin mu heavy chain variable region mRNA, partial cds | | *Ginglymostoma cirratum* |
| AY608373.1 | isolate JosefM21 immunoglobulin mu heavy chain variable region mRNA, partial cds | | *Ginglymostoma cirratum* |
| AY608376.1 | isolate JosefM27 immunoglobulin mu heavy chain variable region mRNA, partial cds | | *Ginglymostoma cirratum* |
| AY608386.1 | isolate M17 immunoglobulin mu heavy chain variable region mRNA, partial cds | | *Ginglymostoma cirratum* |
| AY608392.1 | isolate M29 immunoglobulin mu heavy chain variable region mRNA, partial cds | | *Ginglymostoma cirratum* |
| AY608397.1 | isolate M34 immunoglobulin mu heavy chain variable region mRNA, partial cds | | *Ginglymostoma cirratum* |
| AY609272.1 | clone 72S immunoglobulin mu heavy chain variable region mRNA, partial cds | | *Ginglymostoma cirratum* |
| AY609265.1 | clone 47S immunoglobulin mu heavy chain variable region mRNA, partial cds | | *Ginglymostoma cirratum* |
| AY609266.1 | clone 49S immunoglobulin mu heavy chain variable region mRNA, partial cds | | *Ginglymostoma cirratum* |
| GQ359839.1 | clone G5G2-13 immunoglobulin heavy chain variable region mRNA, partial cds | | *Ginglymostoma cirratum* |
| GQ359840.1 | clone G5G2-16 immunoglobulin heavy chain variable region mRNA, partial cds | | *Ginglymostoma cirratum* |
| GQ359841.1 | clone G5G2-17 immunoglobulin heavy chain variable region mRNA, partial cds | | *Ginglymostoma cirratum* |
| GQ359843.1 | clone G5G2-33 immunoglobulin heavy chain variable region mRNA, partial cds | | *Ginglymostoma cirratum* |
| GQ359844.1 | clone G5G2-9 immunoglobulin heavy chain variable region mRNA, partial cds | | *Ginglymostoma cirratum* |
| GQ359845.1 | clone G5G2-13-2 immunoglobulin heavy chain variable region mRNA, partial cds | | *Ginglymostoma cirratum* |
| GQ359846.1 | clone G5G2-B immunoglobulin heavy chain variable region mRNA, partial cds | | *Ginglymostoma cirratum* |
| GQ359832.1 | clone G2G5-E11 immunoglobulin heavy chain variable region mRNA, partial cds | | *Ginglymostoma cirratum* |
| GQ359857.1 | clone G2G5-C2 immunoglobulin heavy chain variable region mRNA, partial cds | | *Ginglymostoma cirratum* |
| GQ359856.1 | clone G2G5-B9 immunoglobulin heavy chain variable region mRNA, partial cds | | *Ginglymostoma cirratum* |
| GQ359858.1 | clone G2G5-C6 immunoglobulin heavy chain variable region mRNA, partial cds | | *Ginglymostoma cirratum* |
| AY608337.1 | isolate MaryM2 immunoglobulin mu heavy chain variable region mRNA, partial cds | | *Ginglymostoma cirratum* |
| AY608339.1 | isolate MaryM4 immunoglobulin mu heavy chain variable region mRNA, partial cds | | *Ginglymostoma cirratum* |
| AY608340.1 | isolate MaryM5 immunoglobulin mu heavy chain variable region mRNA, partial cds | | *Ginglymostoma cirratum* |
| AY608341.1 | isolate MaryM6 immunoglobulin mu heavy chain variable region mRNA, partial cds | | *Ginglymostoma cirratum* |
| AY608346.1 | isolate MaryM12 immunoglobulin mu heavy chain variable region mRNA, partial cds | | *Ginglymostoma cirratum* |
| AY608347.1 | isolate MaryM13 immunoglobulin mu heavy chain variable region mRNA, partial cds | | *Ginglymostoma cirratum* |
| AY608349.1 | isolate MaryM15 immunoglobulin mu heavy chain variable region mRNA, partial cds | | *Ginglymostoma cirratum* |
| AY608351.1 | isolate MaryM17 immunoglobulin mu heavy chain variable region mRNA, partial cds | | *Ginglymostoma cirratum* |
| AY608353.1 | isolate MaryM31 immunoglobulin mu heavy chain variable region mRNA, partial cds | | *Ginglymostoma cirratum* |
| AY608354.1 | isolate MaryM32 immunoglobulin mu heavy chain variable region mRNA, partial cds | | *Ginglymostoma cirratum* |
| AY608356.1 | isolate MaryM34 immunoglobulin mu heavy chain variable region mRNA, partial cds | | *Ginglymostoma cirratum* |
| AY608357.1 | isolate JosefM1 immunoglobulin mu heavy chain variable region mRNA, partial cds | | *Ginglymostoma cirratum* |
| AY608361.1 | isolate JosefM6 immunoglobulin mu heavy chain variable region mRNA, partial cds | | *Ginglymostoma cirratum* |
| AY608363.1 | isolate JosefM11 immunoglobulin mu heavy chain variable region mRNA, partial cds | | *Ginglymostoma cirratum* |
| AY608364.1 | isolate JosefM12 immunoglobulin mu heavy chain variable region mRNA, partial cds | | *Ginglymostoma cirratum* |
| AY608365.1 | isolate JosefM13 immunoglobulin mu heavy chain variable region mRNA, partial cds | | *Ginglymostoma cirratum* |
| AY608366.1 | isolate JosefM14 immunoglobulin mu heavy chain variable region mRNA, partial cds | | *Ginglymostoma cirratum* |
| AY608367.1 | isolate JosefM15 immunoglobulin mu heavy chain variable region mRNA, partial cds | | *Ginglymostoma cirratum* |
| AY608368.1 | isolate JosefM16 immunoglobulin mu heavy chain variable region mRNA, partial cds | | *Ginglymostoma cirratum* |
| AY608369.1 | isolate JosefM17 immunoglobulin mu heavy chain variable region mRNA, partial cds | | *Ginglymostoma cirratum* |
| AY608370.1 | isolate JosefM18 immunoglobulin mu heavy chain variable region mRNA, partial cds | | *Ginglymostoma cirratum* |
| AY608371.1 | isolate JosefM19 immunoglobulin mu heavy chain variable region mRNA, partial cds | | *Ginglymostoma cirratum* |
| AY608372.1 | isolate JosefM20 immunoglobulin mu heavy chain variable region mRNA, partial cds | | *Ginglymostoma cirratum* |
| AY608374.1 | isolate JosefM22 immunoglobulin mu heavy chain variable region mRNA, partial cds | | *Ginglymostoma cirratum* |
| AY608375.1 | isolate JosefM26 immunoglobulin mu heavy chain variable region mRNA, partial cds | | *Ginglymostoma cirratum* |
| AY608377.1 | isolate M3 immunoglobulin mu heavy chain variable region mRNA, partial cds | | *Ginglymostoma cirratum* |
| AY608378.1 | isolate M4 immunoglobulin mu heavy chain variable region mRNA, partial cds | | *Ginglymostoma cirratum* |
| AY608379.1 | isolate M5 immunoglobulin mu heavy chain variable region mRNA, partial cds | | *Ginglymostoma cirratum* |
| AY608381.1 | isolate M8 immunoglobulin mu heavy chain variable region mRNA, partial cds | | *Ginglymostoma cirratum* |
| AY608382.1 | isolate M9 immunoglobulin mu heavy chain variable region mRNA, partial cds | | *Ginglymostoma cirratum* |
| AY608383.1 | isolate M13 immunoglobulin mu heavy chain variable region mRNA, partial cds | | *Ginglymostoma cirratum* |
| AY608384.1 | isolate M14 immunoglobulin mu heavy chain variable region mRNA, partial cds | | *Ginglymostoma cirratum* |
| AY608385.1 | isolate M15 immunoglobulin mu heavy chain variable region mRNA, partial cds | | *Ginglymostoma cirratum* |
| AY608387.1 | isolate M19 immunoglobulin mu heavy chain variable region mRNA, partial cds | | *Ginglymostoma cirratum* |
| AY608389.1 | isolate M21 immunoglobulin mu heavy chain variable region mRNA, partial cds | | *Ginglymostoma cirratum* |
| AY608390.1 | isolate M24 immunoglobulin mu heavy chain variable region mRNA, partial cds | | *Ginglymostoma cirratum* |
| AY608391.1 | isolate M25 immunoglobulin mu heavy chain variable region mRNA, partial cds | | *Ginglymostoma cirratum* |
| AY608393.1 | isolate M30 immunoglobulin mu heavy chain variable region mRNA, partial cds | | *Ginglymostoma cirratum* |
| AY608394.1 | isolate M31 immunoglobulin mu heavy chain variable region mRNA, partial cds | | *Ginglymostoma cirratum* |
| AY608395.1 | isolate M32 immunoglobulin mu heavy chain variable region mRNA, partial cds | | *Ginglymostoma cirratum* |
| AY608396.1 | isolate M33 immunoglobulin mu heavy chain variable region mRNA, partial cds | | *Ginglymostoma cirratum* |
| AY608398.1 | isolate M35 immunoglobulin mu heavy chain variable region mRNA, partial cds | | *Ginglymostoma cirratum* |
| AY608400.1 | isolate M39 immunoglobulin mu heavy chain variable region mRNA, partial cds | | *Ginglymostoma cirratum* |
| AY608401.1 | isolate M41 immunoglobulin mu heavy chain variable region mRNA, partial cds | | *Ginglymostoma cirratum* |
| AY608403.1 | isolate M43 immunoglobulin mu heavy chain variable region mRNA, partial cds | | *Ginglymostoma cirratum* |
| AY608404.1 | isolate M47 immunoglobulin mu heavy chain variable region mRNA, partial cds | | *Ginglymostoma cirratum* |
| AY609249.1 | clone 2S immunoglobulin mu heavy chain variable region mRNA, partial cds | | *Ginglymostoma cirratum* |
| AY609254.1 | clone 21S immunoglobulin mu heavy chain variable region mRNA, partial cds | | *Ginglymostoma cirratum* |
| GQ359842.1 | clone G5G2-31 immunoglobulin heavy chain variable region mRNA, partial cds | | *Ginglymostoma cirratum* |
| AY609258.1 | clone 27S immunoglobulin mu heavy chain variable region mRNA, partial cds | | *Ginglymostoma cirratum* |
| GQ282627.1 | clone G2G5-34 immunoglobulin heavy chain variable region mRNA, partial cds | | *Ginglymostoma cirratum* |
| AY609264.1 | clone 46S immunoglobulin mu heavy chain variable region mRNA, partial cds | | *Ginglymostoma cirratum* |
| AY609259.1 | clone 29S immunoglobulin mu heavy chain variable region mRNA, partial cds | | *Ginglymostoma cirratum* |
| GQ359827.1 | clone G4G5-3 immunoglobulin heavy chain variable region mRNA, partial cds | | *Ginglymostoma cirratum* |
| GQ359826.1 | clone G4G5-17 immunoglobulin heavy chain variable region mRNA, partial cds | | *Ginglymostoma cirratum* |
| GQ359833.1 | clone G4G5-E30 immunoglobulin heavy chain variable region mRNA, partial cds | | *Ginglymostoma cirratum* |
| GQ359828.1 | clone G4G5-4 immunoglobulin heavy chain variable region mRNA, partial cds | | *Ginglymostoma cirratum* |
| GQ359830.1 | clone G4G5-39 immunoglobulin heavy chain variable region mRNA, partial cds | | *Ginglymostoma cirratum* |
| GQ359829.1 | clone G4G5-33 immunoglobulin heavy chain variable region mRNA, partial cds | | *Ginglymostoma cirratum* |
| GQ359831.1 | clone G4G5-66 immunoglobulin heavy chain variable region mRNA, partial cds | | *Ginglymostoma cirratum* |
| GQ359835.1 | clone G4G2-33 immunoglobulin heavy chain variable region mRNA, partial cds | | *Ginglymostoma cirratum* |
| GQ359836.1 | clone G4G2-41 immunoglobulin heavy chain variable region mRNA, partial cds | | *Ginglymostoma cirratum* |
| GQ359834.1 | clone G4G5-E35 immunoglobulin heavy chain variable region mRNA, partial cds | | *Ginglymostoma cirratum* |
| GQ359837.1 | clone G4G2-54 immunoglobulin heavy chain variable region mRNA, partial cds | | *Ginglymostoma cirratum* |
| AY609252.1 | clone 15S | | *Ginglymostoma cirratum* |
| GQ359848.1 | clone G4G5-46 immunoglobulin heavy chain variable region mRNA, partial cds | | *Ginglymostoma cirratum* |
| GQ359849.1 | clone G4G5-76 immunoglobulin heavy chain variable region mRNA, partial cds | | *Ginglymostoma cirratum* |
| GQ359850.1 | clone G4G5-81 immunoglobulin heavy chain variable region mRNA, partial cds | | *Ginglymostoma cirratum* |
| GQ359855.1 | clone G4G5-A21 immunoglobulin heavy chain variable region mRNA, partial cds | | *Ginglymostoma cirratum* |
| GQ359851.1 | clone G4G5-88 immunoglobulin heavy chain variable region mRNA, partial cds | | *Ginglymostoma cirratum* |
| JQ272797.1 | clone 4-7 IgM G4 VDJ switch to G5 C-region mRNA sequence | | *Ginglymostoma cirratum* |
| JQ272798.1 | clone 4-21 IgM G4 VDJ switch to G5 C-region mRNA sequence | | *Ginglymostoma cirratum* |
| JQ272799.1 | clone 4-36 IgM G4 VDJ switch to G5 C-region mRNA sequence | | *Ginglymostoma cirratum* |
| JQ272805.1 | clone I7 IgM G4 VDJ switch to G5 C-region mRNA sequence | | *Ginglymostoma cirratum* |
| JQ272806.1 | clone I16 IgM G4 VDJ switch to G5 C-region mRNA sequence | | *Ginglymostoma cirratum* |
| JQ272808.1 | clone I29 IgM G4 VDJ switch to G5 C-region mRNA sequence | | *Ginglymostoma cirratum* |
| JQ272809.1 | clone I36 IgM G4 VDJ switch to G5 C-region mRNA sequence | | *Ginglymostoma cirratum* |
| JQ272810.1 | clone I53 IgM G4 VDJ switch to G5 C-region mRNA sequence | | *Ginglymostoma cirratum* |
| JQ272812.1 | clone I69 IgM G4 VDJ switch to G5 C-region mRNA sequence | | *Ginglymostoma cirratum* |
| JQ272815.1 | clone I-167 IgM G4 VDJ switch to G5 C-region mRNA sequence | | *Ginglymostoma cirratum* |
| JQ272821.1 | clone 61 IgM G5 VDJ switch to G4 C-region mRNA sequence | | *Ginglymostoma cirratum* |
| GQ359852.1 | clone G2G5-F27 immunoglobulin heavy chain variable region mRNA, partial cds | | *Ginglymostoma cirratum* |
| GQ359853.1 | clone G4G5-C33 immunoglobulin heavy chain variable region mRNA, partial cds | | *Ginglymostoma cirratum* |
| JF507607.1 | clone T0923W2J05 IgWV TCR delta trans-rearrangement (TCRD) mRNA, partial cds | | *Ginglymostoma cirratum* |
| JF507611.1 | clone T1023W2J12 IgWV TCR delta trans-rearrangement (TCRD) mRNA, partial cds | | *Ginglymostoma cirratum* |
| JF507612.1 | clone T1123W2J12 IgWV TCR delta trans-rearrangement (TCRD) mRNA, partial cds | | *Ginglymostoma cirratum* |
| JF507613.1 | clone T0419W2J12 IgWV TCR delta trans-rearrangement (TCRD) mRNA, partial cds | | *Ginglymostoma cirratum* |
| JF507614.1 | clone T0423W2J12 IgWV TCR delta trans-rearrangement (TCRD) mRNA, partial cds | | *Ginglymostoma cirratum* |
| JF507615.1 | clone T1323W2J12 IgWV TCR delta trans-rearrangement (TCRD) mRNA, partial cds | | *Ginglymostoma cirratum* |
| JF507616.1 | clone T0523W2J24 IgWV TCR delta trans-rearrangement (TCRD) mRNA, partial cds | | *Ginglymostoma cirratum* |
| JF507617.1 | clone T0123W2J24 IgWV TCR delta trans-rearrangement (TCRD) mRNA, partial cds | | *Ginglymostoma cirratum* |
| JF507620.1 | clone S1523W2J06 IgWV TCR delta trans-rearrangement (TCRD) mRNA, partial cds | | *Ginglymostoma cirratum* |
| JF507625.1 | clone S0916W2J08 IgWV TCR delta trans-rearrangement (TCRD) mRNA, partial cds | | *Ginglymostoma cirratum* |
| JF507627.1 | clone S2023W2J09 IgWV TCR delta trans-rearrangement (TCRD) mRNA, partial cds | | *Ginglymostoma cirratum* |
| JF507629.1 | clone S1823W2J12 IgWV TCR delta trans-rearrangement (TCRD) mRNA, partial cds | | *Ginglymostoma cirratum* |
| JF507637.1 | clone V1419W2J08 IgWV TCR delta trans-rearrangement (TCRD) mRNA, partial cds | | *Ginglymostoma cirratum* |
| JF507640.1 | clone V1319W2J08 IgWV TCR delta trans-rearrangement (TCRD) mRNA, partial cds | | *Ginglymostoma cirratum* |
| JF507646.1 | clone V1924W2J12 IgWV TCR delta trans-rearrangement (TCRD) mRNA, partial cds | | *Ginglymostoma cirratum* |
| JF507647.1 | clone V1724W2J12 IgWV TCR delta trans-rearrangement (TCRD) mRNA, partial cds | | *Ginglymostoma cirratum* |
| JF507648.1 | clone V2424W2J12 IgWV TCR delta trans-rearrangement (TCRD) mRNA, partial cds | | *Ginglymostoma cirratum* |
| JF507659.1 | clone V1219W2J24 IgWV TCR delta trans-rearrangement (TCRD) mRNA, partial cds | | *Ginglymostoma cirratum* |
| JF507660.1 | clone V1424W2J25 IgWV TCR delta trans-rearrangement (TCRD) mRNA, partial cds | | *Ginglymostoma cirratum* |
| KC920802.1 | clone V5 secreted IgW heavy chain mRNA, partial cds | | *Ginglymostoma cirratum* |
| KC920803.1 | clone c1 secreted IgW heavy chain mRNA, partial cds | | *Ginglymostoma cirratum* |
| AY524282.1 | clone 7 1-2 immunoglobulin IgW short mRNA complete cds | | *Ginglymostoma cirratum* |
| AY524295.1 | clone L immunoglobulin IgW-like mRNA complete sequence | | *Ginglymostoma cirratum* |
| LC000730.1 | IGHV2S19 gene immunoglobulin heavy chain partial sequence | | *Takifugu rubripes* |
| LC000729.1 | IGHV2S18 gene immunoglobulin heavy chain partial sequence | | *Takifugu rubripes* |
| AB125608.1 | IgVH mRNA for immunoglobulin heavy chain variable region partial cds clone: F-m161 | | *Takifugu rubripes* |
| LC000719.1 | IGHV2S7 gene immunoglobulin heavy chain partial sequence | | *Takifugu rubripes* |
| AB125607.1 | IgVH mRNA for immunoglobulin heavy chain variable region partial cds clone: F-m146 | | *Takifugu rubripes* |
| AB125606.1 | IgVH mRNA for immunoglobulin heavy chain variable region partial cds clone: F-m118 | | *Takifugu rubripes* |
| AB217624.1 | IgM mRNA for immunoglobulin mu heavy chain partial cds clone: IgM_36 | | *Takifugu rubripes* |
| XM_011621003.1 | Ig mu chain C region membrane-bound form (LOC445921) mRNA | | *Takifugu rubripes* |
| LC000729.1 | IGHV2S18 gene immunoglobulin heavy chain partial sequence | | *Takifugu rubripes* |
| LC000728.1 | IGHV2S17 gene immunoglobulin heavy chain partial sequence | | *Takifugu rubripes* |
| LC000724.1 | IGHV2S12 gene immunoglobulin heavy chain partial sequence | | *Takifugu rubripes* |
| LC000720.1 | IGHV2S8 gene immunoglobulin heavy chain partial sequence | | *Takifugu rubripes* |
| LC000719.1 | IGHV2S7 gene immunoglobulin heavy chain partial sequence | | *Takifugu rubripes* |
| LC000718.1 | IGHV2S6 gene immunoglobulin heavy chain partial sequence | | *Takifugu rubripes* |
| LC000717.1 | IGHV2S1 gene immunoglobulin heavy chain partial sequence | | *Takifugu rubripes* |
| LC000721.1 | IGHV2S9 gene immunoglobulin heavy chain partial sequence | | *Takifugu rubripes* |
| LC000722.1 | IGHV2S10 gene immunoglobulin heavy chain partial sequence | | *Takifugu rubripes* |
| LC000723.1 | IGHV2S11 gene immunoglobulin heavy chain partial sequence | | *Takifugu rubripes* |
| LC000726.1 | IGHV2S15 gene immunoglobulin heavy chain partial sequence | | *Takifugu rubripes* |
| LC000727.1 | IGHV2S16 gene immunoglobulin heavy chain partial sequence | | *Takifugu rubripes* |
| LC000730.1 | IGHV2S19 gene immunoglobulin heavy chain partial sequence | | *Takifugu rubripes* |
| LC000731.1 | IGHV2S20 gene immunoglobulin heavy chain partial sequence | | *Takifugu rubripes* |
| AB217616.1 | IgH mRNA for Immunoglobulin heavy chain partial cds clone: IgH_4 | | *Takifugu rubripes* |
| AB159481.1 | IgD mRNA for immunoglobulin D complete cds | | *Takifugu rubripes* |
| AB217618.1 | IgH mRNA for immunoglobulin heavy chain partial cds clone: IgH_6 | | *Takifugu rubripes* |
| AB125605.1 | IgVH mRNA for immunoglobulin heavy chain variable region partial cds clone: F-m116 | | *Takifugu rubripes* |
| AB217620.1 | IgH mRNA for immunoglobulin heavy chain partial cds clone: IgH_20 | | *Takifugu rubripes* |
| AB125604.1 | IgVH mRNA for immunoglobulin heavy chain variable region partial cds clone: F-m106 | | *Takifugu rubripes* |
| LC000713.2 | IGHV1S17 gene immunoglobulin heavy chain partial sequence | | *Takifugu rubripes* |
| LC000716.1 | IGHV1S21 gene immunoglobulin heavy chain partial sequence | | *Takifugu rubripes* |
| LC000700.1 | IGHV1S4 gene immunoglobulin heavy chain partial sequence | | *Takifugu rubripes* |
| LC000714.1 | IGHV1S18 gene immunoglobulin heavy chain partial sequence | | *Takifugu rubripes* |
| LC000711.1 | IGHV1S15 gene immunoglobulin heavy chain partial sequence | | *Takifugu rubripes* |
| LC000710.1 | IGHV1S14 gene immunoglobulin heavy chain partial sequence | | *Takifugu rubripes* |
| LC000708.1 | IGHV1S12 gene immunoglobulin heavy chain partial sequence | | *Takifugu rubripes* |
| LC000704.1 | IGHV1S8 gene immunoglobulin heavy chain partial sequence | | *Takifugu rubripes* |
| LC000703.1 | IGHV1S7 gene immunoglobulin heavy chain partial sequence | | *Takifugu rubripes* |
| LC000699.1 | IGHV1S3 gene immunoglobulin heavy chain partial sequence | | *Takifugu rubripes* |
| LC000698.1 | IGHV1S2 gene immunoglobulin heavy chain partial sequence | | *Takifugu rubripes* |
| LC000697.1 | IGHV1S1 gene immunoglobulin heavy chain partial sequence | | *Takifugu rubripes* |
| LC000715.1 | IGHV1S19 gene immunoglobulin heavy chain partial sequence | | *Takifugu rubripes* |
| LC000700.1 | IGHV1S4 gene immunoglobulin heavy chain partial sequence | | *Takifugu rubripes* |
| LC000709.1 | IGHV1S13 gene immunoglobulin heavy chain partial sequence | | *Takifugu rubripes* |
| LC000712.1 | IGHV1S16 gene immunoglobulin heavy chain partial sequence | | *Takifugu rubripes* |

**Supplementary Data 1.** computationally predicted 3D structure of Gm-AID used to guide amino acid alignment and as the structure template in ProtASR analyses (Data_S3.pdb).

**Supplementary Data 2.** Aligned nucleotide sequence of genes used in ancestral sequence reconstruction analyses (Data_S2.fas).

**Supplementary Data 3**. The Python code used to assign and RGB color to each AID enzyme based on their catalytic efficiency (Data_S1.ipynb).

**Supplementary Data 4.** The combined scripts, input files, and setting files used to predict ancestral sequences using RAxML, MrBayes, and ProtASR packages (Data_S4.txt).
